# Current advances on the phytochemical composition, pharmacologic effects, toxicology, and product development of *Phyllanthi Fructus*

**DOI:** 10.3389/fphar.2022.1017268

**Published:** 2022-10-19

**Authors:** Xiaoyu Yan, Qiuju Li, Lin Jing, Shuangyue Wu, Wei Duan, Yan Chen, Dayi Chen, Xiaoqi Pan

**Affiliations:** ^1^ School of Public Health, Chengdu University of Traditional Chinese Medicine, Chengdu, China; ^2^ State Key Laboratory of Southwestern Chinese Medicine Resources, Chengdu University of Traditional Chinese Medicine, Chengdu, China; ^3^ School of Pharmacy, Chengdu University of Traditional Chinese Medicine, Chengdu, China

**Keywords:** phyllanthi fructus, traditional Chinese medicine, phytochemistry, pharmacological effects, toxicology, product development

## Abstract

*Phyllanthi Fructus* (PF), the edible fruits of *Phyllanthus emblica* L., serves as an important resource for some health products, foods and drugs due to its high safety and sufficient nutritional value. In recent years, *in vivo* and *in vitro* experiments have been conducted to reveal the active components of PF. More than 180 compounds have been isolated and identified from the PF so far, primarily including tannins, phenolic acids, flavonoids, terpenoids, polysaccharides, fatty acids and amino acids. In traditional Chinese medicine (TCM), PF is used to cure several diseases such as bronchitis, asthma, diabetes, peptic ulcer, hepatopathy, leprosy, and jaundice. Consistent with ethnopharmacology, numerous modern studies have demonstrated that the extracts or monomeric compounds derived from PF exhibit various pharmacological effects including anti-oxidation, anti-bacteria, anti-inflammation, anti-tumour, anti-virus, immunity improvement, hypoglycemic and hypolipidemic effects, and multiple organ protective protection. Toxicological studies on PF indicated the absence of any adverse effects even at a high dose after oral administration. Due to strict quality control, these pharmacological activities and the safety of PF greatly improve the development and utilization of products. Our comprehensive review aims to summarize the phytochemistry, pharmacological effects, toxicology, and product development of PF to provide theoretical guidance and new insights for further research on PF in the future.

## 1 Introduction

One-quarter of the drugs in the world are directly extracted from plants or prepared with plants as raw materials, such as cocaine in coca leaves and digitalis toxin in digitalis leaves (Na and Yang, 2002). The prevalence of medicinal plants in clinical application is well documented in the pharmacopeias of various countries. Over 1,300 kinds of medicinal plants with anti-inflammatory and analgesic properties have been used by European physicians to treat renal and digestive system diseases in clinics (Wang et al., 2020). In addition, the United States has screened more than 20,525 kinds of medicinal plants to discover novel drugs against cancer and acquired immune deficiency syndrome (AIDS) (Xiao and Liu, 1983). Medicinal plants have been used in China for at least 2000 years. According to records, there are 35,784 species of plants in China, with nearly 12,000 species having medicinal value (Wang, 2022).

*Phyllanthus emblica* L., which is also known as amla*,* nelli, anmole, or dhatriphala in different countries, belongs to the Phyllanthaceae family that contains more than 1,000 species widely distributed around the world. And it has been promoted and planted by the United Nations Health Organization throughout the world as three healthy plants, together with *Pinus massoniana* Lamb. and *Pueraria thomsonii* Benth. (Wu et al., 2017). Although all parts of *Phyllanthus Emblica* L. are used for medicinal purposes, the fruits (*Phyllanthi Fructus,* PF) are more widely used in traditional Chinese medicine (TCM), either alone or in combination with other traditional herbs for the treatment of many infectious and non-infectious diseases. PF was listed as a dual-purpose medicinal material by China’s Ministry of Health in 1998 (Wu et al., 2017). In Ayurveda and Unani systems of medicine, PF is also one of the key constituents used in various herbal formulations including patented drugs (Rai et al., 2012). Meanwhile, PF has been used as nutritious tonic, possessing vital amino acids and vitamins. It is particularly the main source of ascorbic acid and minerals compared to other citrus fruits. More than 180 compounds, such as tannins, phenolic acids, flavonoids, fatty acids, polysaccharides, terpenoids, and amino acids, have so far been identified in PF. Various biological activities of these compounds have been reported. Modern pharmacology research has confirmed that PF has many pharmacological effects including anti-inflammation, anti-oxidation, anti-tumour, anti-viral and lowering blood lipid, blood glucose and blood pressure. A recent study reported the role of PF against skin aging (Chaikul et al., 2021). Furthermore, no obvious adverse effects have been observed during PF application, which means that PF presents high security in clinical treatment (Li, 2001).

Previous studies focused on pharmacological activities of PF without providing a comprehensive critical analysis of other relevant information, such as phytochemistry and product development. In this review, a comprehensive understanding of PF as drugs or food, such as phytochemistry, pharmacological effects, toxicology, product application and quality control were summarized. Our review provides scientific evidence for fully exploiting the nutritional value of PF products and developing high-value-added products in depth to promote the sustainable and healthy development of the PF production industry.

## 2 Literature search methods

**FIGURE 1:**
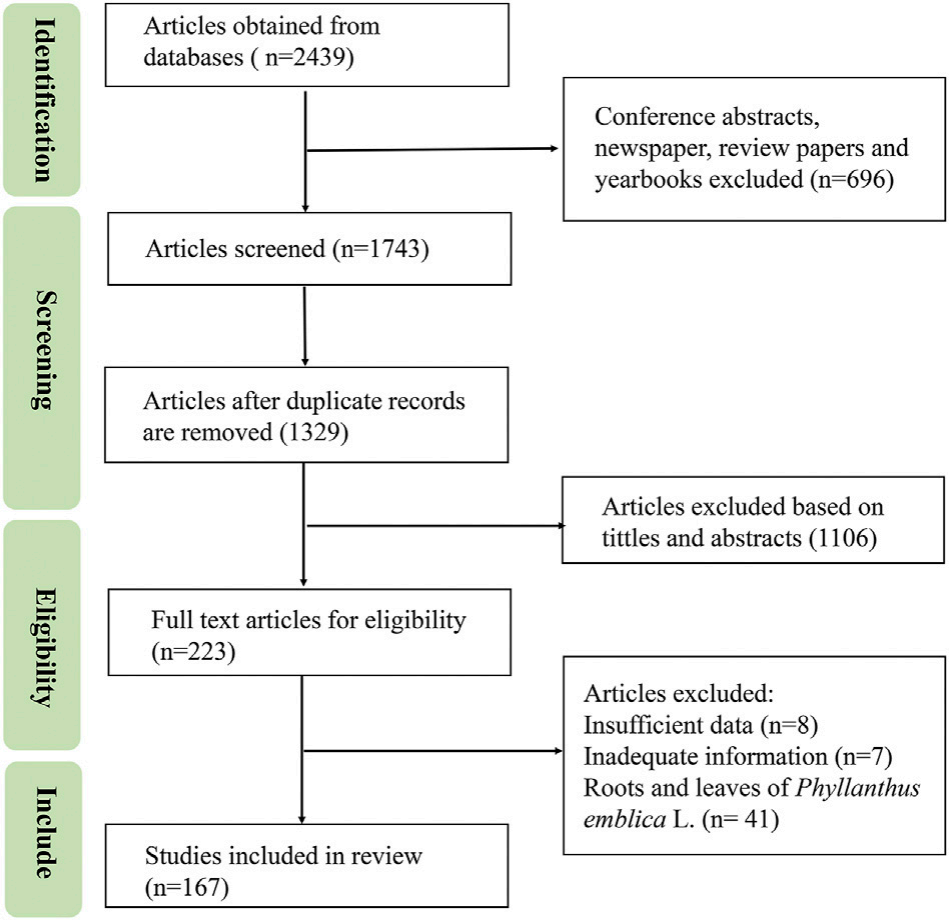
The flow of study selection.

Available information on PF was collected from published materials, including monographs on medicinal plants, ancient and modern recorded classics, pharmacopeias, and electronic databases, such as Web of Science, PubMed, CNKI, Wanfang Database, Baidu Scholar, Flora of China (FOR), from 1952 up to June 2022. The following keywords including “*Phyllanthus* emblica”, “the botany of *Phyllanthus* emblica”, “pharmacological activity of *Phyllanthus* emblica”, “antioxidant of *Phyllanthus* emblica”, “biological activity of *Phyllanthus* emblica”, “quality control of *Phyllanthus* emblica”, “traditional uses of *Phyllanthus* emblica”, or “toxicity of *Phyllanthus* emblica” were used to search relevant literature. The titles and abstracts were screened for relevance, and articles related to roots and leaves of *Phyllanthus emblica* L., or repetitive articles were eliminated. *in vitro* or *in vivo* studies and clinical trials of PF were included. The search strategy is shown in Figure 1. The IUPAC names of the known chemical compounds were checked using the PubChem database and their chemical structures were drawn using ChemDraw Pro 20.0 software.

## 3 Botanical description and geographic distribution

PF, the ripe fruit of *Phyllanthus Emblica* L., is recorded in various versions of Chinese Pharmacopoeia. The plant is easy to adapt to the external environment and climate, which often grows in sparse forests, scrub, wastelands or sunny places in ravines at 200–2,300 m above sea level (Yang et al., 2018). The size of the tree is small to medium with a range of 8–18 m in height (Ganesan, 2003). The flowers are greenish-yellow. Flowering between April and June (Wei et al., 2002; Arora et al., 2012). The fruit is nearly spherical, diameter ranges between 1.8 and 2.5 cm, light greenish yellow or brick-red, quite smooth and hard on appearance, with six vertical stripes or furrows (Sangeetha et al., 2010). The fruiting period is from July to September. The fruit, as a highly nutritious drug-food homologous substance, has a sweet and sour taste. It is often divided into wild and fruity types in the market. And the fruity types are characterized by larger and sweeter fruits, smaller kernels, less fiber and higher yields compared with the wild types. Fresh fruit is often processed as jam, preserved, and canned fruit. The seeds are slightly reddish, 5–6 mm long, and 2–3 mm wide (Flora of China Editorial Committee, 1998). The seedlings start bearing fruits 7–8 years after planting, while the budded clones start bearing fruits after 5 years (Kumar et al., 2012).

PF is distributed in most tropical and subtropical countries mainly in China, Indonesia, and the Malay Peninsula. PF is native to tropical southeastern Asia, particularly in central and southern India, Pakistan, Nepal, Bhutan, Bangladesh, Malaya, Myanmar and the Mascarene Islands (Supplementary Figure S1) (Xia et al., 1997). Originally, it was cultivated in Madagascar (Ganesan, 2003). Subsequently, PF is also cultivated in South America. In China, PF is widely growing in Sichuan, Guizhou, Jiangxi, Fujian, Guangdong, Hainan, Taiwan, Guangxi and Yunnan provinces (Supplementary Figure S2) (Yang et al., 2014). The fruit from Guizhou and Yunnan provinces are most suitable for use as medicine and health products, which contain high levels of gallic acid, corilagin and ascorbic acid (Mao, 2019).

## 4 Phytochemical composition of *Phyllanthi Fructus*

PF, like most botanical medicines, contains numerous natural products with different structural patterns, such as tannins, phenolic acids, flavonoids, and ascorbic acid, which are the main active components in pharmacodynamic activity (Pareek et al., 2018; Zhu et al., 2018). Isocorilagin (27), emblicanin A (44) and B (45), *etc.* are unique phytochemicals of PF with antioxidant and anti-tumour properties. Major chemical components isolated from PF are listed in Table 1. Different pharmacological activities of PF are often attributed to these active ingredients.

**TABLE 1:** Major chemical components isolated and structurally identified from PF.

NO.	Chemical constituents	Molecular formula	Extracts	Biological activities	References
**Tannins**					
1	Chebulinic acid	C_41_H_32_O_27_	Methanol	Anti-inflammatory, anti-hypertension	Avula et al., 2013; Lu et al., 2020
2	Neochebulic acid	C_41_H_32_O_28_	Methanol	Antioxidant	Avula et al. (2013)
3	1.2,3,6-tetra-*O*-galloyl-*β*-d-glucose	C_34_H_28_O_22_	Methanol	Antioxidant	Xu et al. (2016)
4	1.2,4,6-tetra-*O*-galloyl-*β*-d-glucose	C_34_H_28_O_22_	Ethanol	Anti-virus	Xiang et al. (2011); Yang et al. (2010)
5	3,4,6-tri-*O*-galloyl-d-glucose	C_27_H_24_O_18_	Methanol	Antioxidant	Xu et al. (2016)
6	1,2,3,4,6-penta-*O*-galloyl-*β*-glucose	C_41_H_32_O_26_	Methanol	Antioxidant	Xu et al. (2016)
7	1-*O*-digalloyl-*β*-D-glucos	C_14_ H_18_ O_10_	Aqueous	Antioxidant	Majeed et al. (2009)
8	1-O-galloyl-β-d-glucose	C_13_H_15_O_10_	Ethanol	Antioxidant	Zhang et al. (2001)
9	1,6-di-*O*-galloyl-*β*-d-glucose^a^	C_20_H_20_O_14_	Aqueous	Antioxidant	Majeed et al. (2009)
10	Terchebin	C_41_H_30_O_27_	Ethanol	Antioxidant, anti-bacteria	Zhang et al. (2001)
11	Mallotusinin	C_41_H_26_O_25_	Methanol	Antioxidant, anti-inflammatory	Luo et al. (2012)
12	Isomallotusinin	C_41_H_26_O_25_	Methanol	Antioxidant	Luo et al. (2012)
13	Phyllanemblinin A^a^	C_27_H_20_O_17_	Ethanol	**_**	Yang et al. (2020)
14	Methyl chebulagate	C_42_H_34_O_28_	Ethanol	**_**	Yang et al. (2020)
15	Dimethyl neochebulagate	C_42_H_34_O_28_	Methanol	**_**	Yang et al. (2020)
16	Elaeocarpusin	C_47_H_34_O_32_	Methanol	Antioxidant, anti-inflammatory	Xu et al., 2016; Bobasa et al., 2021
17	Neochebulagic acid	C_41_H_32_O_28_	Methanol	Antioxidant	Xu et al. (2016)
18	Chebulagic acid	C_41_H_30_O_27_	Methanol	Antioxidant, anti-inflammatory, anti-virus	Xu et al., 2016; Du et al., 2021
19	Putranjivain A	C_46_H_36_O_31_	Methanol	Antioxidant, anti-inflammatory, anti-virus	Liang et al., 2010; EL-Mekkawy et al., 1995
20	Putranjivain B	C_53_H_44_O_36_	Methanol	Anti-inflammatory	Yang and Liu, (2014)
21	Punicafolin	C_41_H_30_O_26_	Methanol	Anti-diabetic	Xu et al. (2016)
22	Carpinusnin	C_41_H_34_O_29_	Methanol	**_**	Xu et al. (2016)
23	Mallonin	C_33_H_28_O_24_	Methanol	**_**	Xu et al. (2016)
24	Geraniin	C_41_H_28_O_27_	Methanol	Antioxidant, immunomodulatory, anti-tumour	Xu et al., 2016; Liu et al., 2008
25	Tercatain	C_34_H_26_O_22_	Methanol	Anti-inflammatory	Xu et al., 2016; Yang and Liu, 2014
26	Corilagin	C_27_H_22_O_18_	Methanol	Antioxidant, immunomodulatory, anti-tumour	Luo et al. (2012)
27	Isocorilagin^a^	C_27_H_22_O_18_	Methanol	Antioxidant	Liu et al. (2008)
28	Furosin	C_27_H_22_O_19_	Methanol	Antioxidant	Liu et al. (2008)
29	Chebulanin	C_27_H_24_O_19_	Methanol	Anti-inflammatory	Luo et al. (2012)
30	2.3-(*S*)-hexahydroxy-diphen-oyl-d-glucose	C_20_H_33_O_22_	Ethanol	**_**	Xu, (2009)
31	MLT-1	C_69_H_47_O_42_	Ethanol	**_**	Xu, (2009)
32	MLT-4	C_48_H_35_O_30_	Ethanol	**_**	Xu, (2009)
33	Tuberonic acid glucoside	C_18_H_28_O_9_	Ethanol	Anti-diabetic	Xu, (2009)
34	1(*β*),4-di-*O*-galloyglucose	C_20_H_20_O_14_	Ethanol e	**_**	Xu, (2009)
35	3,4,6-tri-*O*-galloyl-*β*-d-glucose	C_27_H_24_O_18_	Aqueous	Antioxidant, anti-bacteria	Bobasa et al. (2021)
36	Phyllanthunin	C_32_H_30_	Ethanol	**_**	Yang et al. (2007)
37	2-(2-methylbutyryl)-phloroglucinol-1-*O*-*β*-d-glucopyranoside	C_15_H_20_O_10_	Ethanol e	Antioxidant	Xu, 2009; Wu et al., 2017
38	2-(2-methylbutyryl)-phloroglucinol-1-*O*-(6″-*O*-*β*-d-apiofuranosyl)-*β*-d-glucopyranoside	C_20_H_27_O_13_	Ethanol	_	Zhang et al. (2002)
39	Gallic acid-3-*O*-(6′-O-galloyl)-*β*-d-glucose^a^	C_20_H_20_O_14_	Ethanol	Antioxidant	Xu, (2009)
40	3.3′-dimethylellagic acid -4′-*O*-*α*-l- rhamnopyranoside	C_30_H_32_O_12_N_1_B_1_	Ethanol	**_**	Xu, (2009)
41	3-*O*-methylellagicacid-4′-*O*-α-L-rha-mnopyanoside	C_22_H_20_O_12_	Ethanol	**_**	Xu, (2009)
42	Isostrictiniin	C_27_H_22_O_18_	Ethanol	Hepatoprotective activity	Zhou et al. (2018)
43	Chebulic acid	C_14_H_12_O_11_	Ethanol	Anti-neuroinflammatory, antioxidant	Xu et al. (2016)
44	Emblicanin A^a^	C_34_H_22_O_22_	Methanol	Antioxidant	Bhattacharya et al. (2000a)
45	Emblicanin B^a^	C_34_H_20_O_22_	Methanol	Antioxidant	Bhattacharya et al. (2000b)
46	Prodelphinidin B2	C_30_H_26_O_14_	Ethanol	Anti-tumour	Zhou et al. (2018)
47	Epicatechin-(4*β*→8)-epigallo-catechin-3-*O*-gallate^a^	C_37_H_30_O_17_	Ethanol	_	Xu, (2009)
48	Epicatechin-(4*β*→8)-gallocatechin	C_30_H_26_O_13_	Acetone	Hepatoprotective effects	Xu, (2009)
49	Prodelphinidin B1	C_30_H_26_O_14_	Ethanol	Anti-tumour	Zhou et al. (2018)
50	Epicatechin-(2*β*→7,4*β*→8)-gallocatechin^a^	C_30_H_22_O_14_	Ethanol	_	Xu, (2009)
**Phenolic acids**					
51	Mucic acid	C_6_H_10_O_8_	Aqueous	Antioxidant	Majeed et al. (2009)
52	Mucic acid-1-methyl ester-6-ethyl ester	C_9_H_15_O_8_	Ethanol	Antioxidant	Zhang et al. (2016)
53	Mucic acid-1-methyl ester-2-*O*-gallate	C_14_H_20_O_12_	Methanol	Antioxidant	Zhang et al. (2017)
54	Mucic acid-6-methyl ester-2-*O*-gallate	C_14_H_20_O_12_	Methanol	Antioxidant, anti-virus	Zhang et al. (2017)
55	Mucic acid dimethyl ester-2-*O*-gallate	C_16_H_22_O_15_	Aqueous	Antioxidant, anti-virus	Xu, (2009)
56	Mucic acid-2-*O*-gallate^a^	C_13_H_14_O_13_	Ethanol	_	Xu et al., 2016
57	Mucic acid 2,5-di-*O*-gallate	C_20_H_25_O_16_	Ethanol	_	Olennikov et al. (2015)
58	l-malic acid-2-*O*-gallate^a^	C_13_H_14_O_13_	Aqueous	Antioxidant, anti-tumour	Xu, (2009)
59	Mucic acid-1,4-lactone-3-*O*-gallate^a^	C_14_H_12_O_11_	Methanol	Antioxidant	Xu, 2009; Luo et al., 2012
60	Mucic acid-1,4-lactone-6-methyl ester-2-*O*-gallate	C_15_H_14_O_11_	Ethanol	Antioxidant	Xu, 2009; Liu et al., 2017
61	Mucic acid-1,4-lactone-5-*O*-gallate^a^	C_14_H_12_O_11_	Ethanol	Antioxidant	Xu, (2009)
62	Mucic acid-1,4-lactone-3,5-di-*O*-gallate^a^	C_21_H_16_O_15_	Acetone	_	Xu, 2009; Zhang et al., 2001
63	Mucid acid-1,4-lactone-2-*O*-gallate	C_14_H_12_O_11_	Ethanol	Antioxidant	Xu, (2009)
64	Mucic acid 1,4-lactone-6-methyl-ester-5-*O*-gallate	C_15_H_14_O_11_	Acetone	_	Xu, 2009; Zhang et al., 2001
65	Mucic acid 2,5-di-*O*-gallate 1,4-lactone	C_21_H_16_O_15_	Acetone	Antioxidant	Olennikov et al. (2015)
66	Pyrogallol	C_6_H_6_O_3_	Ethanol	Antioxidant	Xu, (2009)
67	1,3,5-trihydroxybenzene-1-*O*-galloyl-*β*-D-glucoseide	C_12_H_16_O_9_	Ethanol	_	Xu, (2009)
68	Gallic acid	C_7_H_6_O_5_	Methanol	Antioxidant, anti-inflammatory	Xu et al., 2009
69	Protocatechuic acid	C_7_H_6_O_4_	Methanol	Anti-inflammatory	Xu, (2009)
70	Methyl gallate	C_8_H_8_O_5_	Methanol	Hypoglycemic activity	Xu, 2009; Liu et al., 2017
71	Ethyl gallate	C_9_H_10_O_5_	Ethanol	Antioxidant	Zhang et al. (2016)
72	3-ethoxy-4,5-dihydroxy-benzoic acid^a^	C_14_H_10_O_6_	Acetone	_	Zhang et al. (2003)
73	2-carboxylmethylphenol-1-*O*-*β*-d-glucopyranoside^a^	C_13_H_18_O_9_	Acetone	_	Zhang et al. (2003)
74	2,6-dimethoxy-4-(2-hydroxyethyl)-phenol-1-*O*-*β*-d-glucopyranoside^a^	C_16_H_24_O_10_	Acetone	_	Zhang et al. (2001)
75	Chebulic acid trimethyl ester	C_17_H_20_BO_14_	Ethanol	Hepatoprotective effects, antioxidant	Yang et al. (2020)
76	3.3′-dihydroxy-4,4′-2-propenyl-2,2′-biphenyldicarboxylic acid	C_20_H_18_O_6_	Ethanol	Antioxidant	Yang et al. (2020)
77	3,4,8,9,10-pentahydroxydibenzo [b,d] pyran-6-one	C_13_H_8_O_7_	Ethanol	_	Zhang et al. (2016)
78	1*β*,6-di-*O*-galloylglucose	C_20_H_20_O_14_	Ethanol	_	Nisar et al. (2018)
79	Digallic acid	C_14_H_10_O_9_	Methanol	Antioxidant, lipid-lowering activity	Xu et al. (2016)
80	Ellagic acid	C_14_H_6_O_8_	Acetone	Antioxidant, anti-inflammatory	Xu et al. (2016)
81	Aryl-tetralin-type lignan^a^	C_31_H_33_O_15_	Ethanol	_	Yang et al. (2020)
**Flavonoid**					
82	Eriodictyol	C_15_H_12_O_6_	Methanol	Antioxidant, anti-tumour	Xu et al. (2016)
83	Eriodictyol-7-*O*-gluceside	C_21_H_22_O_11_	Methanol	_	Xu et al. (2016)
84	(S)-eriodictyol-7-*O*-(6″-*O*-galloyl)-*β*-d-glucopyranoside	C_22_H_17_O_10_	Ethanol	_	Zhang et al. (2002)
85	(-)-epigallocatechin-3-*O*-gallate	C_22_H_18_O_11_	Methanol	Antioxidant, anti-inflammatory	Xu et al. (2016)
86	(-)-epicatechin-3-*O*-gallate	C_22_H_18_O_10_	Methanol	Antioxidant, anti-inflammatory	Xu et al. (2016)
87	(-)-epiafzelechin	C_15_H_14_O_5_	Methanol	Antioxidant	Xu et al. (2016)
88	(-)-epigallocatechin	C_15_H_14_O_7_	Methanol	Antioxidant, anti-virus	Xu et al. (2016)
89	(-)-epicatechin	C_15_H_14_O_6_	Methanol	Antioxidant	Xu et al. (2016)
90	(-)-gallocatechin	C_15_H_14_O_7_	Methanol	Antioxidant, anti-inflammatory	Xu et al. (2016)
91	(+)-catechin	C_15_H_14_O_6_	Ethanol	Antioxidant, anti-inflammatory	Wang et al. (2019)
92	Delphinidin	C_15_H_11_ClO_7_	Methanol	Antioxidant	Xu et al., 2009
93	Wogonin	C_16_H_12_O_5_	Methanol	Antioxidant, anti-tumour	Nisar et al. (2018)
94	5,7-dihydroxy-2-(4-hydroxyphenyl)-4H-chromen-4-one	C_15_H_10_O_5_	Methanol	Antioxidant, anti-tumour	Xu et al., 2009
95	Naringenin	C_15_H_12_O_5_	Ethanol	Anti-inflammatory	Wang et al. (2019)
96	Naringenin-7-*O*-glucoside	C_21_H_22_O_10_	Methanol	Hypoglycemic activity	Xu et al. (2016)
97	Naringenin-7-*O*-(6″-*O*-galloyl)-gluceside	C_28_H_26_O_17_	Ethanol	_	Xu et al. (2016)
98	Dihydrokaempferol	C_15_H_12_O_6_	Methanol	Antioxidant, anti-inflammatory	Xu et al. (2016)
99	Rutin	C_27_H_30_O_16_	Methanol	Antioxidant, anti-tumour	Xu et al. (2016)
100	Kaempferol	C_15_H_10_O_6_	Aqueous	Antioxidant, anti-inflammatory	Cui et al. (2010)
101	Kaempferol 3-*O*-rhamninoside	C_33_H_40_O_19_	Aqueous	_	Cui et al. (2010)
102	Quercetin	C_15_H_10_O_7_	Aqueous	Antioxidant, anti-inflammatory, antiviral, anti-tumour	Liu et al. (2007b)
103	Myricetin-3-*O*-rhamnoside	C_21_H_20_O_12_	Methanol	Antioxidant	Xu et al. (2016)
104	Quercetin-3-rhamnoside	C_21_H_20_O_11_	Aqueous	_	Liu et al. (2007a)
105	Isoquercitrin	C_21_H_20_O_12_	Aqueous	_	Liu et al. (2007b)
106	Quercetin-7-*O*-*β*-d-glucopyranoside	C_21_H_20_O_12_	Aqueous	Antioxidant	Liu et al. (2007a)
107	Myricetin	C_15_H_10_O_8_	Aqueous	Antioxidant	Liu et al. (2007b)
108	2-(2-methylbutyryl) phloroglucinol (multifidol)glucoside^a^	C_16_H_25_O_10_	Methanol	_	Xu et al. (2016)
109	2-(2-methylbutyryl) phloroglucinol-1-O-(6″-O-β-d-apiofuranosyl)-β-d-glucopyranoside^a^	C_22_H_32_O_13_	Methanol	_	Xu et al. (2016)
110	Avicularin	C_20_H_18_O_11_	Ethanol	_	Zhou et al. (2018)
111	Naringenin-7-*O*-(6″-*O*-trans-pcoumaroyl)-gluceside	C_30_H_28_O_12_	Methanol	_	Xu, (2009)
112	(S)-eriodictyol-7-*O*-(6″-*O*-trans-pcoumaroyl)-*β*-d-glucopyranoside^a^	C_29_H_29_O_13_	Ethanol	_	Zhang et al. (2002)
**Terpenoids**					
113	*β*-amyrin	C_30_H_50_O	Ethanol	Anti-inflammatory, anti-virus	Zhou et al. (2018)
114	Betulin	C_30_H_50_O_2_	Ethanol	Anti-inflammatory, anti-virus	Zhou et al. (2018)
115	*β*-amyrone	C_30_H_48_O	Ethanol	Antiviral	Zhou et al. (2018)
116	Lupeol	C_30_H_50_O	Methanol	Anti-inflammatory, anti-virus	Xu et al. (2016)
117	Phyllaemblinol^a^	C_20_H_24_O_9_	Ethanol	Antioxidant	Zhang et al. (2016)
**Polysaccharides**					
118	Pectin	C_5_H_10_O_5_	Methanol	Antioxidant, anti-aging	Wang et al. (2019)
119	Glucose	C_6_H_12_O_6_	Methanol	Antioxidant, anti-aging	Wang et al. (2019)
120	Fructose	C_6_H_12_O_6_	Methanol	Antioxidant, anti-aging	Wang et al. (2019)
121	Saccharose	C_12_H_22_O_11_	Methanol	Antioxidant, anti-aging	Wang et al. (2019)
**Fatty acids**					
122	Lauric acid	C_12_H_24_O_2_	Ethanol	Immuno-enhancement, anti-virus	Kumaran and Karunakaran, (2006)
123	Palmitic acid	C_16_H_32_O_2_	Ethanol	Antioxidant	Xu, (2009)
124	Hexacosanoic acid	C_26_H_52_O_2_	Ethanol	_	Xu, (2009)
125	Palmitoleic acid	C_16_H_30_O_2_	Ethanol	Hypoglycemic, hypolipidemic activities	Zhao et al. (2007)
126	Heptadecanoic acid	C_₁7_H_₃6_O_2_	Ethanol	_	Zhao et al. (2007)
127	Oleic acid	C_18_H_34_O_2_	Ethanol	Hypoglycemic, hypolipidemic activities	Zhao et al. (2007)
128	Stearic acid	C_18_H_36_O_2_	Ethanol	_	Zhao et al. (2007)
129	Linolenic acid (a.α-linolenic acid, b. γ-linolenic acid)	C_18_H_30_O_2_	Ethanol	Hypoglycemic, hypolipidemic activities and antioxidant	Zhao et al. (2007)
130	Myristic acid	C_14_H_28_O_2_	Ethanol	Anti-tumour, anti-bacteria	Zhao et al. (2007)
131	Arachidonic acid	C_20_H_32_O_2_	Ethanol	Immuno-enhancement, antioxidant	Zhao et al. (2007)
132	Docosanoic acid/Behenic acid	C_22_H_44_O_2_	Ethanol	_	Meng et al. (2011)
133	Pentacosanoic acid	C_50_H_100_O_2_	Ethanol	_	Zhao et al. (2007)
134	Melissic acid	C_30_H_60_O_2_	Ethanol	Anti-bacteria	Zhao et al. (2007)
135	Lignoceric acid	C24H48O2	Ethanol	_	Meng et al. (2011)
**Amino acids**					
136	Glutamic acid	C₅H₉NO₄	Aqueous	Regulation of protein and glucose metabolism	Yuan et al. (2021)
137	Proline acid	C_5_H_9_NO_2_	Aqueous	Immunomodulatory effect	Yuan et al. (2021)
138	Alanine acid	C3H7NO2	Aqueous	Hypoglycemic, hypolipidemic activities, and immuno-enhancement	Yuan et al. (2021)
139	Lysine acid	C_6_H_14_N_2_O_2_	Aqueous	Neuroprotective effect	Yuan et al. (2021)
140	Tryptophan acid	C_11_H_12_N_2_O_2_	Aqueous	Neuroprotective effect	Yuan et al. (2021)
141	Histidine acid	C_6_H_9_N_3_O_2_	Aqueous	Antioxidant and anti-inflammatory	Yuan et al. (2021)
142	Tyrosine acid	C_9_H_11_NO_3_	Aqueous	Hypoglycemic activity	Wu et al. (2003)
143	Leucine acid	C_6_H_13_NO_2_	Aqueous	Hypoglycemic activity	Wu et al. (2003)
144	Methionine acid	C_5_H_11_NO_2_S	Aqueous	Immunomodulatory effect	Wu et al. (2003)
145	Isoleucine acid	C_6_H_13_NO_2_	Aqueous	Hypoglycemic activity	Wu et al. (2003)
146	Arginine acid	C_6_H_14_N_4_O_2_	Aqueous	Immuno-enhancement	Wu et al. (2003)
147	Serine acid	C_3_H_7_NO_3_	Aqueous	Immunomodulatory effect	Wu et al. (2003)
148	*Glycine* acid	C_2_H_5_NO_2_	Aqueous	Immunomodulatory and Neuroprotective effect	Wu et al. (2003)
149	Threonine acid	C_4_H_9_NO_3_	Aqueous	Immunomodulatory effect and hypolipidemic activity	Wu et al. (2003)
150	Phenylalanine acid	C_9_H_11_NO_2_	Aqueous	Hypoglycemic activity and antioxidant	Wu et al. (2003)
**Other compounds**					
151	Vitamin B_1_	C_12_H_17_ClN_4_OS	Aqueous	Hypoglycemic activity and neuroprotective effect	Chen et al. (2003)
152	Vitamin B_2_	C_17_H_20_N_4_O_6_	Aqueous	Regulation of metabolism	Chen et al. (2003)
153	Ascorbic acid	C_6_H_8_O_6_	Methanol	Antioxidant and anti-inflammatory	Chen et al. (2003)
154	α-carotene	C_40_H_56_	Methanol	Anti-tumour	Wang et al. (2019)
155	β-carotene	C_40_H_56_	Methanol	Immuno-enhancement and antioxidant	Wang et al. (2019)
156	Nicotinic acid	C_6_H_5_NO_2_	Ethanol	Anti-glycation and skin Protection	Wang et al. (2019)
157	24-propylcholesterol	C_29_H_50_O	Ethanol	Hypoglycemic activity, antioxidant, anti-inflammatory and anti-tumour	Kumaran and Karunakaran, (2006)
158	Sitosterol	C_29_H_48_O	Ethanol	Antioxidant, anti-inflammatory and anti-tumour	Xu et al. (2016)
159	β-sitosterol-D-glucoside	C_35_H_60_O_6_	Ethanol	Antioxidant and anti-inflammatory	Xu et al. (2016)

^a^ This compound is unique to PF.

### 4.1 Tannins

**FIGURE 2:**
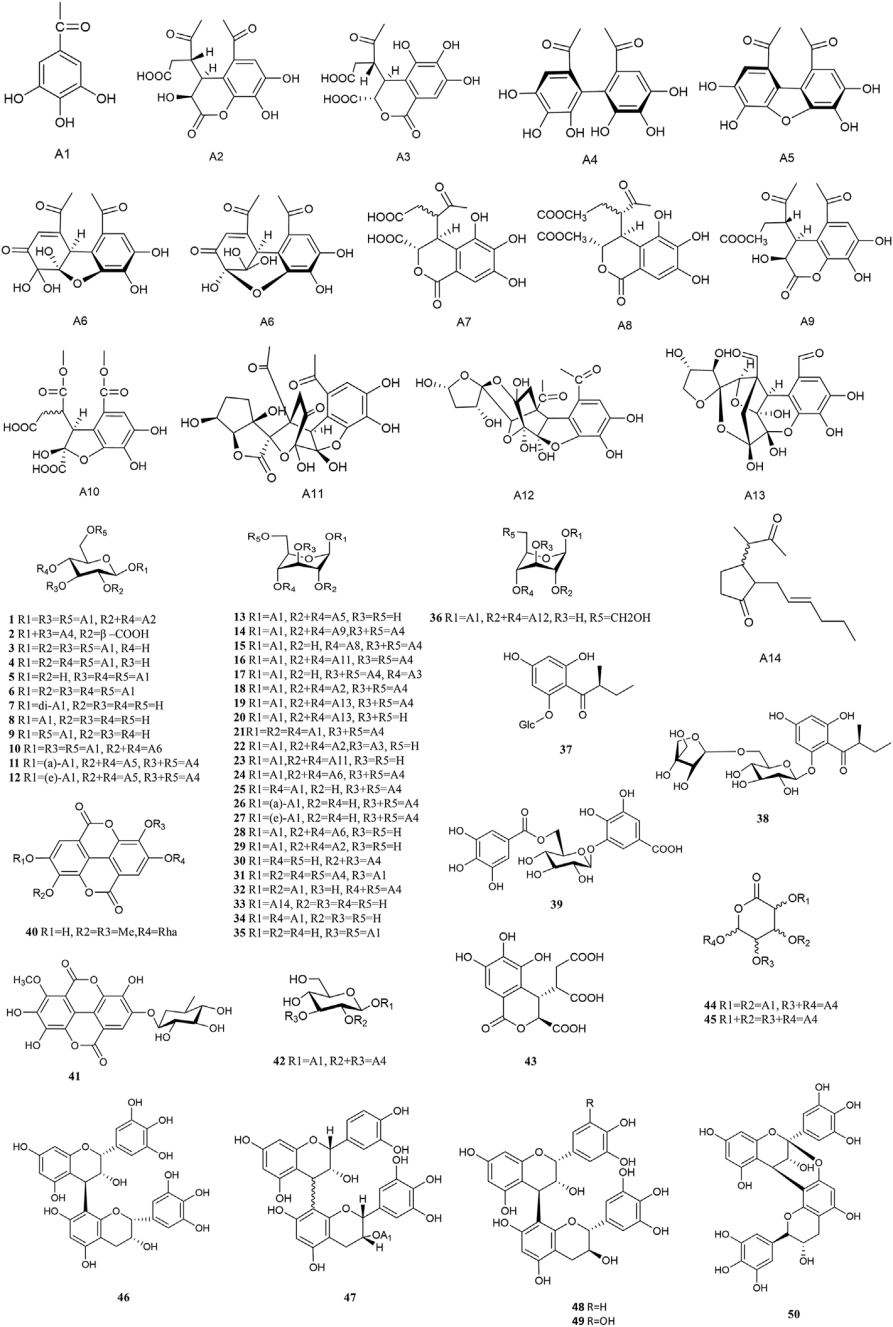
Chemical structures of Tannins (1–50).

Tannins are a kind of polyphenolic compounds with a complex structure, whose molecular structure has many active phenolic hydroxyl groups. These active phenolic hydroxyl groups are easily oxidised into quinone structures which can scavenge free radicals, thus rendering their antioxidant, anti-inflammatory, anti-mutagenic and anti-cancer activities (Shao et al., 2014). According to the chemical structure, tannins include hydrolyzed and condensed types (Guo, 2013). Tannins content is up to 45% and 14% in fresh and dried PF respectively, most of which are hydrolyszable tannins (Chen et al., 2016). Tannin compounds mainly take d-glucose as glycosyl, and various monomers are derived at the substitutions of R1, R2, R3, R4 and R5, respectively. Approximately 49 types of tannins have been isolated from PF thus far, including chebulinic acid (1), elaeocarpusin (15), chebulagic acid (17), and geraniin (23), etc (Figure 2). 3,4,6-tri-*O*-galloyl-*β*-d-glucose was isolated from PF for the first time in 2015 (Olennikov et al., 2015). Methyl chebulagate (13) and dimethyl neochebulagate (14) as novel compounds isolated from PF have not been fully studied in terms of pharmacological activity (Yang et al., 2020).

### 4.2 Phenolic acids

**FIGURE 3:**
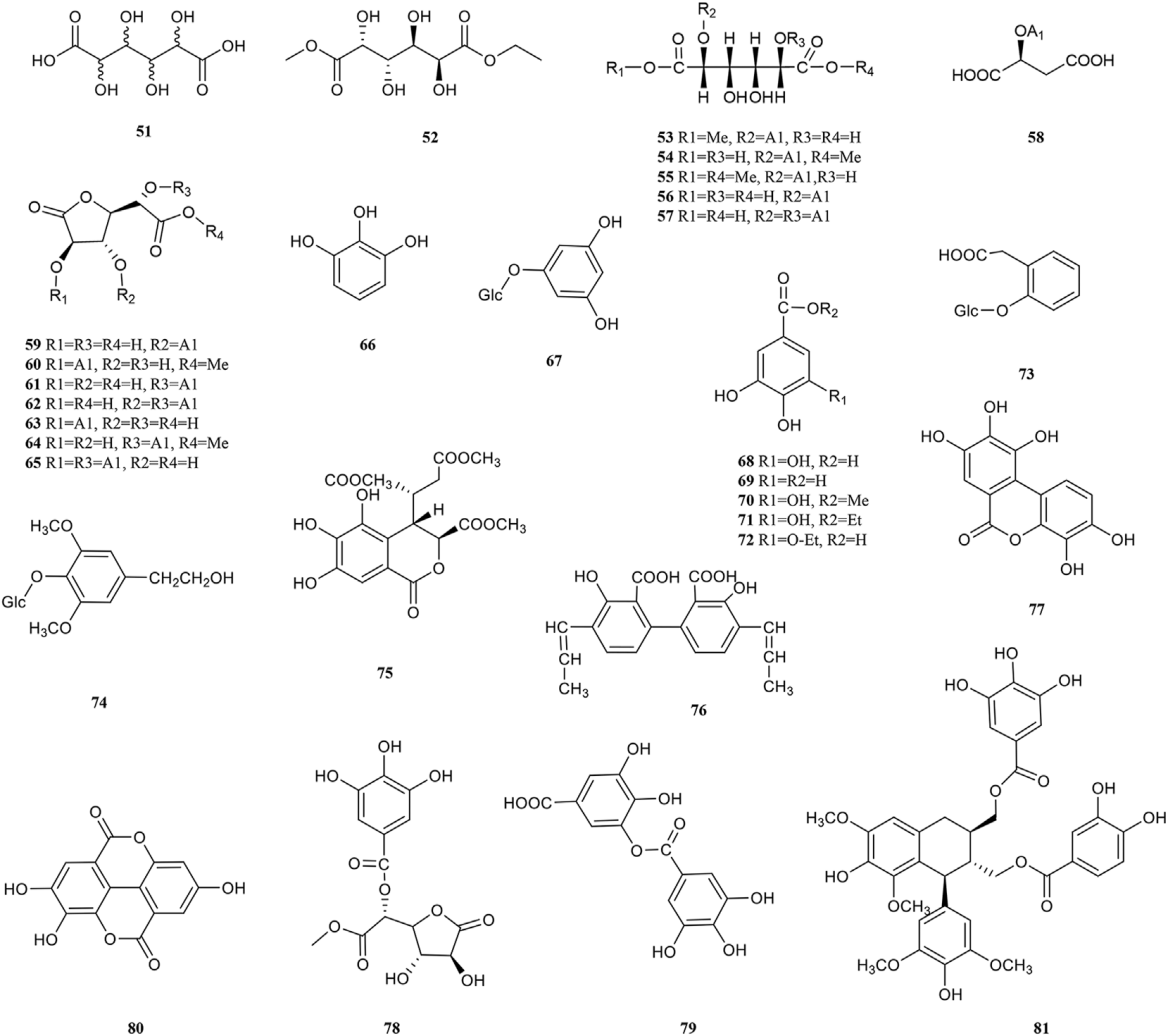
Chemical structures of phenolic acids (51–81).

Phenolic acids are generated by replacing a hydrogen atom in an aromatic ring with a carboxylic acid group and at least one hydroxyl group. On the one hand, the hydrogen atoms given by the breakage of the -O-H bond of the phenolic hydroxyl group can bind directly to free radical molecules. On the other hand, phenolic compounds can be converted into polyphenol anions by ionizing H^+^ in polar solvents or under alkaline conditions, which converts oxygen radicals directly into anions (Chen, 2019). Therefore, phenolic acids have a wide range of physiological activities, such as antioxidant, anti-UV radiation, anti-bacterial and anti-viral effects, which are used in food additives, pharmaceuticals, and cosmetic ingredients. 30 phenolic acid (51–81) components have been isolated from PF (Figure 3). Olennikov et al. isolated two new mucic acid derivatives, mucic acid 2,5-di-*O*-gallate 56) and mucic acid 2,5-di-*O*-gallate1,4-lactone (63), both of which have antioxidant activity (Olennikov et al., 2015). Chebulic acid trimethyl ester (72) and aryl-tetralin-type lignan (77) in PF were first identified in 2020. (Yang et al., 2020).

### 4.3 Flavonoids

**FIGURE 4:**
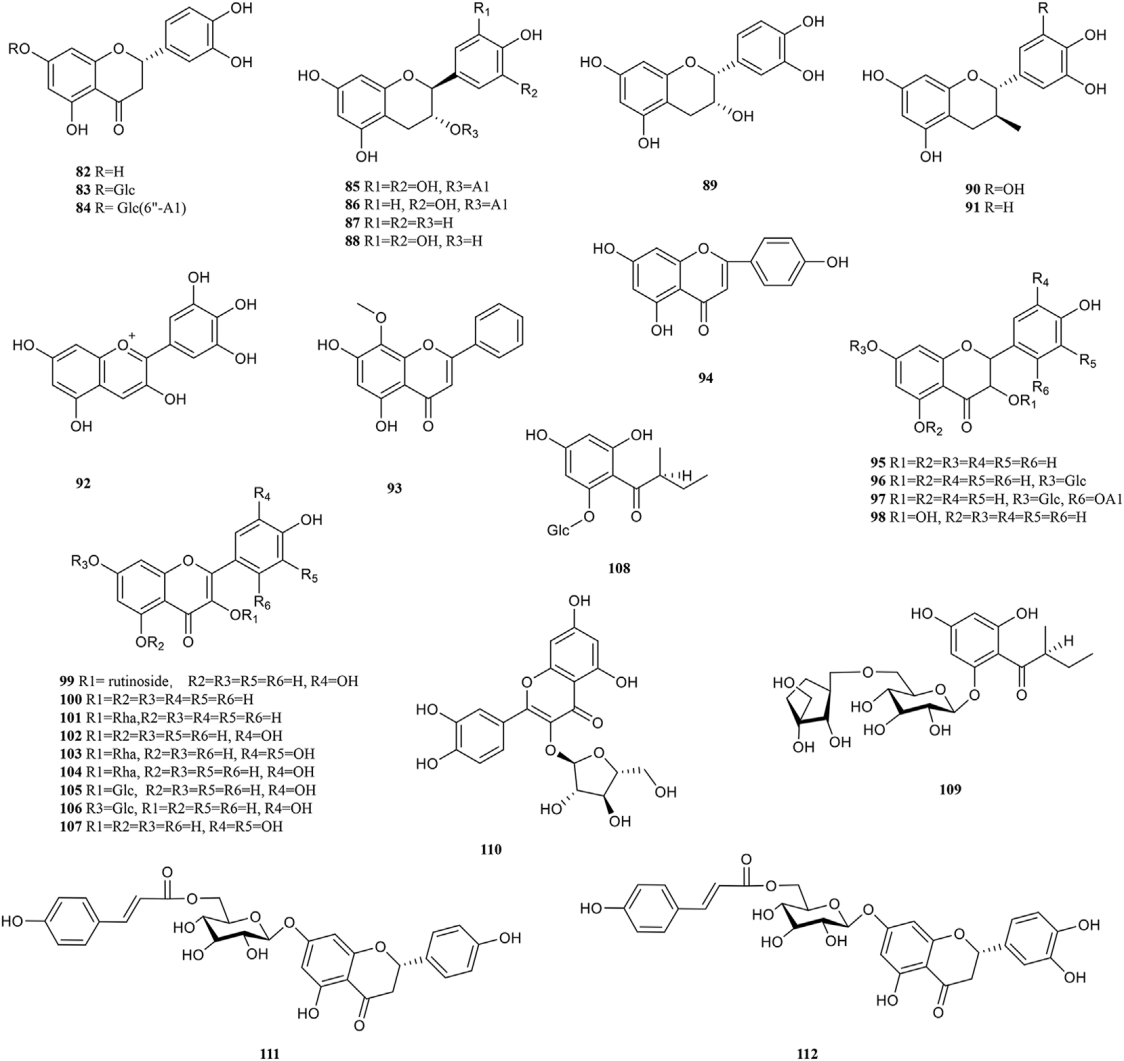
Chemical structures of Flavonoids (82–112).

Flavonoids are formed by two benzene rings with hydroxyl groups attached to the A and B ring linked *via* three central carbon atoms, thus having 2-benzylchromone basic parent nucleus structures. The biological activity of flavonoids is closely related to the chemical structure. Flavonoids terminate free radical chain reactions by binding to peroxyl radicals, which is one of the reasons for the antioxidant properties. The antioxidant activity is also related to the following structural features, the substitution position and number of phenolic hydroxyl groups, the C-2,3 double bond, and the spatial structure of the compound (Chen et al., 2013). The antitumour activity of flavonoids is attributed to the difference in parent nucleus structure, hydroxylation pattern and degree, and whether the hydroxyl group is substituted or not (Zhao et al., 2015). In addition, its anti-inflammatory and antiviral activity is influenced by the above-mentioned constitutive relationships. So far, over 30 flavonoids (82–112) have been reported in PF (Figure 4). Wogonin (93), rutin (99), and quercetin (102) are typical representatives of the flavonoids in PF.

### 4.4 Terpenoids

**FIGURE 5:**
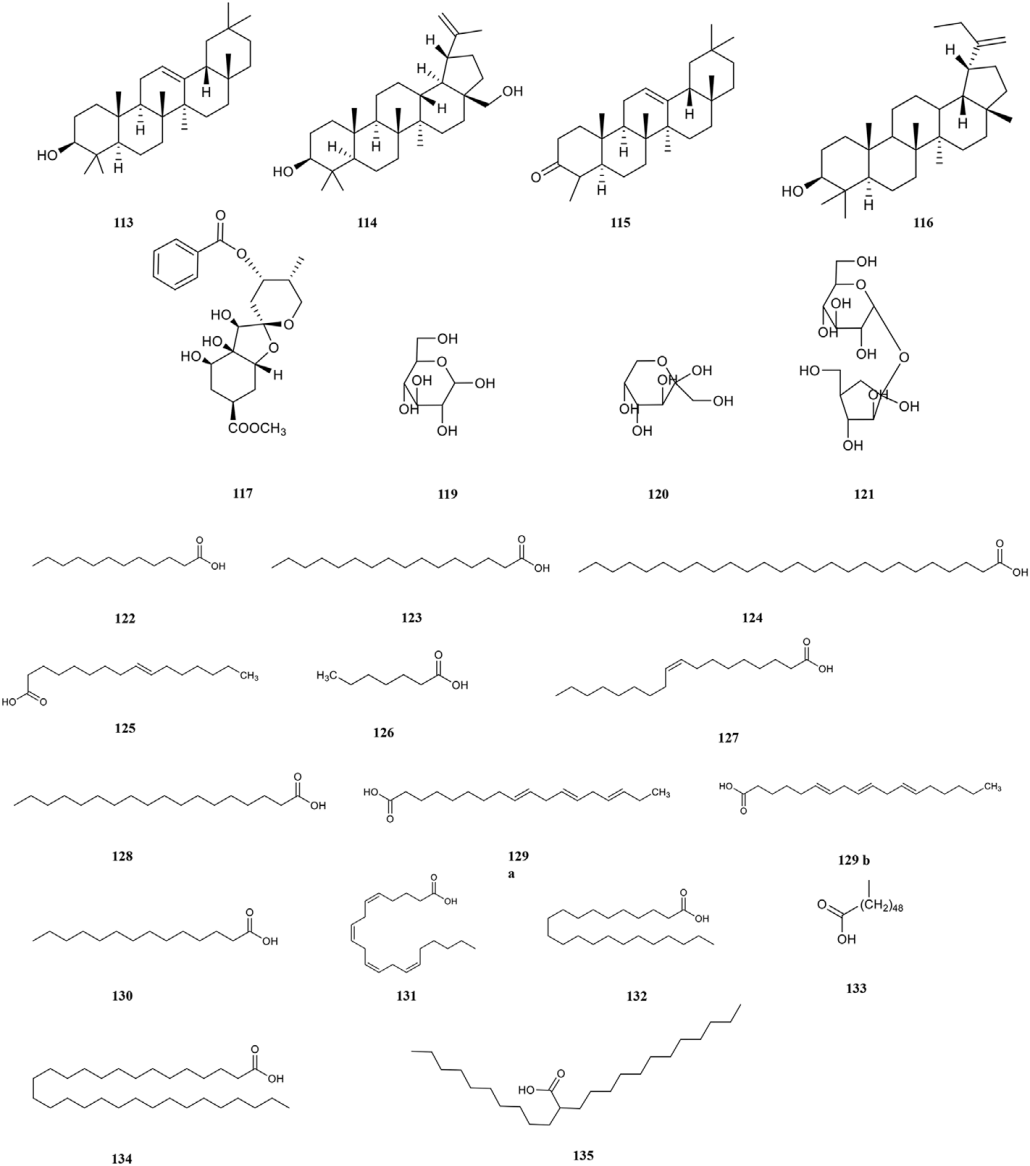
Chemical structures of terpenoids, polysaccharides, and fatty acids (113–135).

Terpenoids belong to the olefins, whose molecular formula is usually an integral multiple of isoprene. The biological activities of terpenoids are determined by variations in structure. For example, its anti-inflammatory, antitumour and antiviral activities are affected by the substituents at C-4, C-8, and C-10 positions and the parent nucleus. At present, terpenoids have become an important source for studying natural products and developing new drugs. PF contains a variety of terpenoids with strong antiviral and antitumour activities (Figure 5). β-amyrin (113), betulin (114), and β-amyrone (115) are the most fully researched terpenoids to date (Zhou et al., 2018., Xu et al., 2009). A new sesquiterpene namely phyllaemblinol (116) from PF ethanol extracts was separated by high performance liquid chromatography (HPLC) in 2016 (Zhang et al., 2016).

### 4.5 Polysaccharides

Polysaccharides play a positive role in immune regulation and cell recognition (Du et al., 2017). It has a variety of biological activities such as anti-tumour, anti-viral and hypoglycaemic. However, the biological activity of polysaccharides is influenced by many factors including the composition of monosaccharides, various isomers, sequence of glycogen linkage, and position and content of substituents (Bai, 2019). The content of polysaccharides in dry powder is 47.76 ± 0.37 mg/g, which accounts for 5% of the dry powder of PF, and that of fresh fruit pulp is 2.67 ± 0.02 mg/g, which accounts for 4.38% of the fresh fruit of PF (Liu B.k. et al., 2015). PF is rich in polysaccharides thereby characterizing it with a unique flavour, which is initially sour and astringent and then sweet. There are four main types of polysaccharides in PF (Figure 5), including pectin (118), glucose (119), fructose (120), and sucrose (121).

### 4.6 Fatty acids

PF includes a variety of unsaturated (80.36%) and saturated fatty acids (19.64%) (Figure 5) (Zhang et al., 2013). The saturated fatty acids in PF mainly include lauric (122), palmitic (123), hexacosanoic (124), melissic (134) (Kumaran and Karunakaran, 2006; Zhao et al., 2007; Xu, 2009), and lignoceric acid (135) (Li et al., 2015), *etc.* Palmitoleic (125), oleic (127), linolenic (129), are unsaturated fatty acids in PF (Zhao et al., 2007). Among them, the proportion of α-linolenic acid belonging to ω-3 polyunsaturated fatty acids is more than 50%, which is beneficial to the brain and neural development, cardiovascular health, and tumour suppression. Importantly, α-linolenic acid is an essential fatty acid that cannot be synthesised by the human body (Wang et al., 2007).

### 4.7 Amino acids

**FIGURE 6:**
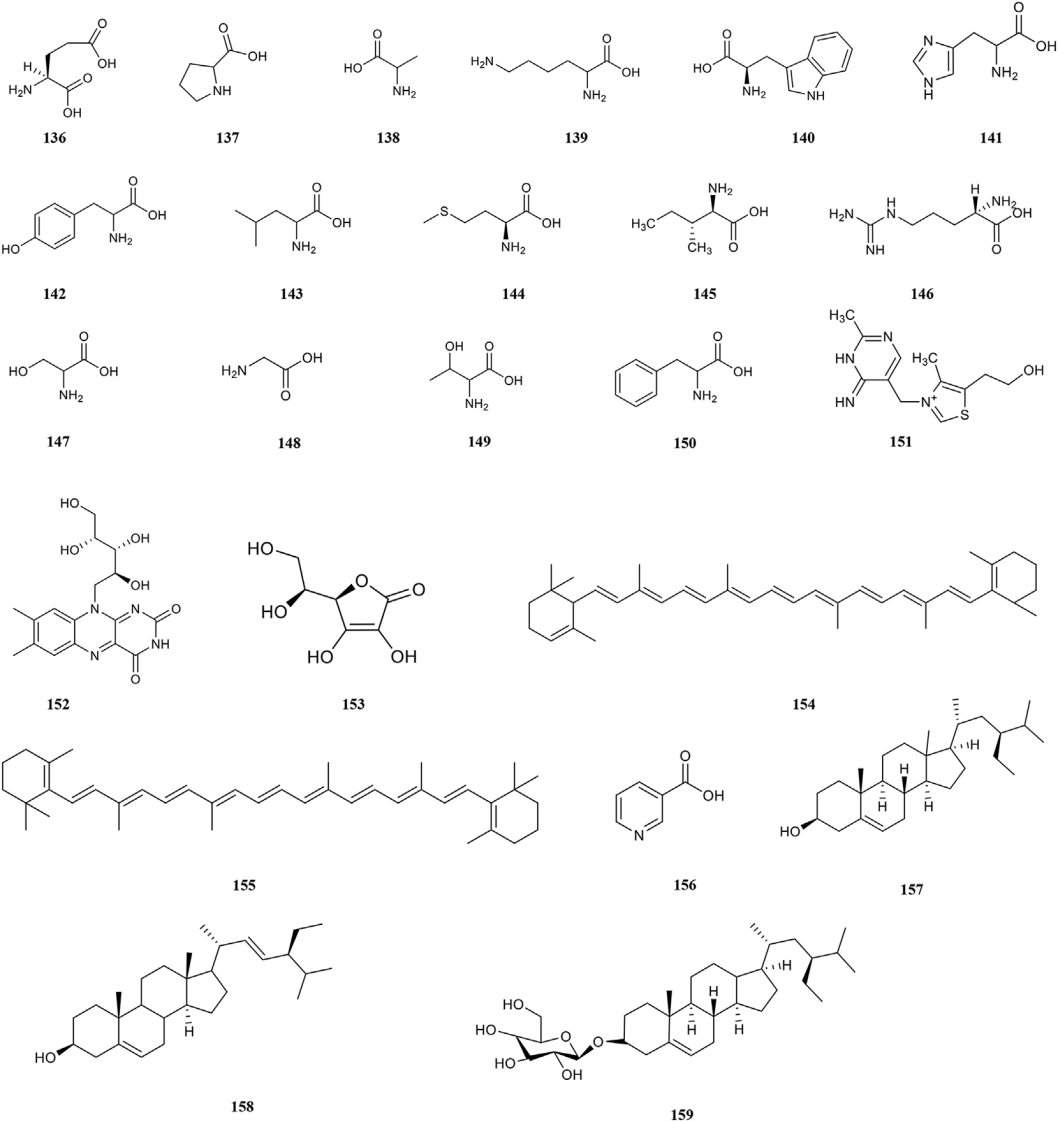
Chemical structures of amino acids, and other compounds (136–159).

Amino acids are involved in maintaining the body’s normal physiological, biochemical, and immune functions, as well as growth, development, metabolism, and other life activities. The antioxidant properties of amino acids are due to the ability to directly provide hydrogen atoms to bind to free radicals (Chen, 2019). PF contains 18 kinds of amino acids (Figure 6), among which 8 essential amino acids, such as glutamic acid (136), proline (137), alanine (138) and lysine (139), are required by the human body. And the amount of total amino acid is 1.851 mg/g. (Xia et al., 1997). Other amino acids include tryptophan (140), histidine (141), tyrosine (142), leucine (143), methionine (144), isoleucine (145), arginine (146), serine (147), glycine (148), threonine (149), and phenylalanine (150) (Wu et al., 2003).

### 4.8 Others

PF contains 18 kinds of trace elements such as Zn, Ge, Se, Gr, Fe, Cu, and Mn, and the major elements include K, Na, Ca and P (Hou, 2002; Chen et al., 2003). It is worth mentioning that the amount of Se in PF is up to 0.24–0.73 mg/100g, which is a Se-rich fruit with an extremely high health value (Jose et al., 1995). Moreover, about 12 vitamins such as vitamin B_1_ (151), vitamin B_2_ (152), ascorbic acid (153), and carotene (154, 155) are isolated from PF (Chen et al., 2003). Moreover, PF also contains alkaloid compounds, superoxide dismutase (SOD), steroids, protein, and other compounds (Kumaran and Karunakaran, 2006).

## 5 Pharmacological effects of *Phyllanthi Fructus*

**FIGURE 7:**
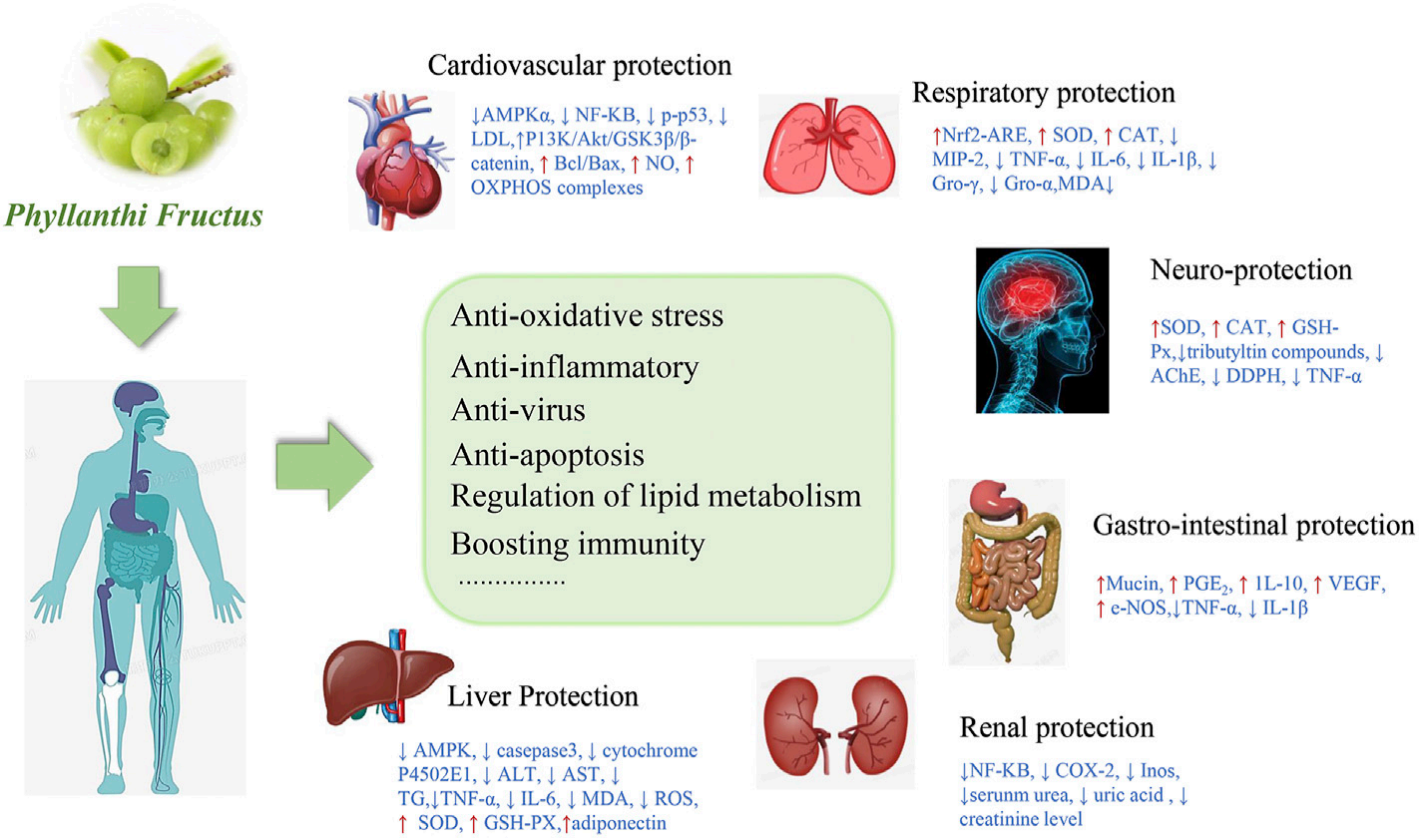
The pharmacological effects of PF on the body.

Many *in vitro*, *in vivo* and clinical studies have confirmed the pharmacological properties and functional activities of PF. The protective effect of PF on the body based on its biological activities were summarized in Figure 7.

### 5.1 Ethnopharmacology

PF has been utilized in Ayurveda, Siddha, and Unani systems in India, Sri Lanka, and China to treat various diseases. In the Chinese Pharmacopoeia (version 2020), PF is described as being cool in property, and sweet, sour, and astringent in flavour. It affects the stomach and lung meridians and acts to clear heat and cool blood, improve throat conditions, remove phlegm and promote good digestion. Therefore, PF can be applied in the treatment of blood heat and blood stasis, indigestion and abdominal distension. The recommended dosage is 3–9 g. Because PF is cool in property, and sour and astringent in flavour, individuals with deficiency-cold of the spleen and stomach, and pregnant women should use it with caution. In China, different ethnic groups use PF in slightly different ways. For example, Tibetan people mainly use the fruit to treat “*Blood disease*”, “*Chiba disease*” and “*Bacon disease*”, which are equivalent to the syndrome of blood stasis, liver and gallbladder disease, hypertension and digestive system disease (Luo, 2004), Dai people directly eat fresh fruits for curing tonsillitis, Uygur people use PF to treat high blood pressure, Zhuang people use dried fruit to treat cold, fever, cough, pharyngitis, enteritis and abdominal pain, Miao and Buyi people like to use wine or saltwater to soak PF to invigorate the stomach and relieve cough. Bai (Luo et al., 2000), Bulang, Jinuo and Lahu nationalities treat sore throat with fruit chewing, and Naxi people believed that PF could prevent influenza (Higby and King, 2013).

PF is considered one of the most important medicines in Ayurveda, which is called immortal medicine in Indian mythology. According to statistics, the frequency of use of PF in Ayurveda’s commonly used pharmaceutical system is up to 195 times. It is often used in the treatment of various heat syndromes and cancer (Thakur et al., 1993). PF is used in the traditional Turkish medicine system to treat diarrhoea, dysentery and gastroenteritis (Ernst, 1999). In North America, it is mainly used as a diaphoretic prescription and cathartic. In general, there is a surprising consistency of PF in the traditional medicine of different nationalities in China, India and Southeast Asia, as a tonic medicine for longevity (Higby and King, 2013).

As a traditional medicine, PF is widely used in modern medical practice with compound prescriptions combined with other botanical drugs (Table 5), and some of them are even used directly in their traditional form. PF as an aphrodisiac helps to increase sperm count (Udupa, 1985). PF is also used as a hair tonic in traditional formulations for enriching hair growth and pigmentation due to the presence of fixed oil (Thakur et al., 1993). They are applied as skin lighteners loved by women (Chaudhuri, 2004). Herbal formulations of burnt seeds and oils of PF have been used to treat skin afflictions (Dastur, 1952). Furthermore, PF helps to rejuvenate all body organ systems, provides strength and wellness, and boosts the immune system. The traditional efficacy of PF is an irreplaceable compass for researchers developing modern applications. Due to widely traditional usage, PF has also been developed in modern applications as drugs, health care products, and skincare products.

### 5.2 Biological activities

#### 5.2.1 Anti-oxidant activity

Before the 1990s, it was believed that PF contained a high level of ascorbic acid which is mainly responsible for antioxidant potency (Kapoor, 1989). A study later contradicted this claim, stating that PF did not contain any ascorbic acid at all and hydrolyzable tannins like emblicanin A and emblicanin B were responsible for its antioxidant function (Ghosal et al., 1996). However, a study in 2006 suggested that high levels of ascorbic acid may still be the main reason for the antioxidant activity of PF (Scartezzini et al., 2006). Assays including iron (III) reductive activity, iron (II) chelating activity, 2,2 diphenyl-1-picrylhydrazyl (DPPH)free radical scavenging, superoxide anion free radical scavenging, and hydroxyl free radical scavenging were conducted to evaluate the antioxidative properties of PF collected in four areas. The results showed that four PF samples exhibited varying degrees of antioxidant potential, regardless of different contents of ellagic acid, corilagin, gallic acid and ascorbic acid. Interestingly, the PF samples still had consistently potential in the antioxidant screens although the ascorbic acid level is not the highest (Poltanov et al., 2010). Bhattacharya et al. investigated the effect of PF on rat brain frontal cortical and striatal oxidative free radical scavenging enzyme levels and reported that the antioxidant activity of PF could be due to tannins including emblicanin A, emblicanin B, pedunculagin, and punigluconin, showing ascorbic acid like properties (Bhattacharya A. et al., 2000). Overall, it is not difficult to conclude that the strong antioxidant activity of PF is owed to the influence of various phytochemicals, such as ascorbic acid, hydrolyzed tannins, and phenolic acids, *etc.*

The antioxidative activity of PF mainly manifests as a scavenging effect on free radicals. Oxygen-derived free radicals, especially reactive oxygen species (ROS) including hydrogen peroxide, superoxide and hydroxyl compounds, are strongly inhibited by the polyphenols, flavonoids and polysaccharides in PF extracts (Shi and Yang, 1994; Li et al., 2010). ROS has been reported to be associated with diseases such as diabetes, Alzheimer’s disease, coronary heart disease, nephritis, cancer, arteriosclerosis, and diseases related to ageing (Chen and Kan, 2018). Related scholars evaluated the free radical scavenging ability by *in vitro* anti-oxidant tests, which showed that PF extracts have strong metal chelating, reducibility, 2,2′-azinobis-(3-ethylbenzothiazoline-6-sulfonic acid (ABTS) scavenging and DPPH scavenging ability (Wei et al., 2011; Wang et al., 2019). PF also has a scavenging effect on hydroxyl radicals and superoxide radical O_2_ *via* Fenton Reaction (Zhang et al., 2011). Malondialdehyde (MDA) is the main product of lipid peroxidation in the free radical membrane, whereas superoxide dismutase (SOD) is an important antioxidant enzyme that defends ROS (Liu et al., 2019). Therefore, increasing SOD activity and decreasing MDA content can reduce oxidative stress. *In vitro* tests have confirmed that PF ethanolic extracts protected RAW264.7 cells from oxidative damage by increasing glutathione content and total SOD activity and suppressing MDA content (Li et al., 2020). The active extracts of PF have been shown to possess antioxidant properties in several experimental models, which are shown in Table 2. In addition, another study reported the antioxidant activity of PF kernels methanolic extracts using the DPPH assay (IC_50_ 15 μg/ml) and H_2_O_2_ scavenging assay (IC_50_ 32 μg/ml) (Gupta et al., 2012). While the antioxidant activity of PF was explained by several studies using different solvent extracts, there are only a few *in vitro* studies on the antioxidant activity of the kernels and seeds coat. Thus, further research work should focus on the antioxidant activity of PF kernels and seeds’ coat waste.

**TABLE 2:** Anti-oxidant active components of PF extracts.

Extracts	Active compounds/monomer	Models and dosage range	Positive control	Antioxidant performances	References
Aqueous	Tannins^/_^ Flavonoids/^_^	RAW 264.7 cells stimulated with LPS (5 μg/ml) for 6 h. 0.125–2 mg/ml	Ascorbic acid (100 µM)	Dose-dependently decrease of DPPH, ROS, iNOS and COX-2 production	Wang et al. (2019)
Ethanol	Tannins/gorilagin Phenolic acids/gallic acid and ellagic acid	RAW264.7 cells stimulated with H_2_O_2_ (100–2,400 μmoL/L) for 24 h. 0.16–2.56 mg/ml	l-Ascorbic acid (0.16–2.56 mg/ml)	Increase of glutathione content and total superoxide dismutase activity, and decrease of MDA content	Li et al. (2020)
Aqueous/ethyl acetate	Flavonoids/quinic acid, gallic acid, and quercetin	Red blood cells (physiological) pretreated with glucose (5 or 50 mM) for 24 h. 50-200 ug/mL	_	Improvement of the plasma MDA, protein carbonylation, total protein, and albumin levels	Packirisamy et al. (2018)
Ethanol	Flavonoids/-	*C. elegans* stimulated with H_2_O_2_ solution (20 mmol/L). 1–4 mg/ml	_	Enhancement of thermal resistance, exercise capacity and lifespan	Wang et al. (2018)
Ethanol	Phenolics/gallic acid, chebulic acid and ellagic acid	*C. elegans* stimulated with H_2_O_2_ sol. 0–1.2 mg/ml	Ascorbic acid (0–0.15 mg/ml)	Enhancement of thermal resistance, extension, of lifespan and inhibition of AChE and BuChE activity, increase of SOD and CAT and decrease of MDA.	Wu et al. (2022)
Ethanol	Phenolics/gallic acid and ellagic acid. Tannins/isocorilagin, chebulanin and chebulagic acid	Mice were exposed to arsenic (sodium arsenite 3 mg/kg p.o.) for 28 days. 500 mg/kg p.o	_	Decrease of the lipid peroxidation, ROS production, the activity of caspase-3; increase of SOD,GSH, and CAT.	Singh et al. (2013)
Aqueous	Phenolics/gallic acid Flavonoids/-	Male Sprague Dawley rats induced by iodinated contrast agents (1,600 mg iodine/kg). 125 or 250 or 500 mg/kg/day for 5 days	Ascorbic acid and gallic acid (6.25, 12.5, 25, 50 and 100 μg/ml). Quercetin (25, 50, 100, 200 and 400 μg/ml)	Significantly decrease of MDA, and increase of total antioxidant capacity, SOD and CAT.	Tasanarong et al. (2014)
Methanol	Phenolics/-	Sprague Dawley rats induced by indomethacin (20 mg/kg p.o.) for 2 days. 100 mg/kg body-weight for 10 days	_	PF (100 mg/kg) significantly decreased the level of MDA and SOD.	Bandyopadhyay et al. (2000)
Aqueous	Tannins/-	0.39–12.5 μg/ml, The DPPH radical scavenging and reactive oxygen species inhibition assay	Ascorbic acid (100 µM)	A free radical scavenging activity (IC50 = 2.37 μg/ml), and protection of cells from ROS (IC501.77 μg/ml)	Majeed et al. (2019)

The anti-aging effects of PF is also attribute to its strong antioxidant ability. PF polyphenols showed a strong protective effect against the aging process in the *Caenorhabditis elegans* model through increasing thermal resistance, extending lifespan by 18.53%, reducing the activity of enzymes acetylcholinesterase (AchE) by 34.71% and butyrylcholinesterase by 45.38% (Wu et al., 2022). The life span of *drosophila* can be extended by ingesting certain concentrations of PF powder, nutrient solutions and juices (Zhao et al., 2018). Moreover, the extraordinary effect of PF in reducing the toxicity of heavy metals is also attributed to the antioxidant effects, such as its inhibitive effect on Ar-induced oxidative damage and apoptosis of mouse splenocytes, thymocytes and liver cells (Singh et al., 2013; Singh et al., 2014). The remarkable antioxidant and anti-aging potential of PF could be implemented in the food and pharmaceutical industry.

Although PF has proven its free radical scavenging activity *in vivo* and *in vitro*, it is difficult to achieve the desired outcome in the human body because of its low bioavailability like other herbal formulations. Researchers are developing various nano-formulations of PF extracts to overcome such bottlenecks. Rosarin et al. synthesize silver nanoparticles (AgNPs) loading with the aqueous extracts of PF, and it is reported that the IC_50_ value of PF (alone) and AgNPs is 30ug/mL and 20ug/mL, respectively (Rosarin et al., 2013). However, studies on the use of these nano-formulations in clinical applications are very limited so far. Thus the use of nanotechnology to enhance the therapeutic value of PF may be one of the future research prospects for scientists and clinicians.

#### 5.2.2 Analgesic, antipyretic, and anti-inflammatory activities

Phenolics, flavonoids and ascorbic acid in PF interfere with multiple processes in the pathogenesis of inflammation, such as the production of pro-inflammatory mediators, the expression of adhesion molecules, the adhesion of circulating leukocytes to the endothelium, and NF-κB activation (Lampronti et al., 2008; Saito et al., 2008; Muthuraman et al., 2011). *In vitro* studies showed that PF alleviated lipopolysaccharide (LPS)-induced inflammation in RAW 264.7 cells by decreasing the release of pro-inflammatory mediators, the expression of inducible nitric oxide synthase, cyclooxygenase-2 (COX-2) and NF-κB (Shi and Yang, 1994; Li et al., 2020). Another *in vitro* study found that PF prevented the further development of the inflammatory response in PAO1-induced IB3-1 CF bronchial epithelial cells by inhibiting the expression of the PAO1-dependent neutrophil chemokines interleukin-8 (IL-8), growth-regulating oncogene alpha (GRO-α) and GRO-γ, the adhesion molecule intercellular adhesion molecule (ICAM) 1 and the pro-inflammatory cytokine interleukin-6 (IL-6) (Nicolis et al., 2008). Furthermore, PF has a therapeutic effect on a variety of inflammatory diseases. PF aqueous extracts have a significant protective effect on the cartilage in patients with osteoarthritis by inhibiting hyaluronidase and type II collagenase activities (Sumantran et al., 2011). PF extracts inhibited the inflammatory response to benzopyrene B(a)P-induced acute lung injury by significantly reducing the levels of tumour necrosis factor-α (INF-α), IL-6, and macrophage inflammatory protein 2 (MIP-2) in B(a)P-induced lung cancer mice (Wang et al., 2017). PF extracts also have a good therapeutic effect on acute necrotizing pancreatitis. It can reduce serum lipase and IL-10 levels, and significantly increase the nucleic acid content, DNA synthesis rate, pancreatic proteins and pancreatic amylase contents in rats with sexual necrotizing pancreatitis (Sidhu et al., 2011).

Modern pharmacological studies have confirmed that the ethyl acetate, petroleum ether and n-butanol components in the methanol extracts of PF are potent anti-inflammatory and analgesic agents. These anti-inflammatory fractions treat gouty arthritis by inhibiting the release of the inflammatory factors including prostaglandin E2 (PGE2) and TNF-α, which may relieve the symptoms of redness, fever and severe pain that occur during gout attacks (Zeng and Cen, 2012). Dong Wook Lim et al. established a postoperative pain model (PI) produced by plantar incisions and a neuropathic pain nerve injury (SNI) rat model. Results found that PF extracts reduced the number of ultrasonic vocalizations in response to PI-related post-operative pain and hypersensitivity in response to von Frey stimulation of the hind paw in the PI model rats, as evidenced by an increased mechanical withdrawal threshold in the SNI-related neuropathic pain model rats. And PF extracts also significantly decreased pain-related pro-inflammatory cytokine levels in the dorsal root ganglion caused by neuropathic pain in SNI rats (Lim et al., 2016). The inhibitory effect of PF on the synthesis and/or release of pain and inflammatorymediators is similar to that of non-steroidal anti-inflammatory drugs (NSAIDs) (Golechha et al., 2014). Although the anti-inflammatory and analgesic effects of PF have been well studied, its inflammatory response in allergic reactions and immune-related diseases have not been carefully investigated. Besides, more research is needed to develop greener, safer, and more effective nano-anti-inflammatory drugs using PF as a raw material.

#### 5.2.3 Antimicrobial activity

Many *in vivo* and *in vitro* experimental studies have confirmed the powerful antibacterial activity of PF. Alkaloid components of the PF alcoholic extracts have significant antibacterial activity against Gram-positive (G^+^) bacteria, such as *Bacillus cereus*, *Bacillus subtilis*, *Staphylococcus aureus*, *Salmonella typhi*, *Salmonella paratyphi*, *Pseudomonas aeruginosa*, *Shigella boydii*, and some fungi, *Candida albicans*, *Sacharomyces cerevisiae*, and *Aspergillus niger*, were also included (Rahman et al., 2009). Tannoids, saponins, flavonoids, and terpenoids from the aqueous extracts of PF also exhibited potent antimicrobial activity against *Enterobacter cloacae*, *Escherichia coli*, and *Klebsiella pneumonia* (Saeed and Tariq, 2007; Kumar A. et al., 2011). The chloroform soluble fraction of the PF methanolic extracts exhibited an inhibitory effect against some G^+^ and Gram-negative (G^−^) pathogenic bacteria and strong cytotoxicity with an IC_50_ of 10.257 ± 0.770 μg/ml (Rahman et al., 2009). MgO nanoparticles and silver nano-particles loading with the extracts of PF which were synthesized through green synthesis showed potential anti-microbial activity against various pathogenic bacteria such as *Staphylococcus aureus*, *Pseudomonas aeruginosa*, *Escherichia coli* and *Bacillus subtilis* (Ramanujam and Sundrarajan, 2014; Ramesh et al., 2015). Due to the effects against resistant strains, nano-formulations synthesized using PF has the potential to be developed as the carriers for antibiotics. In traditional medicine, Basant (a mixture of herbs containing PF extracts, curcumin, soapberry saponin, aloe vera and rose water) inhibits the growth of strains and clinical isolates of *Neisseria gonorrhoeae*, among which are resistant to penicillin, tetracycline, nalidixic acid or ciprofloxacin (Dong et al., 2014). Moreover, PF essential oil extracted by Zhao et al. using the supercritical CO_2_ extraction technique showed good inhibition of common food contaminating bacteria (*Bacillus subtili*s (G^+^), *Staphylococcus aureus* (G^+^), *Salmonella* (G^−^)), and the inhibition effect on G^+^ bacteria was greater than that on G^−^ bacteria (Zhao et al., 2007). Together, these results suggest that PF can be used as a plant-derived antimicrobial agent in the clinical control of drug-resistant pathogens and has the potential to control food-contaminating bacteria. However, the specific antibacterial components and mechanism of PF are not yet clear and need in-depth study. Similarly, there are few studies on the antifungal properties of PF, so further studies are required to find out the antifungal active ingredient.

#### 5.2.4 Anti-viral activity

Previous investigations revealed that PF has inhibitory effects against human immunodeficiency virus (HIV), H_1_N_1_ influenza virus, herpes simplex virus (HSV), human papillomavirus (HPV), hepatitis B Virus (HBV), and severe acute respiratory syndrome coronavirus 2 (SARS-CoV-2). PF methanol extracts and water extracts have a good inhibitory effect on HIV reverse transcriptase and H_1_N_1_ infection (Sun et al., 2002; Kong et al., 2016). 1,2,4,6-tetra-*O*-galloyl-*β*-d-glucose from PF has anti-HSV activity *in vitro* (Xiang et al., 2011). The anti-HSV-1 and anti-HSV-2 effects of polyphenols isolated from PF extracts were confirmed in a guinea pig cutaneous HSV-1 infection model, cytopathic effect observation method, and thrombocytopenia assay. And the mechanism may be direct inactivation of the virus and inhibition of early viral DNA replication, thereby inhibiting HSV virus-induced cytopathy (Qu et al., 2010). PF also has a good inhibitory effect on HPV-16 and HPV-18 viruses by dose-and time-dependent reduction of DNA binding activity of constitutively active activator protein-1 in HeLa cervical cancer cells (Mahata et al., 2013). Elaeocarpusin and digallic acid in PF ethanolic extracts showed remarkable anti-HBV activity. These compounds screened by HBV-DNA transfected HepG2 2.1.5 cell model showed strong inhibition for the secretion of HBeAg and HSeAg with IC_50_ of 10.2, 79.0 μg/ml and 5.5, 101.0 μg/ml, respectively (Hou, 2006). Moreover, numerous computational screening studies have mapped out the potential bioactive compounds from medicinal plants against the targets of severe acute respiratory syndrome coronavirus 2 (SARS-CoV-2) (Siddiqui et al., 2020; Natesh et al., 2021). PF extracts contain bioactive compounds such as flavonoids, phenols, tannins, and alkaloids with good antiviral activity against SARS-CoV-2, which may guide the development of novel anti-neoplastic pneumonia prophylactic drugs (Krupanidhi et al., 2020; Kumar et al., 2020). Murugesan’s study also found that PF quercetin has a good binding effect on SARS-CoV-2 main protease and could be developed as a promising inhibitor of SARS-CoV-2 main protease (Murugesan et al., 2021). However, the technical difficulties in synthesizing complex natural compounds with good antiviral activity from PF need to be solved and the antiviral activity of PF needs to be validated in more animal and clinical studies.

#### 5.2.5 Anti-tumour activity

Nowadays, plant-originated natural products constitute a considerable proportion of commercial anticancer drugs (Shynu et al., 2011). The anticancer properties of some molecules identified by high performance liquid chromatography (HPLC) from PF extracts have been repeatedly demonstrated (Table 3). Corilagin has significant growth inhibition on human nasopharyngeal carcinoma cells kB, ovarian carcinoma cells A2780, osteosarcoma cells OS-732, human lung adenocarcinoma cells A549, mouse liver cancer H22 tumour, murine *Lewis* tumour and human HCT-8 (Liu et al., 2002). Geraniin can induce the apoptosis of human melanoma cell A2058 and inhibit the proliferation of human lung adenocarcinoma cell A549 (Lee et al., 2008; Li et al., 2013). Chebulinic acid has obvious anti-leukemia, anti-cervical cancer and anti-colon cancer HT-29 cell effects and can inhibit the growth of malignant tumour (Yi et al., 2004). Chebulagic acid also has a good inhibitory effect on the growth of eye cancer cells and HSC-T6 hepatocytes by inhibiting vascular endothelial growth factors (Athira et al., 2013; Chuang et al., 2011; Kumar, N. et al., 2014). These results suggested that tannins might be responsible for the growth inhibition of cancer cells. However, animal and clinical trials of PF active monomers as complementary drugs for cancer treatment and prevention are lacking. Interestingly, crude PF extracts are also capable of tumour inhibition. PF extracts enriched with polyphenols or aqueous extracts have shown cytotoxic activity against cervical and ovarian cancer cell (De et al., 2013; Zhu et al., 2013). In a murine model of skin carcinogenesis, continuous administration of PF extracts at 100 mg/kg reduced tumour incidence by ∼60% (Sancheti et al., 2005). The pre-clinical experiments of various cancer cell lines (A549 [lung], HepG2 [liver], HeLa [cervix], MDA-MB-231 [breast], SK-OV-3 [ovary] and SW620 [colorectal]) showed that the water extracts of PF (50–100 μg/ml) can induce apoptosis by activating caspase-8 and caspase-3/7 and upregulating Fas expression (Ngamkitidechakul et al., 2010). PF extracts also sensitized refractory high-grade serous ovarian cancer (HGSOC) cells with high drug resistance by regulating key signalling molecules of angiogenesis and metastasis (De et al., 2020).

**TABLE 3:** The anti-tumour mechanisms of active components in PF extracts.

Extracts	Active compounds/monomer	Anti-tumour mechanisms	Anti-tumour indicators	References
**Induction of tumour cell apoptosis**				
Ethanol	Phenolics/gallic acid	1. Down-regulation of ERK/p-ERK signalling pathway	1.↓ Proliferation and tubule formation of human cervical cancer cells HeLa and HTB-35	Zhao and Hu., 2013
		2. The activation level of ERK1/2 was down-regulated	2.↓ The proliferation of human osteosarcoma cell lines U-2OS and MNNG/HOS	Liang et al. (2012)
Methanol	Tannins/1,2,3,4,6-penta-*O*-galloyl-*β*-glucose	3. Activation of the MAPK8/9/10 and JNK signalling pathways	3.↑ Autophagy occurred in HepG2, MCF-7 and A549 tumour cells	Dong et al. (2014)
Ethanol	Flavonoids/progallin A Tannins/_	4. G_1_/M and G_2_/M cell cycle arrest was induced	4. ↓ The proliferation of human liver cancer cell BEL-7404	Zhong et al. (2011)
**Inhibition of tumour metastasis**				
Methanol	Phenolics/penta-1,2,3,4,6-*O*-galloyl-*β*-d-glucose and geraniin	1.The expression of key enzymes MMP-2 and MMP-9 in tumour cells were inhibited	1.↓ Proliferation, migration, invasion and adhesion of human fibrosarcoma HT1080 cells	Sancheti et al. (2005)
		2. Inhibition of Akt and ERK1/2 activities	2.↓Proliferation and migration of rat glioma cell C6 and human glioma cell U373	Li et al. (2020)
Ethanol	Phenolics/gallic acid	3.Inhibition of tumour angiogenesis	3.↓ Angiogenesis and growth of new blood vessels (Human Placental Vein Angiogenesis Model)	Ji, (2010)
**Regulation of body immunity**				
Ethanol	Ascorbic acid	1.NK cell activity and antibody-dependent cell-mediated cytotoxicity were significantly enhanced	1.↑ The lifespan of immunodeficient mice vaccinated with Dalton’s lymphoma ascites	Suresh and Vasudevan., 1994
	Tannins/corilagin	2. The expression of the E3 ubiquitin ligase RING finger protein 8 were attenuated	2. ↓Proliferation and migration of esophageal squamous cell carcinoma	Qiu et al. (2019)
**Regulation of the related gene expression**				
Aqueous	Tannins/corilagin	1. The conduction of the TGFβ/AKT/ERK/Smad signalling pathway was targeted to inhibit	1.↓ The growth of ovarian cancer cells	Liu et al. (2002)

How do these extracts exert anti-tumour effects? There are several possibilities. First of all, PF can prevent DNA damage and tumour occurrence caused by ROS (Majeed et al., 2009; Hazra et al., 2010). However, it is unclear from the current findings to what extent ROS contribute to the underlying pathology. Secondly, many of the anticancer properties of PF extracts may be brought about through inhibition of this transcription factor binding with its cognate DNA binding elements (Hoesel and Schmid, 2013). Thirdly, PF extracts have anti-inflammatory activity that may prevent related cancers caused by inflammation (Golechha et al., 2014). Fourth, the antitumour activity of PF may be due to the blocking cell cycle in the G2/M phase (Huang et al., 2017). Either crude PF extracts or purified components show effective cytotoxicity to most cancer cell lines, but its resistance mechanism still exists. Remarkable results in a study were not reported when PF extracts were examined for liver tumours induced by initiation with diethylnitrosamine followed by promotion with 2- acetylaminofluorene (Sultana et al., 2008). Although a large number of studies have reported the beneficial effects of PF for the treatment of cancer, these results are still in the cell and animal experimental stage. The anti-tumour effects of PF are yet to be explored in more clinical trials to develop effective new anti-cancer drugs.

#### 5.2.6 Immunomodulatory and anti-fatigue activities

The activation of the immune system is an effective protection mechanism against internal and external threats. *In vitro* and *in vivo* studies have been performed on the extracts of PF and bioactive constituents to evaluate their immunomodulating effects. The tannins and polyphenols responsible for immunomodulatory properties of PF included gallic acid, ellagic acid, corilagin, chebulagic acid, and quercetin (Scartezzini and Speroni, 2000). PF has been demonstrated to regulate the immune system *via* various mechanisms. Chebulagic acid found in PF has been reported to inhibit the NF-κB signaling pathway. Impaired regulation of NF-κB may lead to various immune/non-immune related disorders including cancer, inflammatory, and autoimmune diseases (Hamada et al., 1997). Thus, the immunomodulatory effects of PF could be its potential to regulate the NF-κB signaling pathway. Diethyl ether extracts of PF potentially suppressed calcium ionophore A23187-induced LTB4 release from human neutrophils and TXB2 release in platelets (Vormisto et al., 1997). Ram et al. reported that Chromium (VI) induced cellular stress and immunosuppressive effects on lymphocyte proliferation were ameliorated following the treatment with PF and it also restored the altered levels of IL-2 and γ-IFN (Ram et al., 2002). Luteolin and apigenin are important flavones found in PF and were able to modulate the immune system by attenuating LPS-induced splenocyte proliferation and enhancing humoral immune responses (Cui et al., 2010; Wang et al., 2014). The investigation has also shown the ability of these compounds to enhance natural killer (NK) cells and cytotoxic T lymphocytes (CTL) activities. Thus, the immunomodulatory properties of PF extracts could be due to the synergistic effects of these cells. In TCM, PF in combination with *Radix Plantarum*, *Astragalus* spp., *Salvia miltiorrhiza*, *Panax notoginseng*, *Ginkgo biloba* and *Radix Puerariae* can improve immunity, reduce blood lipid and protect the heart and cerebrovascular integrity (Zhu et al., 2018). PF also has an excellent protective effect on immune organs while inhibiting tumour growth in mice *via* the research methods of TCM serum pharmacology (Luo, 2010). PF showed a wide range of application values for the treatment and prevention of various diseases because of the immunomodulatory activities.

PF has an anti-fatigue effect by increasing glycogen stores, reducing glycogen consumption and controlling fatty energy supply. An anti-fatigue evaluation model of the simulated plateau-exposed mice was established to observe the effect of PF. The research results showed that a low-dose extracts of PF (0.383 g/kg) can significantly prolong the exhausted swimming time of mice, promote the elimination of lactic acid after exercise and increase liver glycogen reserves. In the middle dose group (0.167 g/kg), only liver glycogen significantly increased, whereas in the high-dose group (11.67 g/kg), there was a significant increase in exhaustive swimming time (Zhang et al., 2011). These results indicate that PF extracts have broad application prospects for improving the working ability of the plateau.

#### 5.2.7 Hypoglycemic and hypolipidemic activities

There is increasing evidences that PF may have a positive role in controlling disorders of blood glucose and lipid metabolism. Polysaccharides in PF extracts can significantly improve insulin resistance or β-cell dysfunction, thus, having a therapeutic effect on type 2 diabetes (Dong et al., 2009). Polysaccharides also remove OH, O^2-^, and DPPH, which slows down the oxidative stress of patients with diabetes and effectively prevents diabetic complications (Wang et al., 2013; Guo et al., 2014). Favonoids from PF exhibited significant hypoglycemic activity. Its main mechanisms of action are to maintain β-cell activity, inhibit β-cell apoptosis and insulin-like effects, promote peripheral tissue regulation of glucose metabolism, maintain biofilm stability through antioxidation, protect pancreatic tissue and regulate the release and activity of enzymes related to glucose metabolism (Kang et al., 2011). Similarly, the hypolipidemic activity is due to the inhibition of hepatic 3-hydroxy-3-methylglutaryl coenzyme A reductase and the elevation of lecithin-cholesterol acyltransferase by flavonoids of PF (Anila and Vijayalakshmi, 2002). PF extracts have the same effect as the anti-diabetic drug chlorpropamide in reducing serum glucose and glycosylated haemoglobin (HbA1C) (Daisy et al., 2009). When PE was used in combination with hypoglycaemic drugs, it was significantly more effective in controlling blood glucose and lipid levels than hypoglycaemic drugs alone (Linda and Mary, 2014). A 21-day clinical study showed that PF reduced fasting and 2-h postprandial blood glucose levels and cholesterol and triglyceride (TG) levels in patients with diabetes (Akhtar et al., 2011). The alcohol-extracted part of PF could increase the biosynthesis of GLUT4 by enhancing the expression of GLUT4 mRNA in skeletal muscle and accelerating the transmembrane transfer of glucose and the utilization of glucose by peripheral tissues, thereby improving insulin resistance and treating diabetes (Kang et al., 2011). Moreover, some studies showed that a regular intake of PF powder can effectively reduce levels of total cholesterol, LDLs, TGs, and very-low-density lipoproteins (VLDLs) and increase levels of high-density lipoproteins (HDLs) in patients with type II hyperlipidemia by mechanisms similar to that of simvas-tatin (Gopa et al., 2012). A study by *Balusamy et al.* suggested that the digallic acid of PF showed significant anti-lipolytic activity compared to the standard drug orlistat. In the mature adipocytes, digallic acid significantly reduces TG accumulation by down-regulating adiponectin, peroxisome proliferator-activated receptor γ (PPARγ), CCAAT/enhancer-binding protein α, and fatty acid-binding protein 4 (FABP4), and triggers adipocyte apoptosis by up-regulating the expression of Bax/Bcl-2 (Balusamy et al., 2020). Hence, PF negatively regulates adipogenesis by initiating fat cell apoptosis and therefore it can be considered a potential herbal medicinal product for treating obesity.

Hyperglycaemia and hyperlipidemia interact with each other and exacerbate each other. Both endanger the health of the heart, brain, kidneys, and other organs. Many scholars have found through animal studies and clinical trials that the bioactive components from PF can prevent complications caused by diabetes and dyslipidemia through different mechanisms (Table 4). At present, most of the existing literature focuses on research into the therapeutic mechanisms of certain compositions or extracts of PF, which may show the synergistic effects of multiple compositions. Although PF contains hundreds of compounds, current reports focus on polysaccharides, ellagic acid, gallic acid, chebulic acid, *etc.* However, it does not mean that other compounds have no effects. Moreover, PF has preventive and therapeutic effects on diabetes and hyperlipidemia, but there is still a lack of sufficient evidence in as food and dietary supplement. We hope that PF and related products can provide a new healthy diet reference for high-risk groups and patients with diabetes in the future.

**TABLE 4:** The possible mechanisms underlying PF extracts against hyperlipidaemia and diabetic complications.

Complications	Extracts	Active compounds/monomer	Positive control	Mechanisms	Improvement indicators	References
**Cardiovascular diseases**	Aqueous	Phenolics/ellagic acid and gallic acid	Simvastatin capsule (20 mg p.o.)	1. Reduction of oxidative stress and ROS production in cardiomyocytes	**Animal experiment**: LDH ↓, creatinine kinase MB (CK-MB) ↓, SOD ↓, GSH ↓, serum creatinine (Scr) ↓, left ventricular wet LV/BW and HW/BW ratios ↑, blood pressure (BP) levels ↓	Gopa et al. (2012)
				2. Regulation of adipokines and improvement of lipid metabolism disorders		
				3. Reduction of the advanced glycation end products (AGEs) production		
	Aqueous	Phenolics/-Tannins/gallotannin	_	4. Regulation of cardiac autonomic dysfunction	**Clinical experiments**: TC ↓, LDL ↓, TG ↓, VLDL↓, HDL↑	Patel and Goyal, (2011)
				5. Improvement of glucose utilization and maintaining of glucose homeostasis by restore and regenerate β-cell structure		
**Nephropathy**	Methanol	Tannins/gallotannin, Phenolics/ellagic acid	Insulin (3 IU/kg s.c.).	1. Oxidative stress-induced apoptosis was reduced	**Animal experiment**: serum urea ↓, uric acid ↓, Scr ↓, total protein (TP) ↑, albumin (Alb) ↑, aldose reductase (AR) ↓, sorbitol dehydrogenase (ID)↓, HbA1c ↓, urinary glycosylated albumin ↓, renal carboxymethyl lysine ↓, pentoside ↓, sorbitol ↓, fructose level ↓, and IL-6↓, IL-1b ↓, TNF-α↓ and MCP-1 ↓ in kidney tissue	Chandak et al., 2009; Chao et al., 2010; Latha and Daisy, 2011
				2. The production of AGEs was reduced		
				3. Over-activation of poly-ADP-ribose polymerase and mRNA expression of aldose reductase in the kidney were inhibited		
				4. The release of renal inflammatory cytokines was inhibited		
				5. The activity of renal aldose reductase (AR) was inhibited		
**Neuropathy** (neuropathic pain)	Aqueous	Phenolics/gallic acid	Insulin (10 IU/kg, s c.).	1. Scavenging free radicals and anti-oxidation	**Animal experiment**: ↓ TNF-α, IL-1β and TGF-β1 levels in the serum and sciatic nerve of diabetic rats, regulation of oxidative-nitrosation stress in diabetic rats, ↓ neuropathic pain	Tiwari et al. (2011)
				2. Inhibition of hyperglycemia leading to reactive dicarbonyl formation of AGEs		
				3. Regulation of lipid metabolism and axon degeneration in the brain		
**Diabetic cataract**	Aqueous	Tannins/*β*-glucogallin Flavonoids/quercetin	Quercetin (50 µg/ml p.o.)	1. Aldose reductase activity and sorbitol formation were significantly inhibited	**Animal experiment**: ↓ eye’s lens high pressure, ↓ the accumulation of galactitol, sorbitol in red blood cells, lens and sciatic nerve	Suryanarayana et al. (2004)
	Methanol	Phenolics/ellagic acid	_	2. Polyol-induced oxidative stress pathways in the lens are altered	**Rat lens organ culture model**: normalization of the altered a-crystallin profile, and preservation of a-crystallin chaperone activity	Kumar et al. (2011b)
				3. Inhibition of hyperglycaemia-induced aggregation and insolubility of lens proteins		
**Protein wasting**	Methanol	Phenolics/ellagic acid, and gallic acid	Insulin (3 IU/kg s.c.)	1. Inhibition of oxidative stress caused by hyperglycemia	**Animal experiment:** ↑ total hemoglobin (HB), maintaining food and water intake, and maintaining body weight	D'Souza et al., 2014; Patel and Goyal, 2011; Wang, 2017
				2. Improvement of insulin insensitivity or insulin resistance to maintain glucose homeostasis		
				3. Inhabition of α-glucosidase		
**Hepatopathy**	Methanol	Flavonoids/epigallocatechin gallate Phenolics/ellagic acid, and gallic acid	Simvastatin (1.77 mg/kg p.o.)	1. Antioxidant activity in liver; anti-vascular oxidative stress	**Animal experiment:** ↓mRNA expression of IL-1β, IL-6, TNF-α, and monocyte chemoattractant protein-1 (MCP-1), ↓serum resistin levels, ↓plasma total cholesterol and triglyceride level, ↓ plasma ALT and AST levels, ↓hepatic triglyceride and cholesterol content	Yoshimura et al., 2013; Liu et al., 2015b; Ding et al., 2015
				2. Inhibition of Smad2 phosphate and Smad3 phosphate; improvement of energy expenditure by activating the AMPK pathway; improvement of mRNA expression levels of LDL-Rs		
				3. Anti-inflammatory; correction of abnormal hepatic metabolism		

#### 5.2.8 Neuroprotective activity

PF has been considered to exert therapeutic effects on neurological ailments due to its antioxidant, cholesterol-lowering and anti-inflammatory properties. The ethanol extracts of PF can increase the levels of SOD, CAT, and glutathione peroxidase (GSH-Px) in rat brain tissue homogenates and decrease the level of tributyltin compounds and the activity of AchE. Therefore, PF can improve the cognitive ability of rats (Uddin et al., 2016). Ellagic acid and gallic acid from PF methanol extract improved the performance of Alzheimer’s disease rodents in neuro-behavioural tests by inhibiting AchE activity (IC_50_ < 100 μg/ml) and scavenging ability of DPPH (IC_50_ < 10 μg/ml) (Mathew and Subramanian, 2014; Uddin et al., 2016). A previous study established a depression mouse model using a tail suspension test and forced swimming test, then this research observed that PF has significant anti-depressant activity, and its efficacy is comparable to imipramine and fluoxetine. In addition, a reduction in the monoamine oxidase-A enzyme in the mouse brain after using antagonists of the α_1_ adrenergic, serotonin, dopaminergic D_2_, and γ-aminobutyric acid-b receptors caused the anti-depressant activity of PF to be ineffective. Hence, PF might interact with these receptors to exhibit anti-depressant activity (Dhingra et al., 2012). Moreover, PF ethanol extracts have antiepileptic activity by reducing glucose and cortisol levels in the brain tissue of mice in a restraint stress model and improving the performance in the elevated plus-maze test (Kumar, C.P. et al., 2014). Kainic acid of PF attenuated global tonic seizures and persistent status epilepticus in pentylenetetrazole-induced epileptic rats, thereby improving the cognitive dysfunction, which may be related to the decrease of TNF-α levels in rat brain tissue (Golechha et al., 2010; Golechha et al., 2011). Furthermore, PF can resist the toxicity and cognitive impairment induced by aluminum chloride in rats by regulating oxidative stress and reducing the expression of apoptosis markers including Bax, caspases-3, caspases-9, cytosolic cytochrome c and p-Tau (Thenmozhi et al., 2016; Bharathi et al., 2019). PF also has a significant neuroprotective effect on oxidative DNA damage of neurons induced by H_2_O_2_ (Ramakrishna et al., 2014). The combined treatment with PF, *Arrowshaped Tinospora Root*, and *Ocimum tenuiflorum* also improved learning and memory disorders caused by scopolamine, diazepam and cyclosporin (Malve et al., 2014). In short, PF extracts have been hypothesized to act through multiple neuroprotective pathways. To date, most of the studies have shown neuroprotective effects of PF crude extracts. However, only a few research focuses on the molecular mechanisms by which the active monomers of PF act on neural cells. Further studies on the neuroprotective effects of PF extracts and monomers are crucial to uncovering the molecular mechanisms and gene regulation underlying these effects. Furthermore, given the neuroprotective properties exhibited by PF, more researches will focus on the clinical effects of PF on depression, epilepsy, and neurodegenerative disorders in future.

#### 5.2.9 Multiorgan functional protective activity

Over 40 years of scientific research on PF have confirmed the protective effect on many organs. Inflammation and oxidative stress play a major role in liver injury. And the phytoconstituents viz. ascorbic acid and gallic acid present in PF protect the hepatocellular environment against toxic moieties generated from oxidative stress or inflammation (Chen et al., 2011). PF extracts significantly increased hepatic SOD and GSH-Px activities, and inhibited MDA and ROS production to protect the liver by regulating protein kinase (AMPK) and acetyl coenzyme a carboxylase signaling pathways (Malar and Mettilda, 2009; Yang et al., 2016). PF also reduced liver inflammatory damage by decreasing the concentration of TNF-α and IL-6, down-regulating casepase3 gene expression and hepatocyte apoptosis levels (Zhang et al., 2017). There is a paucity of literature on the synergistic hepatoprotective mechanisms of multiple active ingredients in PF extracts. The mechanism of action needs to be further elucidated, which will provide a scientific basis for the development of hepatoprotective combination drugs. In TCM, PF along with the combination of other herbs is widely used to treat wheezing coughs, eruptions and other respiratory disorders (Sai et al., 2010). Flavonoids in PF extracts attenuate the development of lung inflammation in B(a)p or H_1_N_1_ influenza virus-infected mice by reducing the levels of pro-inflammatory cytokines MIP-2, TNF-α, IL-6, and IL-1β in lung tissue and the number of lymph nodes on the lung surface. (Kong et al., 2016; Wang et al., 2017). PF mixture improves the clinical efficacy and lung function of patients with acute exacerbation of chronic obstructive pulmonary disease (Chen and Jing, 2012).

PF showed in numerous animal studies to be cardioprotective and anticoagulant (McCord, 1985; Bhatia et al., 2011). The cardioprotective effect of PF is attributed to its high content of antioxidant active substances (Bhatia et al., 2011). For example, ascorbic acid reacts with singlet oxygen radicals and hydrogen radicals to produce semi-dehydroascorbic acid, which inhibits oxidative modification of LDL and improves atherosclerosis-induced endothelial damage (Villacorta et al., 2007). PF inhibited the accumulation of intracellular oxidants and the conversion of phosphorylated P53 protein (phospho-p53) in the nucleus by upregulating the PI3K/Akt/GSK3β/β-catenin cardioprotective signaling pathway and downstream cascade reactions, thereby reducing endothelial cell apoptosis induced by oxidative load and inhibiting cardiomyocyte necrosis and fibrosis (Liu et al., 2007a; Thirunavukkarasu et al., 2015). In TCM research, the Tibetan medicine prescription *Wuwei Yuganzi* plays a role in protecting the myocardium by regulating the expression of Bcl-2 and Bax, increasing the ratio of Bcl-2/Bax expression and the level of NO in the myocardium of IRI rats (Tan et al., 2015). *Amalaki Rasayana* (AR, an Ayurvedic formula made with PF and other ingredients) improved myocardial contractility and the energy efficiency of cardiomyocyte mitochondria in aged rats by upregulating the expression of OXPHOS complex and β1/2 adrenergic receptor gene, downregulating the phosphorylation level of AMPKα and the expression of NF-κB (Kumar et al., 2017).

Preclinical studies have shown the beneficial effect of PF in the treatment and/or prevention of various GI problems like ulcerative colitis and Crohn’s disease (Romano et al., 2014; Saxena et al., 2014). The ethanolic extracts of PF had a protective effect against NSAID-induced gastropathy by significantly increasing the levels of mucin and PGE2, decreasing the levels of pro-inflammatory cytokines (TNF-α, IL-1β) and up-regulating the levels of anti-inflammatory cytokines (IL-10) (Chatterjee et al., 2011). The gallic acid-rich fraction of PF extracts exerted therapeutic effects on indomethacin-induced gastric ulcer in mice by increasing the levels of pro-angiogenic factors like PGE2, vascular endothelial growth factor (VEGF), hepatocyte growth factor, von Willebrand Factor VIII and endothelial NOS (Chatterjee et al., 2012). In addition, the chloroform part and ethyl acetate part of PF extracts can increase gastrointestinal motility and act as a laxative by activating cholinergic receptors; therefore, it is used to treat indigestion and constipation (Mehmood et al., 2013). PF is reported to have diuretic potential. PF has a nephroprotective effect on producing nephrogenesis by significantly reducing arterial blood pressure, blood urea nitrogen, and creatinine levels and decreasing the expression of iNOS, COX-2, and NF-κB in aged rats (Yokozawa et al., 2007).

#### 5.2.10 Others

PF contains a high concentration of ascorbic acid, which can prevent and treat scurvy (Jain et al., 2015). PF extracts significantly antagonized the *Vipera russellii* and *Naja kaothia* venom-induced lethality *in vivo* and *in vitro* experiments (Alamand and Gomes, 2003). Furthermore, its combination with high levels of ascorbic acid and polyphenols can promote wound healing by regulating collagen synthesis, extracellular matrix protein synthesis and oxidative stress (Sumitra et al., 2009).

## 6 Toxicology of *Phyllanthi Fructus*

Since ancient times, PF has been used for the treatment of diseases. *In vivo* and *in vitro* studies have verified its role in the inhibition of various pathogenesis. The results of the safety evaluation showed that the acute oral maximum tolerable dose of PF was greater than 20.0 g/kg b. w., which was practically non-toxic according to acute toxicity classification (Wen et al., 2018). Acute toxicity experiment showed no behavioural changes or symptoms of toxicity in rats given up to 2000 mg/kg b. w. of PF ethanol extract orally for 14 days. (Uddin et al., 2016). A previous study has administrated rats with PF juice (up to 9 ml/kg/day) for 60 days. As a result, no significant changes in body weight, internal organs, or biochemical and hematological parameters were found (Chaiyasut et al., 2018). Chronic toxicity studies in rats with daily oral administration at doses of 300, 600, and 1,200 mg/kg for 270 days also did not observe obvious histopathology changes (Jaijoy et al., 2010). Moreover, both the micronucleus test in the bone marrow cells of mice and the mouse sperm aberration test suggested that PF had no genotoxicity to somatic or germ cells (Wen et al., 2018). In brief, no toxic effects of PF were observed as demonstrated by hematological examination, behavioral observation, and biochemical parameters suggesting the safe utilization of PF. Thus, PF has high safety for animals and humans under reasonable application, which is very suitable for drugs or healthy product development.

## 7 Quality control of *Phyllanthi Fructus*

Although PF has shown unique efficacy for the treatment of several diseases, there is still a big gap in the industrialization and standardization of PF. Currently, the Chinese Pharmacopoeia (2020 edition) requires that gallic acid in PF (anhydrous substance) is more than 1.2% to ensure medicinal quality (Chinese Pharmacopoeia Commission, 2020), which is still somewhat inadequate. First, gallic acid, found in many plants, is not a chemical component unique to PF. Second, the hydrolyzed tannins contained in PF can be hydrolyzed to gallic acid by heat, affecting the accuracy of the test results (Huang et al., 2019). Therefore, the measurement of gallic acid is not an adequate way to control the quality of PF. Depending on more adequate phytochemical and pharmacological studies of PF, more accurate quality control criteria will be reestablished after the exact active components of PF must be fully studied.

Actually, the quality of PF is closely related to multiple factors, such as germplasm, origin, harvesting, processing, preparation, storage and extraction conditions. There are differences in the quality of PF from different varieties and production areas. HPLC fingerprints were used to confirm that there were significant differences in gallic acid and quercetin in methanol extracts of PF among 6 producing areas (Guo et al., 2013). Salt soaking is one of the traditional processing methods for PF. The contents of gallic acid, ellagic acid and epicatechin were affected by the amount of water, the proportion of salt, and temperature during the salting process (Li et al., 2019). Modern processing methods of PF are mainly based on the removal of impurities and drying. Different drying methods, such as vacuum freeze drying and hot air drying at different temperatures, affected the content of gallic acid, corilagin and polysaccharides in PF (Mao, 2019). In addition, the gallic acid content of PF showed a trend of first increasing and then decreasing during storage. Hence, the single indicator is no longer sufficient to meet the needs of the whole industry chain. The quality evaluation of PF also should pay close attention to the place of origin, planting, production, and processing.

Quality control analysis methods, including Ultra-high performance liquid chromatography-multistage mass spectrometry (UPLC-MS^n^), ultraviolet spectrophotometry, HPLC, and gas chromatogram (GC)/mass spectrometry (MS) are used to analyze the active ingredients of PF. Wu et al. used the UPLC-MS^n^ method to determine the content of tannins and luteolin in PF (Zhang et al., 2019; Wu et al., 2021). This method is high separation efficiency, but it has the disadvantage of time-consuming and costly. The content of gallic acid, epigallocatechin, corilagin, terminalia biphenyl acid, and ellagic acid in PF eluted by ethanol were also measured simultaneously using the HPLC method (Li et al., 2018). Wang et al. identified 43 volatile oil components in PF by GC-MS (Wang et al., 2009). Although the above methods have the advantages of high precision, accuracy, sensitivity, short analysis time, strong separation ability, good selectivity, and wide application range, most methods have the disadvantages of complex sample pretreatment process, expensive equipment, and not being environmental-friendly. A recent study hypothesized that gallic acid, quercetin, ellagic acid, and corilagin could be used as candidate compounds for PF quality markers using predictive analysis with a multi-directional Q-marker (Guan et al., 2022). In the future, Q-marker-based predictive analysis can be studied in depth during the quality control of PF, and methods such as metabolomics and network pharmacology can be applied to it to establish safer, more scientific and effective quality assessment methods.

## 8 Products development of *Phyllanthi Fructus*

**FIGURE 8:**
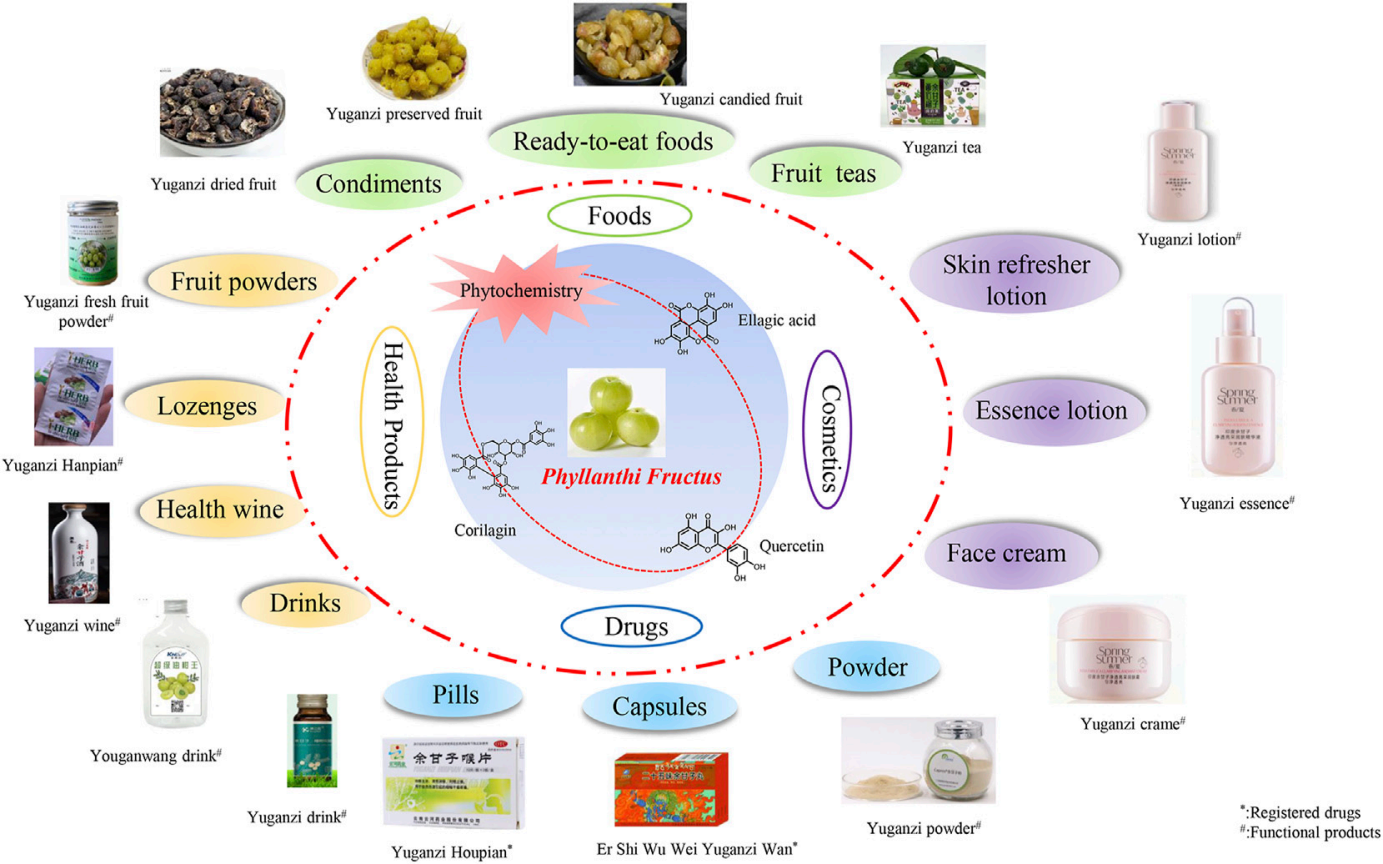
Various products using PF as a raw material.

PF has been developed into various products in many fields due to its various pharmacological activities, including drugs, health products, foods, and cosmetics as shown in Figure 8.

### 8.1 Drugs

PF has been developed into a variety of medicines due to its richness in bioactive substances. For example, PF capsules can reduce the content of TG and TC, increase the content of HDL and the activity of SOD in red blood cells and brain tissue, and reduce the content of lipid peroxides in serum and brain tissue (Li et al., 2002). PF ointment is mainly used for indigestion, abdominal distension, cough, sore throat, and toothache (Yu and Gongbu, 1987). In addition to singular use, PF is often used as a compound prescription. Based on the theory of TCM, it plays different roles when it is compatible with different Chinese herbal medicines. For example, Hepatitis B Kangtai pills are processed and formulated from Chinese medicines including PF, *Morus mulberry, Turmeric, Kochia scoparia, Cao Guo* and *Coptis chinensis*, which can effectively protect the liver and inhibit HBV (He, 2010). The common formulations of PF and traditional Chinese prescriptions that have been clinically tested and approved by the National Medical Products Administration (NMPA) were collected in Table 5.

**TABLE 5:** Traditional Chinese formulations containing PF.

Formulation names	Main composition	Formulations, usage, dosage	Diseases or symptoms therapy	Prescription sources
Er Shi Wu Wei Zheng Zhu Wan (二十五味珍珠丸)	*Pernulo* (20 g), *Myristicae Semen* (40 g), Travertine (100 g), *Fructus* of *Amomum tsaoko Crevost et Lemarie* (30 g), the flower of *Syringa oblata Lindl.* (50 g), the center of the trunk of *Dalbergia odorifera* T. *Chen* (100 g), *Amomi Rotundus Fructus*/*Elettaria cardamomum White* et Maton (40 g), *Fructus of Choerospondias axillaris* (Roxb.)Burtt et Hill (130 g), *Santalum album* L. (50 g), *Phyllanthi Fructus* (100 g), *Aquilaria* spp. (80 g), *Cinnamomi Cortex*/*Cinnamomum tamala* (Bauch.-Ham.) Nees et Eberm (40 g), *Terminaliae Belliricae Fructus* (100 g), Crab (50 g), *Radix Aucklandiae* (80 g), *Malvae Fructus* (40 g), *Piper longum* L. (40 g), *Lagotis brachystachya* Maxim. (100 g), Micae Lapis Aureus (40 g), B*os taurus domesticus Gmelin* (1 g), *Moschus* (1 g), *Cuminum cyminum* L. (60 g), *Carthamus tinctorius* L. (20 g), *Caralluma nebrownii* Bgr. (20 g), *Nigella Sativa* seed (30 g)	Pill/oral administration/bid-tid/1.2–1.5 g each time	Used for cerebral hemorrhage, cerebral infarction, poststroke syndrome, myocardial infarction and angina pectoris	Si Bu Yi Dian. AD773–783. *Chinese Pharmacopoeia* (2020)
Shi Ba Wei Du Juan Wan (十八味杜鹃丸)	*Rhododendron anthopogonoides* Maxim. (75 g), *Fructus* of *Amomum tsaoko* Crevost et Lemarie (15 g), *Fructus* of *Terminalia chebula* Retz. (70 g), *Fructus* of *Terminalia bellirica* (Gaertn.) Roxb. (50 g), *Phyllanthi Fructus* (60 g), the lumps in the stalks of *Bambusa textilis Mc*Clure/*Schizostachyum chinense* Rendle (35 g)*,* The dried flower of *Carthamus tinctorius* L. (50 g), the dried seed of *Myristica fragrans* Houtt. (15 g), the dried flower buds *Eugenia caryophμllata*Thunb. (20 g), *Fructus* of *AmomumkravanhPierre* ex Gagnep. (15 g), the center of the trunk of a *Santalum album* L*.* (30 g), the trunk of *Pterocarpus santalinus* L.f. (50 g), the leaves of *Symplocos sumuntia* Buch-Ham. ex D. Don (40 g), *Rubia cordifolia* L. (35 g), the secretion of *Lacciferlacca* Kerr. (40 g), *Gentiana straminea* Maxim. (35 g), *Glycyrrhiza uralensis* Fisch. (40 g), the wood of *Aquilaria sinensis* (Lour.) Gilg (50 g)	Pill/oral administration/tid/2g–2.5 g each time	Used for muscular dystrophy, numbness of limbs, facial distortion, dysmenorrhea and amenorrhea	NMPA^a^. Tibetan Medicon^b^
Shi Wu Wei Cheng Xiang Wan. (十五味沉香丸)	*Aquilaria* spp. (100 g), *Inula graveolens* (150 g), *Santalum album* L. (50 g), *Pterocarpus indicus willd* (150 g), the flower of *Carthamus tinctorius* L. (100 g), *Myristica fragrans* (25 g), Rhizome of Pegaeophyton scapiflorum (Hook.f.et Thoms)Marq.et Shaw (150 g), Stem of Rubu*s* L. (200 g), *Fallopia aubertii* (L. Henry) Holub (peeled) (100 g), *Asarum canadense* (50 g), Travertine (100 g), *Fructus* of *Choerospondias axillaris* (Roxb.) Burtt et Hill (150 g), *Fructus* of *Terminalia chebula* Retz. (80 g), *Phyllanthi Fructus* (100 g)	Pill/oral administration/tid/1.5 g each time	Used for coordinating qi and blood, relieving cough, and soothing the nerves. Treatment of qi stagnation and blood stasis, dry cough, shortness of breath, and insomnia	*Chinese Pharmacopoeia* (2020)
Ba Wei An Ning San (八味安宁散)	*Gypsum*/*Calcite* (400 g), limonite (100 g), the seed of *Sinapis alba* L. (350 g), *Dracocephalum tanguticum* Maxim (10 g), the root of *Aconitum pendulum* Busch (10 g), Fructus of Choerospondias axillaris (Roxb.) Burtt et Hill (10 g), Fructus of *Terminalia* chebula Retz. (10 g), Phyllanthi Fructus (10 g)	Decoction/oral administration/bid-tid/1 g each time	Used for menorrhagia, metrorrhagia and metrostaxis, dyspepsia	NMPA^a^. Tibetan Medicon^b^
Si Wei Jang Huang Tang San. (四味姜黄汤散)	*Curcuma longa* (15 g), The dry bark of *Berberis kansuensis Schneid*. (12.5 g), *Phyllanthi Fructus* (25 g), *Fructus* of *Cenchrus echinatus* Linn. (25 g)	Decoction/oral administration/bid/3.2g–4.8 g each time	Used for urethritis, frequent urination and urgency. Clearing away heat and diuresis	*Chinese Pharmacopoeia* (2020). NMPA^a^
Liu Wei Yu Gan Zi Tang San (六味余甘子汤散)	*Phyllanthi Fructus* (200 g), *Fructus* of *Coriandrum sativum* L. (75 g), *Fructus* of *Malva verticillata* var. *Crispa* Linnaeus (75 g), the root of *Pedicularis resupinata* L. (40 g), *Oxytropis kansuensis* Bunge (100 g), the root and stem of *Glycyrrhiza uralensis* Fisch./*Glycyrrhiza inflata* Bat./*Glycyrrhiza glabra* L. (50 g)	Decoction/oral administration/bid/3 g each time	Used for clearing heat and diuresis	Shang Han Lun Ji Yi. AD1801
Ba Wei Xiao Bo Pi San (八味小檗皮散)	The roots and stems of *Berberis silva-taroucana* Schneid. (150 g), the fruit ear *Piper longum* L. (20 g), *Phyllanthi Fructus* (125 g), the root and stem of *Glycyrrhiza uralensis* Fisch./*Glycyrrhiza inflata* Bat./*Glycyrrhiza glabra* L. (50 g), *Crocus sativus* L. (7.5 g), *Ursi Fellis Pulvis* (6 g), the secretion of *Moschus berezovskii*/*M.sifanicus Przewalski*/*M.Moschiferus Linnaeus* (6 g), Jing Mo (12 g)	Decoction/oral administration/bid/1 g each time	Used for urinary tract infection, odynuria, hematuresis, gonorrhea and spermatorrhea	*Chinese Pharmacopoeia* (2020). Tibetan Medicon^b^
Qi Zhen Tang San (七珍汤散)	The root of *Inula helenium* L. (70 g), the stem of *Rubus* L. (225 g), the stem of *Tinospora sinensis* (Lour.) *Merr.* (125 g), the root and stem of *Zingiber oj-jicinale* Rosc. (50 g), *Fructus* of *Choerospondias axillaris* (Roxb.)Burtt et Hill (90 g), *Fructus* of *Terminalia chebula* Retz. (50 g), *Phyllanthi Fructus* (100 g)	Decoction/oral administration/bid/3 g each time	Used for common cold due to wind-cold, fever and arthralgia	NMPA^a^ Tibetan Medicon^b^
Qi Wei Kuan Jin Teng Tang San (七味宽筋藤汤散)	The vine stem of *Tinospora sinensis (Lour.) Merr.* (30 g), *Fructus* of *Choerospondias axillaris* (Roxb.)Burtt et Hill (25 g), *Fructus* of *Terminalia chebula Retz.* (80 g), *Phyllanthi Fructus* (25 g), *Swertia bimaculata* (Sieb. Et Zucc.) Hook. f. et Thoms. Ex C. B. Clark)(25 g), *Corydalis impatiens* (pall.) Fisch. (25 g), *Rhodiola crenulata* (Hook. f. et Thoms.) H. Ohba (25 g)	Decoction/oral administration/bid/3 g each time	Used for heat-clearing and detoxifying, variola	Tibetan Medicon^b^
Jie Ke Bai Bei Wan (解渴百杯丸)	*Fructus* of *Chaenomeles speciosa* (Sweet)Nakai (2000g), *Prunus mume* (Sieb.)Sieb.etZuce. (500 g), the root and stem of *Glycyrrhiza uralensis* Fisch./*Glycyrrhiza inflata* Bat./*Glycyrrhiza glabra* L. (375 g), the root of *Pueraria lobata* (Wild) Ohwi/*Pueraria thomsonii* Benth. (100 g), *Ligusticum chuanxiong hort* (25 g), *Phyllanthi Fructus* (25 g), the leaves of *Perilla/rMtescews* (L.) Britt. (25 g), *Mass Galla chinesis et camelliae Fermentata* (50 g), *Sal* (500 g)	Pill/oral administration/1 pill each time	Used for clearing heat and detoxication	Pu Ji Fang. AD1406
Qi Wei Xiao Zhong Wan (七味消肿丸)	*Phyllanthi Fructus* (300 g), the flower of *Carthamus tinctorius* L. (150 g), the seed of *Herpetospermum pedunculosum* (Ser.) C.B.Clarke (40 g), *Dracocephalum tanguticum* Maxim (200 g), *Veronica eriogyne* H.Wink1. (80 g), *Aconitum naviculare* (Bruhl.) Stapf (100 g), the flower of *Meconopsis* Vig. (150 g)	Pill/oral administration/bid-tid/2g–2.5 g each time	Used for edema, thirst, oliguria, asthma and ascites	NMPA^a^. Tibetan Medicon^b^
Shi Wei Ke Zi Tang San (十味诃子汤散)	*Fructus* of *Choerospondias axillaris* (Roxb.) Burtt et Hill (15 g), *Fructus* of *Terminalia chebula* Retz. (10 g), *Phyllanthi Fructus* (15 g), the stem of *Tinospora sinensis* (Lour.) Merr. (15 g), *Swertia bimaculata* (Sieb. Et Zucc.) Hook. f. et Thoms. Ex C. B. Clark (15 g), *Aconitum naviculare* (Bruhl.) Stapf (15 g), *Lagotis brevituba Maxim.* Bull. Acad. St. Petersb. (15 g), the root and stem of *Rheum palmatum* L. (15 g), *Fructus* of *Cassia fistula* L. (10 g), the root of *Euphorbia pekinensis* Rupr. (10 g)	Decoction/oral administration/bid/1.6 g each time	Used for hepatitis B	Tibetan Medicon^b^
Shi Wei Xue Re Tang San (十味血热汤散)	*Fructus* of *Choerospondias axillaris* (Roxb.) Burtt et Hill (175 g), *Fructus* of *Terminalia chebula* Retz. (75 g), *Phyllanthi Fructus* (150 g), Radix Onosmatis/*Onosma paniculatum* Bur. Et Fr. (100 g), the secretion of *Lacciferlacca* Kerr. (75 g), the stem of *Tinospora sinensis* (Lour.) Merr. (200 g), the root of *Inula helenium* L. (150 g), *Radix et Rhizoma Rubiae*/*Rubia wallichiana* Decne. (75 g), the root of *Rubus biflorus* Buch-Ham. ex Smith (300 g), *Zingiberis Rhizoma*/*Zingiber officinale* Rosc. (50 g)	Decoction/oral administration/bid/5 g each time	Used for clearing heat, dizzy, mouth parched and tongue scorched	NMPA^a^. Tibetan Medicon^b^
Qi Wei Xue Bing Wan (七味血病丸)	*Radix Arnebiae*/*Lithospermum erythrorhizon* Sieb. Et Zucc. (100 g), calcite (75 g), *Phyllanthi Fructus* (85 g), the root of *Inula helenium* L. (150 g), the root of *Auckiandialappa* Decne. (40 g), the root and stem of *Rubia cordifolia* L. (35 g), *Veronica didyma* Tenore (50 g)	Decoction/oral administration/qd-bid/1g–2 g each time	Used for clearing heat and expectoration, removing heat from the lung to relieve cough, throat edema and chest stuffiness	Tibetan Medicon^b^

^a^ Cited from the website: https://www.nmpa.gov.cn/.

^b^ Cited from the website: http://tibetmdc.com/.

PF is an herbal remedy with multiple healing properties for a wide range of diseases. After extensive basic research, researchers have conducted several clinical trials to scientifically validate the remarkable therapeutic effects of PF. In clinical trials for different diseases, it is often used alone or as the main ingredient in combination formulations. Several clinical trials of PF for different disease were listed in Table 6.

**TABLE 6:** Clinical trials of drugs related to PF.

Diseases	Investigational drug and extracts	Formulations, dosage and administration	Positive/placebo control	Clinical trials	Outcome indicators	References
**Dyslipidemia**	PF extracts capsule (35% polyphenols, 8% triterpenoids and 10% oil). Ethyl acetate. Amlamax™ (30% gallo ellagi tannins). Aqueous	Capsule/oral administration/bid/500°mg each time for 12°weeks. Tablet/oral administration/qd/500mg- 1,000°mg for 6°months	Roasted rice powder (bid/500°mg each time p.o.)_	A randomized, double-blind, placebo, controlled, multicenter clinical trial. Total Subjects:98 (45 males and 53 females). PF group:49 placebo groups: 49. Pilot Clinical study. Total Subjects: 51. Group I: 26 (Amlamax 500°mg; Group II: 25 (Amlamax 1,000°mg)	TC↓, TG↓, VLDL-C↓, atherogenic index of the plasma (AIP)↓, the ratio of Apo B to Apo A1↓. Group I and II: total and LDL cholesterols↓, HDL cholesterol↑	Upadya et al. (2019). Antony et al. (2008)

**Type 2 diabetic**	PF dried powder (Undefined Proportion). _	Tablet/oral administration/bid/1, 2, or 3°g each time for 21°days	Glibenclamide (bid/5°mg each time p.o.)	Comparative randomized clinical trial. Total Subjects: 32. Normal group: three subgroups (1.2,3°g) and control group (carboxymethyl cellulose fiber). Diabetic group : three subgroups and control group (glibenclamide orally)	1.2,3°g subgroups: fasting and 2-h postprandial blood glucose levels ↓. 2.3°g subgroup: total cholesterol and TG↓, HDL-C↑, LDL-C↓ on day 21. 3°g group: total lipids↓	Akhtar et al. (2011)
**Chronic periodontitis**	PF gel (10% sustained-release gel, 20% Poloxamer 407, 4% Carbopol 940 polymers and 10% PF extracts). Ethanol	Gel/ext. administration/qd-tid/subgingival application for 3°months	Placebo gel. (qd-tid/subgingival application for 3°months)	A Randomized Placebo-controlled Clinical Trial. Total Subjects:46 patients (528 sites). Control group (23; 264): SRP + placebo gel test group; Test group(23; 264) : SRP +10%PF gel application	Mean probing pocket depth↓, modified sulcus bleeding index↓, clinical attachment level↓	Grover et al. (2016)
**Melasma**	Three herb cream (PF, Licorice and Belides depigmenting complex 7%). Ethanol	Cream/ext. administration/bid/60°days	Hydroquinone cream 2% (qd/ext.)	Mono-blind clinical study. Total subjects: 56 women,18–60°years of age. Group A: 23, cream with Belides, Emblica and Licorice, applied twice a day; Group B: 23, cream with Hydroquinone 2%, used at night	Group A showed fewer skin adverse events	Costa et al. (2010)
**Essential hypertension**	PF dry powder capsule (Undefined Proportion). Aqueous	Capsule/oral administration/bid/500°mg each time for 12°weeks	Maize starch IP grade (bid/500°mg each time p.o.)	A randomized double-blind placebo-controlled Tria. Total subjects:150. Test group:75; Placebo group : 75, maize starch IP grade	No significant change in systolic and/or diastolic blood pressure levels, lipid profile, HbA1C, hs-CRP levels	Shanmugarajan et al. (2021)
**Symptomatic knee osteoarthritis**	SGC^a^ and SGCG^a^ formulations (Undefined Proportion)_	Capsule/oral administration/qd/400°mg each time for 24°weeks	Glucosamine sulphate (qd/2°g each time p.o.) and celecoxib (qd/200°mg each time p.o.)	A randomized, double-blind, controlled equivalence drug trial. Total subjects:440. Group 1:110, SGCG^a^; Group 2: SGC^a^. Group 3:10, glucosamine sulphate. Group 4: 110, celecoxib	Knee pain↓, knee function↑	Chopra et al. (2013)

^a^ Each SGCG, capsule (400°mg) contained Zingiber officinale, Tinospora cordifolia, PF, and B. serrata. The SGC, capsule (400°mg) was similar to SGCG (both for content and quantity) except for the absence of B. serrata extracts and a higher quantity of excipients.\ "-" the experimental study has no positive/placebo control.

### 8.2 Health products

A wide range of health products has been developed using PF as a raw material. First, health care wines and beverages, including PF health wine, PF health ginger wine (Ming and Wang, 2018), Sanzi health drink, spiral health drink, and PF functional oral liquid. Wine prevents or reduces the development of atherosclerosis and coronary heart disease by increasing the level of HDL in the body. Brewing PF into health care wine could exert better therapeutic efficacy. Second, fruit powders. PF nutritional powders retain the SOD and over 70% of the vitamin C contained in the fresh fruit. Fruit powder is also suitable for long-term consumption by the elderly. It has the effects of clearing heat, lowering blood pressure, preventing liver and gallbladder diseases, and strengthening immunity. Third, lozenges, such as PF lozenge and PF lemon health-care lozenge, are rich in vitamins, amino acids, and a variety of trace elements. It has positive effects on the treatment of cough, obesity and cardiovascular diseases (Xu, 2007).

### 8.3 Foods

Ready-to-eat food prepared from fresh PF, including PF jam, preserved fruit, fructose, fruit cake, jelly, and yogurt. These products are more acceptable to the public because they retain the unique flavour and nutrients of PF while removing the sourness and astringency. A range of compound drinks and teas made from PF, including PF juice, lemon drink, *Rosa roxburghii Tratt* fruit drink, fruit tea, instant oolong tea, and herbal tea. These compound drinks taste good and achieve specific health benefits (Cao et al., 2019). PF when incorporated with ice cream in processed form resulted in increasing melting resistance and decreasing overrun, thereby enhancing the functional property and nutritional value of ice cream (Goraya and Bajwa, 2015). In addition, PF is made into condiments, health-care noodles and other products, retaining some of its medicinal properties.

### 8.4 Cosmetics

Herbs have long been used in medicines and cosmetics due to their potential to treat skin ailments and improve skin appearance. Antioxidants, flavonoids, and phenolic compounds present in PF play critical roles to counteract free radicals, the main cause of various unfavorable skin changes. The results of Fujii et al. revealed stimulated proliferation of fibroblast and controlled collagen metabolism confirmed the mitigative, cosmetic, and therapeutic application of PF (Fujii et al., 2008). Therefore, PF has been added to sunscreens, anti-aging and whitening skincare products and cosmetics. For example, a cosmetic developed from the raw materials of PF, lotus, tuckahoe, schisandra, and mulberry bark extractss can inhibit the formation of melanin (Chen, 2019). These cosmetics meet the skincare needs of people for facial skin whitening, anti-aging, and wrinkle removal.

## 9 Discussion and perspective

The value of phytomedicine is well recognized in China and South East Asia followed by the continuously increasing demand worldwide. PF, rich in tannins, phenolic acids, flavonoids, polysaccharides, and vitamin, has been shown to have a variety of pharmacological effects on the respiratory, digestive, nervous, and cardiovascular systems, which are attributed to its anti-inflammatory, antioxidant, antiviral, and anti-apoptotic properties. Modern pharmacological studies have gradually confirmed some traditional effects of PF, such as moistening the lung for arresting cough, clearing heat and detoxifying, lowering blood pressure and glucose, *etc.* Moreover, some novel effects of PF were identified with modern pharmacology, including anti-anxiety, anti-depression, and neuroprotective effects. These pharmacological and toxicologic studies provide an important scientific basis for the development of PF-related products. In addition to being developed as a variety of drugs, PF has also been developed in the food, health care products, and cosmetics products industries.

Although it has been widely studied using modern technology, there are still challenges in the development of PF in a sustainable and maximally beneficial way. For instance, both cultivated and wild PF have the problem of varying quality. PF that does not meet the medicinal standard is not fully utilized, which easily results in a huge waste of resources. Therefore, it is of great significance to establish the medicinal and food grading utilization system of PF. At present, the research and application of *Phyllanthus Emblica* L. mainly focus on mature fruits, but the research on its roots, stems and leaves is less. Through deepening the research on the chemical composition and pharmacological activity of other medicinal parts of *Phyllanthus Emblica* L*.*, the comprehensive utilization of *Phyllanthus Emblica* L. plants as a viable resource can eventually effectively promote the economic development of *Phyllanthus Emblica* L*.* production area. In addition, gallic acid is specified as a quality control index of PF in the Chinese Pharmacopoeia (version 2020). The single quality control index of PF is not representative of the overall quality of PF. A more scientific, simple, and easily replicable quality evaluation method needs to be sought in the future for PF industrialization.

## Glossary

ABTS+: 2,2′-azinobis-(3-ethylbenzothiazoline-6-sulfonic acid)
AchE: acetylcholinesterase; AgNPs, synthesize silver nanoparticles
AIDS: anti-acquire immunodeficiency syndrome
ALT: alanine aminotransferase
AMPK: adenosine 5‘-monophosphate-activated protein kinase
AR: Amalaki Rasayana
AST: aspartate aminotransferase
CAT: catalase
COX-2: cyclooxygenase-2
DPPH: 2, 2 diphenyl-1-picrylhydrazyl
GC/MS: gas chromatogram/mass spectrometry
GRO-α: growth-regulated oncogene alpha
GSH-Px: glutathione peroxidase
HDL: high-density lipoprotein
HFD: high-fat diet
HGSOC: high-grade serous ovarian cancer
HIV: human immunodeficiency virus
HPLC: high performance liquid chromatography
HPV: human papillomavirus
HSV-1: herpes simplex virus type 1
IL-6: interleukin-6
IL-8: nterleukin-8
INF-α: tumour necrosis factor-α
IRI: ischaemia-reperfusion injury
LDH: lactate dehydrogenase
LDL: low-density lipoprotein
LPS: Lipopolysaccharide
MDA: malondialdehyde
MgO: magnesium oxide
MIP-2: macrophage inflammatory protein 2
NF-κB: nuclear factor kappa-B
NK: natural killer
NSAIDs: non-steroidal anti-inflammatory drugs
OXPHOS: oxidative phosphorylation
PAO1: pseudomonas aeruginosa 1
PF: Phyllanthi Fructus
PGE2: prostaglandin E2
ROS: reactive oxygen species
SARS-CoV-2: severe acute respiratory syndrome coronavirus 2
SNI: a neuropathic pain nerve injury
SOD: super oxide dismutase
STZ: streptozotocin
TC: total cholesterol
TCM: traditional Chinese medicine
TG: triglyceride
UPLC-MSn: Ultra-high performance liquid chromatography-multistage mass spectrometry; triglyceride
VLDLs: very-low-density lipoproteins

## References

[B1] Alamand M. I. Gomes A. (2003). Snake venom neutralization by Indian medicinal plants (Vitex negundo and Emblica officinalis) root extracts. J. Ethnopharmacol. 86 (1), 75–80. 10.1016/s0378-8741(03)00049-7 12686445

[B165] Akhtar M. S. Ramzan A. Ali A. Ahmad M. (2011). Effect of amla fruit (emblica officinalis gaertn.) on blood glucose and lipid profile of normal subjects and type 2 diabetic patients. Int. J. Food Sci. Nutr. 62 (6), 609–616. 10.3109/09637486.2011.560565 21495900

[B2] Anila L. Vijayalakshmi N. R. (2002). Flavonoids from emblica officinalis and mangifera indica-effectiveness for dyslipidemia. J. Ethnopharmacol. 79 (1), 81–87. 10.1016/S0378-8741(01)00361-0 11744299

[B3] Antony B. Benny M. Kaimal T. N. B. (2008). A Pilot clinical study to evaluate the effect of Emblica officinalis extract (Amlamax™) on markers of systemic inflammation and dyslipidemia. Indian J. Clin. biochem. 23 (4), 378–381. 23105791 10.1007/s12291-008-0083-6 PMC3453138

[B4] Arora D. S. R. Sharma S. Suttee A. (2012). Phytochemical and microscopical investigations on Emblica offi cinalis Gaertn. Int. J. Pharmac Phytoch Res. 4, 1–4. 10.1007/BF03051560

[B5] Athira A. P. Helen A. Saja K. Reddanna P. Sudhakaran P. R. (2013). Inhibition of angiogenesis *in vitro* by chebulagic acid: A COX-LOX dual inhibitor. Int. J. Vasc. Med. 2013, 843897. 10.1155/2013/843897 24288615 PMC3833124

[B6] Avula B. Wang Y. H. Wang M. Shen Y. H. Khan I. A. (2013). Simultaneous determination and characterization of tannins and triterpene saponins from the fruits of various species of terminalia and phyllantus emblica using a uhplc-uv-ms method: Application to triphala. Planta Med. 29 (02), 181–188. 10.1055/s-0032-1328089 23299756

[B7] Bai H. N. (2019). Progress in the isolation and purification of polysaccharides and their biological activities. Mod. Food (17), 10–11+14. 10.16736/j.cnki.cn41-1434/ts.2019.17.003

[B8] Balusamy S. R. Veerappan K. Ranjan A. Kim Y. J. Chellappan D. K. Dua K. (2020). Phyllanthus emblica fruit extract attenuates lipid metabolism in 3T3-L1 adipocytes via activating apoptosis mediated cell death. Phytomedicine 66, 153129. 10.1016/j.phymed.2019.153129 31794911

[B9] Bandyopadhyay S. K. Pakrashi S. C. Pakrashi A. (2000). The role of antioxidant activity of phyllanthus emblica fruits on prevention from indomethacin induced gastric ulcer. J. Ethnopharmacol. 70 (2), 171–176. 10.1016/S0378-8741(99)00146-4 10771207

[B11] Bharathi M. D. Thenmozhi A. J. Manivasagam T. Rather M. A. Babu C. S. Essa M. M. (2019). Amelioration of aluminum maltolate-induced inflammation and endoplasmic reticulum stress-mediated apoptosis by tannoid principles of emblica officinalis in neuronal cellular model. Neurotox. Res. 35, 318–330. 10.1007/s12640-018-9956-5 30242626

[B12] Bhatia J. Tabassum F. Sharma A. K. Bharti S. Golechha M. Joshi S. (2011). Emblica officinalis exerts antihypertensive effect in a rat model of DOCA-salt-induced hypertension: Role of (p) eNOS, NO and oxidative stress. Cardiovasc. Toxicol. 11 (3), 272–279. 10.1007/s12012-011-9122-2 21748534

[B13] Bhattacharya A. Kumar M. Ghosal S. Bhattacharya S. K. (2000a). Effect of bioactive tannoid principles of emblica officinalis on iron-induced hepatic toxicity in rats. Phytomedicine 7 (2), 173–175. 10.1016/S0944-7113(00)80091-4 10839222

[B14] Bhattacharya S. K. Bhattacharya D. Muruganandam A. V. (2000b). Effect of Emblica officinalis tannoids on a rat model of tardive dyskinesia. Indian J. Exp. Biol. 38 (9), 945–947. 10.1258/0956462001915282 12561957

[B15] Bobasa E. M. Phan A. Netzel M. E. Cozzolino D. Sultanbawa Y. (2021). Hydrolysable tannins in Terminalia ferdinandiana Exell fruit powder and comparison of their functional properties from different solvent extracts. Food Chem. 358 (8), 129833. 10.1016/j.foodchem.2021.129833 33933967

[B16] Cao B. He S. Z. Jing W. J. (2019). Research on the nutritional value and current status of processing and utilization of Phyllanthus emblica. Mod. Food 000 (004), 1–4.

[B17] Chaikul P. Kanlayavattanakul M. Somkumnerd J. Lourith N. (2021). Phyllanthus emblica L.(amla) branch: A safe and effective ingredient against skin aging. J. Tradit. Complement. Med. 11 (5), 390–399. 10.1016/j.jtcme.2021.02.004 34522633 PMC8427479

[B18] Chaiyasut C. Sivamaruthi B. S. Duangjitcharoen Y. Kesika P. Sirilun S. Chaiyasut K. (2018). Assessment of biological safety of fermented Phyllanthus emblica fruit juice. Asian J. Pharm. Clin. Res. 11 (9), 312–316. 10.22159/ajpcr.2018.v11i9.27104

[B19] Chandak P. G. Gaikwad A. B. Tikoo K. (2009). Gallotannin ameliorates the development of streptozotocin-induced diabetic nephropathy by preventing the activation of PARP. Phytother. Res. 23 (1), 72–77. 10.1002/ptr.2559 18693296

[B20] Chao C. Y. Mong M. C. Chan K. C. Yin M. C. (2010). Anti-glycative and anti-inflammatory effects of caffeic acid and ellagic acid in kidney of diabetic mice. Mol. Nutr. Food Res. 54 (3), 388–395. 10.1002/mnfr.200900087 19885845

[B21] Chatterjee A. Chatterjee S. Biswas A. Bhattacharya S. Bandyopadhyay S. K. (2012). Gallic acid enriched fraction of phyllanthus emblica potentiates indomethacin-induced gastric ulcer healing via e-nos-dependent pathway. Evid. Based. Complement. Altern. Med. 2012 (4), 487380. 10.1155/2012/487380 PMC3433150 22966242

[B22] Chatterjee A. Chattopadhyay S. Bandyopadhyay S. K. (2011). Biphasic effect of Phyllanthus emblica L. Extract on NSAID-induced ulcer: An antioxidative trail weaved with immunomodulatory effect. Evid. Based Complement. Altern. Med. 2011, 146808. 10.1155/2011/146808 PMC2976071 21076542

[B23] Chaudhuri R. K. (2004). PCIA conference.

[B24] Chen A. Jing Y. (2012). Effect of Miao medicine Yuganzi mixture on pulmonary function in patients with acute exacerbation of chronic obstructive pulmonary disease. Zhongwai Jiankang Wenzhai 09 (002), 53–54. 10.3969/j.issn.1672-5085.2012.02.044

[B25] Chen G. J. Kan J. Q. (2018). Characterization of a novel polysaccharide isolated from Rosa roxburghii Tratt fruit and assessment of its antioxidant *in vitro* and *in vivo* . Int. J. Biol. Macromol. 107, 166–174. 10.1016/j.ijbiomac.2017.08.160 28866014

[B27] Chen J. Feng G. Y. Li D. K. (2016). Effects of tannin part of Tibetan medicine PF on apoptosis and cell cycle of human tumour cells. Chin. Pharmacovigil. 13 (04), 193–196. 10.19803/j.1672-8629.2016.04.001

[B28] Chen K. H. Lin B. R. Chien C. T. Ho C. H. (2011). Emblica officinalis gaertn. attentuates n-nitrosodiethylamine-induced apoptosis, autophagy, and inflammation in rat livers. J. Med. Food 14 (7-8), 746–755. 10.1089/jmf.2010.1459 21761987

[B29] Chen P. (2019). A cosmetic with whitening and moisturizing functions and its preparation process.

[B30] Chen Y. J. Long X. Y. Pan S. J. An X. Chen S. L. (2013). Progress in the study of pharmacodynamic mechanisms and conformational relationships of flavonoids. Chin. J. Exp. Formulation 19 (11), 337–343.

[B31] Chen Z. Y. Liu X. M. Wu J. J. (2003). Biological characteristics and nutritional components of Glycyrhiza sinensis. Zhongguo Nanfang Guoshu 032 (006), 71–73. 10.3969/j.issn.1007-1431.2003.06.045

[B32] Chinese Pharmacopoeia Commission (2020). Chinese Pharmacopoeia. Beijing: China Medical And Technology Press.

[B33] Chopra A. Saluja M. Tillu G. Sarmukkaddam S. Venugopalan A. Narsimulu G. (2013). Ayurvedic medicine offers a good alternative to glucosamine and celecoxib in the treatment of symptomatic knee osteoarthritis: A randomized, double-blind, controlled equivalence drug trial. Rheumatol. Oxf. 52 (8), 1408–1417. 10.1093/rheumatology/kes414 23365148

[B34] Chuang H. Y. Ng L. T. Lin L. T. Chang J. S. Chen J. Y. Lin T. C. (2011). Hydrolysable tannins of tropical almond show antifibrotic effects in TGF-β1-induced hepatic stellate cells. J. Sci. Food Agric. 91 (15), 2777–2784. 10.1002/jsfa.4521 21725979

[B36] Costa A. Moisés T. A. Cordero T. Alves C. Marmirori J. (2010). Association of emblica, licorice and belides as an alternative to hydroquinone in the clinical treatment of melasma. An. Bras. Dermatol. 85 (5), 613–620. 10.1590/S0365-05962010000500003 21152784

[B37] Cui B. Q. He Z. Y. Yang Z. M. (2010). Effects of Phyllanthus emblica extracts on immune function of mice. Lishizhen Med. Materia Medica Res. 21 (008), 1920–1922. 10.3969/j.issn.1008-0805.2010.08.033

[B246] Daisy P. Babu A. S. Modilal R. (2009). Antihyperglycemic effects of Phyllanthus extracts in alloxan-induced diabetic rats.

[B38] D'Souza J. J. D'Souza P. P. Fazal F. Kumar A. Bhat H. P. Baliga M. S. (2014). Anti-diabetic effects of the Indian indigenous fruit emblica officinalis gaertn: Active constituents and modes of action. Food Funct. 5 (4), 635–644. 10.1039/c3fo60366k 24577384

[B39] Dastur J. F. (1952). Medicinal plants of India and Pakistan. Bombay: D. B. Taraporevala Sons & Co., Ltd.

[B40] De A. De A. Sharma R. Suo W. Sharma M. (2020). Sensitization of carboplatinum- and taxol-resistant high-grade serous ovarian cancer cells carrying p53, BRCA1/2 mutations by emblica officinalis (amla) via multiple targets. J. Cancer 11 (7), 1927–1939. 10.7150/jca.36919 32194804 PMC7052860

[B41] De A. Papasian C. Hentges S. Banerjee S. Haque I. Banerjee S. K. (2013). Emblica officinalis extract induces autophagy and inhibits human ovarian cancer cell proliferation, angiogenesis, growth of mouse xenograft tumors. PLoS One 8 (8), e72748. 10.1371/journal.pone.0072748 24133573 PMC3794841

[B42] Dhingra D. Joshi P. Gupta A. Chhillar R. (2012). Possible involvement of monoaminergic neurotransmission in antidepressant-like activity of Emblica officinalis fruits in mice. CNS Neurosci. Ther. 18 (5), 419–425. 10.1111/j.1755-5949.2011.00256.x 22070517 PMC6493501

[B43] Ding Y. Sun X. Chen Y. Deng Y. Qian K. (2015). Epigallocatechin gallate attenuated non-alcoholic steatohepatitis induced by methionine- and choline-deficient diet. Eur. J. Pharmacol. 761, 405–412. 10.1016/j.ejphar.2015.05.005 25967348

[B44] Dong K. Li C. W. Qu H. (2009). Effect of phyllanthus emblica L on the expression of protein kinase B and glucose transporter4 mRNA in the skeletal muscle of insulin resistance rat. J. Fourth Mil. Med. Univ. 030 (021), 2352–2355.

[B45] Dong Y. H. Yin S. T. Jiang C. Luo X. H. Guo X. Zhao C. (2014). Involvement of autophagy induction in penta-1, 2, 3, 4, 6-O-galloyl-beta-D-glucose-induced senescence-like growth arrest in human cancer cells. Autophagy 10 (2), 296–310. 10.4161/auto.27210 24389959 PMC5396085

[B46] Du Q. Xu X. H. Lin P. C. Yong C. L. Ju Y. E. (2017). Research progress and developmental prospect of plant polysaccharide. Electron. J. Transl. Med. 10.3969/j.issn.2095-6894.2017.04.020

[B47] Du R. Cooper L. Chen Z. Lee H. Rong L. Cui Q. (2021). Discovery of chebulagic acid and punicalagin as novel allosteric inhibitors of SARS-COV-23CLpro. Antivir. Res. 190, 105075. 10.1016/j.antiviral.2021.105075 33872675 PMC8052511

[B48] El-Mekkawy S. Meselhy M. R. Kusumoto I. T. Kadota S. Hattori M. Namba T. (1995). Inhibitory effects of Egyptian folk medicines on human immunodeficiency virus (HIV) reverse transcriptase. Chem. Pharm. Bull. 43 (4), 641–648. 10.1248/cpb.43.641 7541317

[B49] Ernst E. (1999). Medicinal plants of the world. Focus Altern. Complement. Ther. 4, 153. 10.1111/j.2042-7166.1999.tb01066.x

[B50] Flora of China Editorial Committee (1998). Flora of China. Beijing, China: Science Press, 526–527.

[B51] Fujii T. Wakaizumi M. Ikami T. Saito M. (2008). Amla (Emblica officinalis Gaertn.) extract promotes procollagen production and inhibits matrix metalloproteinase-1 in human skin fibroblasts. J. Ethnopharmacol. 119 (1), 53–57. 10.1016/j.jep.2008.05.039 18588964

[B52] Ganesan R. (2003). Identification, distribution and conservation of Phyllanthus indofischeri, another source of Indian gooseberry. Curr. Sci. 84, 1515–1518.

[B54] Ghosal S. Tripathi V. K. Chauhan S. (1996). Active constituents of Emblica officinalis:part 1-the chemistry and antioxidative effects of two new hydrolysable tannins, Emblicanin A and B. Indian J. Chem. B 35 (9), 941–948. 10.1007/BF00499557

[B55] Golechha M. Bhatia J. Arya D. S. (2010). Hydroalcoholic extract of Emblica officinalis Gaertn. affords protection against PTZ-induced seizures, oxidative stress and cognitive impairment in rats. Indian J. Exp. Biol. 48 (5), 474–478. 20795364

[B56] Golechha M. Bhatia J. Ojha S. Arya D. S. (2011). Hydroalcoholic extract of emblica officinalis protects against kainic acid-induced status epilepticus in rats: Evidence for an antioxidant, anti-inflammatory, and neuroprotective intervention. Pharm. Biol. 49 (11), 1128–1136. 10.3109/13880209.2011.571264 21749189

[B57] Golechha M. Sarangal V. Ojha S. Bhatia J. Arya D. S. (2014). Anti-inflammatory effect of emblica officinalis in rodent models of acute and chronic inflammation: Involvement of possible mechanisms. Int. J. Inflam. 2014, 178408. 10.1155/2014/178408 25215258 PMC4158298

[B58] Gopa B. Bhatt J. Hemavathi K. G. (2012). A comparative clinical study of hypolipidemic efficacy of Amla (Emblica officinalis) with 3-hydroxy-3-methylglutaryl-coenzyme-A reductase inhibitor simvastatin. Indian J. Pharmacol. 44 (2), 238–242. 10.4103/0253-7613.93857 22529483 PMC3326920

[B59] Goraya R. K. Bajwa U. (2015). Enhancing the functional properties and nutritionalquality of ice cream with processed amla (Indian gooseberry). J. Food Sci. Technol. 52 (12), 7861–7871. 10.1007/s13197-015-1877-1 26604358 PMC4648887

[B60] Grover S. Tewari S. Sharma R. K. Singh G. Yadav A. Naula S. C. (2016). Effect of subgingivally delivered 10% emblica officinalis gel as an adjunct to scaling and root planing in the treatment of chronic periodontitis - a randomized placebo‐controlled clinical trial. Phytother. Res. 30 (6), 956–962. 10.1002/ptr.5600 26914986

[B61] Guan Q. Feng D. P. Duan B. Z. Fan M. (2022). Research progress on chemical constituents and pharmacological effects of Phyllanthus emblica and predictive analysis of quality markers. Zhong Cao Yao, 1–14.

[B62] Guo J. M. Wang J. K. Tian Y. H. Zhou L. H. (2013). Determination of gallic acid and quercetin content in Euphorbia grandis from different origins. Guizhou Agric. Sci. (05), 61–63.

[B63] Guo X. J. (2013). Chemical and bioactive studies on two species of traditional Chinese medicine. Shandong University. 10.7666/d.Y2331105

[B64] Guo Z. Y. Huang Y. X. Wang G. Q. (2014). Advances in research of emblica fruit( Phyllanthus emblica L. ) for the prevention and treatment of diabetes. Mellitus Complicat. Strait Pharm. 000 (012), 1–4. 10.3969/j.issn.1006-3765.2014.12.001

[B65] Gupta P. Nain P. Sidana J. (2012). Antimicrobial and antioxidant activity ON emblica officinalis seed extracts. Int. J. Res. Ayurveda Pharm. 3 (4), 591–596.

[B66] Hamada S. Kataoka T. Woo J. T. Yamada A. Yoshida T. Nishimura T. (1997). Immunosuppressive effects of gallic acid and chebulagic acid on CTL-mediated cytotoxicity. Biol. Pharm. Bull. 20 (9), 1017–1019. doi:10.1248/bpb.20.1017. 9331989 10.1248/bpb.20.1017

[B67] Hazra B. Sarkar R. Biswas S. Mandal N. (2010). Comparative study of the antioxidant and reactive oxygen species scavenging properties in the extracts of the fruits of Terminalia chebula, Terminalia belerica and Emblica officinalis. BMC Complement. Altern. Med. 10, 20. 10.1186/1472-6882-10-20 20462461 PMC2887379

[B68] Higby G. King N. M. (2013). in The dispensatory of the United States of America. 2nd edition. 25134372

[B69] Hoesel B. Schmid J. A. (2013). The complexity of NF-κB signaling in inflammation and cancer. Mol. Cancer 12 (1), 86–15. 10.1186/1476-4598-12-86 23915189 PMC3750319

[B70] Hou H. Y. (2006). Studies on anti-HBV principles of Phyllanthus emblicaL. Chinese People's Liberation Army Academy of Military Medical Sciences.

[B71] Hou K. W. (2002). The compositions and application of phylanthus emblica in the traditional medicine. Chin. J. Ethn. Folk Med. 11 (6), 345–348.

[B72] Huang C. Z. Tung Y. T. Hsia S. M. Wu C. H. Yen G. C. (2017). The hepatoprotective effect of Phyllanthus emblica L. fruit on high fat diet-induced non-alcoholic fatty liver disease (NAFLD) in SD rats. Food Funct. 8 (2), 842–850. 10.1039/c6fo01585a 28128372

[B73] Huang H. Z. Wei L. X. Lin J. Z. Tan P. Fan S. H. Han Li. (2019). Discussion on the rationality of tannin transformation and Pharmacopoeia determination method in the Reflux process of Phyllanthus emblica. Chin. Pharm. J. 54 (7), 581–587.

[B75] Jaijoy K. Soonthornchareonnon N. Lertprasertsuke N. Panthong A. Sireeratawong S. (2010). Acute and chronic oral toxicity of standardized water extracts from the fruit of Phyllanthus emblica Linn. Int. J. Appl. Res. Nat. Prod. 3 (1), 48–58.

[B76] Jain I. Jain P. Bisht D. Sharma A. Srivastava B. Gupta N. (2015). Use of traditional Indian plants in the inhibition of caries-causing bacteria--Streptococcus mutans. Braz. Dent. J. 26 (2), 110–115. 10.1590/0103-6440201300102 25831099

[B77] Ji X. H. (2010). The screening and mechanism study of Phyllanthus urinaria L. which have anti-angiogenic effect. Nanjing University of Traditional Chinese Medicine.

[B78] Jose J. K. Kuttan R. Jjoc B. (1995). Antioxidant activity of Emblica officinalis. J. Clin. Biochem. Nutr. 19 (2), 63–70. 10.3164/jcbn.19.63

[B79] Kang W. J. Zhang G. M. Zhao X. H. (2011). Hypoglycemic effects of different solvent extractss from phmllanthi Fructus. AnHui Agric. Sci. 39 (30), 18545–18547. 10.3969/j.issn.0517-6611.2011.30.062

[B80] Kapoor L. (1989). CRC handbook of ayurvedic medicinal plants. CRC Press, 175–176.

[B81] Kong X. J. Yu R. Liu J. X. (2016). The influence of flavone extracts of Phyllanthus emblica on influenza H1N1 virus infected pneumonia mice. Her. Traditional Chin. Med. 255 (05), 64–65.

[B82] Krupanidhi S. Peele K. A. Venkateswarulu T. C. Ayyagari V. S. Aishwarya G. John Babu D. (2020). Screening of phytochemical compounds of tinospora cordifolia for their inhibitory activity on sars-cov-2: An *in silico* study. J. Biomol. Struct. Dyn. 39 (15), 5799–5803. 10.1080/07391102.2020.1787226 32627715 PMC7441789

[B83] Kumar A. Tantry B. A. Rahiman S. Gupta U. (2011a). Comparative study of antimicrobial activity and phytochemical analysis of methanolic and aqueous extracts of the fruit of Emblica officinalis against pathogenic bacteria. J. Tradit. Chin. Med. 31 (03), 246–250. 10.1016/j.funbio.2014.07.002 21977871

[B84] Kumar C. P. Swathi R. S. Kumar D. Y. Neelima K. (2014). Effect of emblica officinalis on stress induced biochemical and psychological changes in mice.

[B85] Kumar D. Guna R. Abbirami E. Preethi J. Sivasudha T. Thilagar S. (2020). Cissus quadrangularis (veldt grape) attenuates disease progression and anatomical changes in mono sodium iodoacetate (mia)-induced knee osteoarthritis in the rat model. Food Funct. 11 (9), 7842–7855. 10.1039/d0fo00992j 32812575

[B86] Kumar K. S. Bhowmik D. Dutta A. Yadav A. P. Paswan S. Srivastava S. (2012). Recent trends in potential traditional Indian herbs Emblica officinalis and its medicinal importance. J Pharmacogno Phytochem 1 (1), 18–28.

[B87] Kumar N. Gangappa D. Gupta G. Karnati R. (2014). Chebulagic acid from Terminalia chebula causes G1 arrest, inhibits NFκB and induces apoptosis in retinoblastoma cells. BMC Complement. Altern. Med. 14, 319. 10.1186/1472-6882-14-319 25169718 PMC4158129

[B88] Kumar P. A. Reddy P. Y. Suryanarayana P. Reddy G. (2011b). Effect of the tannoid enriched fraction of Emblica officinalis on α-crystallin chaperone activity under hyperglycemic conditions in lens organ culture.

[B89] Kumar V. Aneesh K. A. Kshemada K. Ajith K. G. S. Binil R. S. S. Deora N. (2017). Amalaki rasayana, a traditional Indian drug enhances cardiac mitochondrial and contractile functions and improves cardiac function in rats with hypertrophy. Sci. Rep. 7 (1), 8588. 10.1038/s41598-017-09225-x 28819266 PMC5561106

[B90] Kumaran A. Karunakaran R. J. (2006). Nitric oxide radical scavenging active components from Phyllanthus emblica L. Plant Foods Hum. Nutr. 61 (1), 1–5. 10.1007/s11130-006-0001-0 16688481

[B91] Lampronti I. Khan M. T. H. Borgatti M. Bianchi N. Gambari R. (2008). Inhibitory effects of Bangladeshi medicinal plant extracts on interactions between transcription factors and target DNA sequences. Evid. Based. Complement. Altern. Med. 5 (3), 303–312. 10.1093/ecam/nem042 PMC2529391 18830455

[B92] Latha R. C. R. Daisy P. (2011). Insulin-secretagogue, antihyperlipidemic and other protective effects of gallic acid isolated from Terminalia bellerica Roxb. in streptozotocin-induced diabetic rats. Chem. Biol. Interact. 189 (1-2), 112–118. 10.1016/j.cbi.2010.11.005 21078310

[B93] Lee J. C. Tsai C. Y. Kao J. Y. Kao M. C. Tsai S. C. Chang C. S. (2008). Geraniin-mediated apoptosis by cleavage of focal adhesion kinase through up-regulation of Fas ligand expression in human melanoma cells. Mol. Nutr. Food Res. 52 (6), 655–663. 10.1002/mnfr.200700381 18435487

[B94] Li B. Huang G. Q. Lu R. M. Wei J. h. Zhong Z. g. (2015). Study on chemical composition of Phyllanthus emblica. J. Chin. Med. Mater. 38 (2), 290–293. 10.13863/j.issn1001-4454.2015.02.021 26415402

[B250] Li C. L. (2001). Pharmacological studies on Phyllanthus emblica. Adv. Pharmacol. 25 (4), 4.

[B95] Li C. L. Chen S. T. Chen W. W. (2002). Determination of vc and gallic acid in amolo capsule by HPLC. Chin. Pharm. Stand. 3 (3), 23–26. 10.3969/j.issn.1009-3656.2002.03.013

[B96] Li J. Wang S. Yin J. Pan L. (2013). Geraniin induces apoptotic cell death in human lung adenocarcinoma A549 cells *in vitro* and *in vivo* . Can. J. Physiol. Pharmacol. 91 (12), 1016–1024. 10.1139/cjpp-2013-0140 24289071

[B97] Li Q. F. Li Y. F. Zhang J. F. Wang K. X. (2020). Methyl galate inhibits Akt and ERK1/2 activity in glioma cells and exerts antitumour effects. J. Ningxia Med. Univ. 42 (08), 757–764. 10.16050/j.cnki.issn1674-6309.2020.08.001

[B98] Li Q. Pei H. H. Li J. Luo Y. H. (2018). The contents of 5 components in Phyllanthus emblica were determined by HPLC, and the principal components and cluster analysis were performed. Chin. Pharm. (11), 1491–1495. 10.6039/j.issn.1001-0408.2018.11.13

[B99] Li Q. Sun P. Li J. Pei H. H. (2019). Effect of salt roasting on the content of gallic acid, ellagic acid and epicatechin in Guangxi Yu Gan Zi. Shi-Zhen Guomao Guomao 30 (9), 2155–2158.

[B100] Li W. Zhang X. Chen R. Li Y. Miao J. Liu G. (2020). HPLC fingerprint analysis of Phyllanthus emblica ethanol extract and their antioxidant and anti-inflammatory properties. J. Ethnopharmacol. 254, 112740. 10.1016/j.jep.2020.112740 32151757

[B101] Li Z. W. Ma S. B. Zeng Q. Y. (2010). Extractsion of Phyllanthus emblica flavone and its antioxidant effect. Jiangsu Agric. Sci. 000 (002), 287–289. 10.3969/j.issn.1002-1302.2010.02.118

[B102] Liang C. Y. Zeng H. S. Tong X. L. Zhang T. Qiu Q. Zhong Y. N. (2010). Study on the chemical constituents of the cotyledon of Phyllanthus emblica in Guangxi with petroleum ether. Lishizhen Tradit. Chin. Med. 21 (10), 2584–2585. 10.3969/j.issn.1008-0805.2010.10.074

[B103] Liang C. Z. Zhang X. Li H. Tao Y. Q. Tao L. J. Yang Z. R. (2012). Gallic acid induces the apoptosis of human osteosarcoma cells *in vitro* and *in vivo* via the regulation of mitogen-activated protein kinase pathways. Cancer biother. Radiopharm. 27 (10), 701–710. 10.1089/cbr.2012.1245 22849560 PMC3516425

[B104] Lim D. W. Kim J. G. Kim Y. T. (2016). Analgesic effect of Indian gooseberry (emblica officinalis fruit) extracts on postoperative and neuropathic pain in rats. Nutrients 8 (12), 760. 10.3390/nu8120760 PMC5188415 27898027

[B105] Linda D. B. Mary F. (2014). Expanded colesevelam administration options with oral suspension formulation for patients with diabetes and hypercholesterolemia. Postgrad. Med. 126 (3), 126–134. 10.3810/pgm.2014.05.2762 24918798

[B106] Liu B. k. Lin Z. Y. Yao L. J. (2015a). Analysis of nutritions and functional compositions in Phyllanthus emblica L. Hans J. Foods snutrition Sci. 04 (2), 59–64. 10.12677/hjfns.2015.42008

[B107] Liu C. Chan L. Liang C. (2019). The anti-aging activities against oxidative damages of <i&gt;Rosa roxburghii&lt;/i&gt; and multi-fruit concentrate drink. J. Food Nutr. Res. (Newark). 7, 845–850. 10.12691/jfnr-7-12-5

[B108] Liu R. Li J. K. Cheng Y. J. Huo T. B. Xue J. Y. Liu Y. L. (2015b). Effects of ellagic acid-rich extracts of pomegranates peel on regulation of cholesterol metabolism and its molecular mechanism in hamsters. Food Funct. 6 (3), 780–787. 10.1039/c4fo00759j 25579987

[B109] Liu W. Xi L. M. Wang J. L. Wan C. P. (2017). Structural identification of phenolic constituents from phyllanthus emblica l. and their inhibitory activity on α-glucosidase. Mod. Food Sci. Technol. 33 (12), 50–55. 10.13982/j.mfst.1673-9078.2017.12.008

[B110] Liu X. Cui C. Zhao M. Wang J. Luo W. Bao Y. (2008). Identification of phenolics in the fruit of emblica (phyllanthus emblica l.) and their antioxidant activities. Food Chem. 109 (4), 909–915. 10.1016/j.foodchem.2008.01.071 26050007

[B111] Liu X. L. Zhao M. M. Cui C. J. Luo W. Zhao Q. Z. (2007b). A study of the composition of essential oils from emblica (Phyllanthus emblica L.) fruit by supercritical fluid extractsion and their antioxidant activity. 10.9774/GLEAF.978-1-909493-38-4-2

[B113] Liu X. L. Zhao M. M. Yang B. (2007a). Antioxidant activities and functional composition content of Phyllanthus emblica L. Fruits. Nat. Prod. Res. Dev. 19 (2). 10.3969/j.issn.1001-6880.2007.02.002

[B115] Liu Z. Y. Wang D. C. Chen Y. W. (2002). Experiment studies on the pharmacodynamics experiment by corilagin. Cancer Res. Prev. Treat. 29 (5), 356–358. 10.3971/j.issn.1000-8578.2002.05.003

[B116] Lu k. Iwenofu H. O. Mitra R. Mo X. K. Dasgupta P. X. Basu S. (2020). Chebulinic acid is a safe and effective antiangiogenic agent in collagen-induced arthritis in mice. 10.1186/s13075-020-02370-1 PMC7682078 33225986

[B117] Luo C. L. (2010). Immunoregulative action and inhibitive effect of Phyllanthus emblica on tumour cells. Chin. J. Exp. Traditional Med. Formulae 16 (13), 155–158. 10.3969/j.issn.1005-9903.2010.13.047

[B118] Luo D. S. (2004). Newly repaired jingzhu materia medica. Sichuan Science and Technology Press.

[B120] Luo W. Wen L. R. Zhao M. M. Yang B. Ren J. Shen G. (2012). Structural identification of isomallotusinin and other phenolics in Phyllanthus emblica L. fruit hull. Food Chem. 132 (3), 1527–1533. 10.1016/j.foodchem.2011.11.146 29243645

[B121] Mahata S. Pandey A. Shukla S. Tyagi A. Husain S. A. Das B. C. (2013). Anticancer activity of Phyllanthus emblica linn. (Indian gooseberry): Inhibition of transcription factor AP-1 and HPV gene expression in cervical cancer cells. Nutr. Cancer 65, 88–97. 10.1080/01635581.2013.785008 23682787

[B122] Majeed M. Bhat B. Jadhav A. N. Srivastava J. S. Nagabhushanam K. (2009). Ascorbic acid and tannins from Emblica officinalis Gaertn. Fruits--a revisit. J. Agric. Food Chem. 57 (1), 220–225. 10.1021/jf802900b 19063633

[B123] Majeed M. Majeed S. Mundkur L. Nagabhushanam K. Ali F. Beede K. (2019). Standardized Emblica officinalis fruit extract inhibited the activities of α-amylase, α-glucosidase, and dipeptidyl peptidase-4 and displayed antioxidant potential. J. Sci. Food Agric. 100 (2), 509–516. 10.1002/jsfa.10020 31487036 PMC6973029

[B124] Malar H. Mettilda S. M. (2009). Hepato-protective activity of Phyllanthus emblica against paracetamol induced hepatic damage in wister albino rats.

[B125] Malve H. O. Raut S. B. Marathe P. A. Rege N. N. (2014). Effect of combination of Phyllanthus emblica, Tinospora cordifolia, and Ocimum sanctum on spatial learning and memory in rats. J. Ayurveda Integr. Med. 5 (4), 209–215. 10.4103/0975-9476.146564 25624694 PMC4296432

[B126] Mao S. N. (2019). Study on the establishment conditions and quality characteristics of Euphorbia. Chengdu University of Traditional Chinese Medicine. 10.26988/d.cnki.gcdzu.2019.000133

[B127] Mathew M. Subramanian S. (2014). *In vitro* screening for anti-cholinesterase and antioxidant activity of methanolic extracts of ayurvedic medicinal plants used for cognitive disorders. PLoS One 9 (1), e86804. 10.1371/journal.pone.0086804 24466247 PMC3900633

[B128] McCord J, M. (1985). Oxygen-derived free radicals in postischemic tissue injury. N. Engl. J. Med. 312 (3), 159–163. 10.1056/NEJM198501173120305 2981404

[B129] Mehmood M. H. Rehman A. Rehman N. U. Gilani A. H. (2013). Studies on prokinetic, laxative and spasmodic activities of Phyllanthus emblica in experimental animals. Phytother. Res. 27 (7), 1054–1060. 10.1002/ptr.4821 22972571

[B130] Meng Z. Y. Zhu Y. L. Wang G. W. (2011). Extractsion process and composition analysis of the kernel oil of Eugenia caryophyllata. Chem. industry For. Prod. (8), 60–61. 10.13456/j.cnki.lykt.2011.08.004

[B131] Ming Y. Q. Wang S. M. (2018). The brewing method of Phyllanthus emblica healthy ginger wine.

[B132] Murugesan S. Kottekad S. Crasta I. Sreevathsan S. Usharani D. Perumal M. K. (2021). Targeting COVID-19 (SARS-CoV-2) main protease through active phytocompounds of ayurvedic medicinal plants – emblica officinalis (Amla), Phyllanthus niruri Linn. (Bhumi Amla) and Tinospora cordifolia (Giloy) – a molecular docking and simulation study. Comput. Biol. Med. 136, 104683. 10.1016/j.compbiomed.2021.104683 34329860 PMC8302490

[B133] Muthuraman A. Sood S. Singla S. K. (2011). The antiinflammatory potential of phenolic compounds from Emblica officinalis L. in rat. Inflammopharmacology 19 (6), 327–334. 10.1007/s10787-010-0041-9 20596897 PMC3227803

[B134] Na Y. Yang F. R. (2002). Overview of research on development and utilization of medicinal plant resources in foreign countries. For. Investigation Des. (03), 70.

[B135] Natesh J. Mondal P. Penta D. Ajees A. Meeran S. M. (2021). Culinary spice bioactives as potential therapeutics against sars-cov-2: Computational investigation. Comput. Biol. Med. 128, 104102. 10.1016/j.compbiomed.2020.104102 33190011 PMC7606080

[B136] Ngamkitidechakul C. Jaijoy K. Hansakul P. Soonthornchareonnon N. Sireeratawong S. (2010). Antitumour effects of Phyllanthus emblica L.: Induction of cancer cell apoptosis and inhibition of *in vivo* tumour promotion and *in vitro* invasion of human cancer cells. Phytother. Res. 24 (9), 1405–1413. 10.1002/ptr.3127 20812284

[B137] Nicolis E. Lampronti I. Dechecchi M. C. Borgatti M. Tamanini A. Bianchi N. (2008). Pyrogallol, an active compound from the medicinal plant Emblica officinalis, regulates expression of pro-inflammatory genes in bronchial epithelial cells. Int. Immunopharmacol. 8 (12), 1672–1680. 10.1016/j.intimp.2008.08.001 18760383

[B138] Nisar M. F. He J. W. Ahmed A. Yang Y. X. Li M. X. Wang C. P. (2018). Chemical components and biological activities of the genus Phyllanthus: A review of the recent literature. Molecules 23 (10), 2567. 10.3390/molecules23102567 PMC6222918 30297661

[B139] Olennikov D. N. Kashchenko N. I. Schwabl H. Vennos C. Loepfe C. (2015). New mucic acid gallates from Phyllanthus emblica. Chem. Nat. Compd. 51 (4), 666–670. 10.1007/s10600-015-1380-y

[B140] Packirisamy R. M. Bobby Z. Panneerselvam S. Koshy S. M. Jacob S. E. (2018). Metabolomic analysis and antioxidant effect of amla (emblica officinalis) extract in preventing oxidative stress-induced red cell damage and plasma protein alterations: An *in vitro* study. J. Med. Food 21 (1), 81–89. 10.1089/jmf.2017.3942 29064307

[B141] Pareek S. Shikov A. N. Pozharitskaya O. N. Makarov V. G. González-Aguilar G. A. Ramalho S. A. (2018). Indian gooseberry (emblica officinalis gaertn.). General & Introd. Food Sci. Technol. 1077, 1077–1106. 10.1002/9781119158042.ch54

[B142] Patel S. S. Goyal R. K. (2011). Prevention of diabetes-induced myocardial dysfunction in rats using the juice of the Emblica officinalis fruit. Exp. Clin. Cardiol. 16 (3), 87–91. 22065939 PMC3209545

[B143] Poltanov E. A. Shikov A. N. Dorman H. Pozharitskaya O. N. Makarov V. G. Tikhonov V. P. (2010). Chemical and antioxidant evaluation of indian gooseberry (emblica officinalis gaertn. syn. phyllanthus emblica l.) supplements. Phytother. Res. 23 (9), 1309–1315. 10.1002/ptr.2775 19172666

[B144] Qiu F. H. Liu L. F. Lin y. Yang Z. T. Qiu F. (2019). Corilagin inhibits esophageal squamous cell carcinoma by inducing DNA damage and down-regulation of RNF8. Anticancer. Agents Med. Chem. 19 (8), 1021–1028. 10.2174/1871520619666190307120811 30848215

[B145] Qu C. Lai Z. C. Pei Y. (2010). Study on the anti-HSV activity of crude extracts from phyllanthu semblica *in vitro* . Lishizhen Med. Materia Medica 21 (004), 784–786. 10.3969/j.issn.1008-0805.2010.04.006

[B146] Rahman S. Akbor M. M. Howlader A. Jabbar A. (2009). Antimicrobial and cytotoxic activity of the alkaloids of Amlaki (Emblica officinalis). Pak. J. Biol. Sci. 12 (16), 1152–1155. 10.3923/pjbs.2009.1152.1155 19899327

[B147] Rai N. Tiwari L. Sharma R. K. Verma A. K. (2012). Pharmaco-botanical profile on Emblica officinalis Gaertn–a pharmacopoeial herbal drug STM J, 29–41.

[B148] Ram S, M. Neetu D. Yogesh B. Anju B. Dipti P. Pauline T. (2002). Cyto-protective and immunomodulating properties of Amla (Emblica officinalis) on lymphocytes:an *in-vitro* study. J. Ethnopharmacol. 81 (1), 5–10. 10.1016/S0378-8741(01)00421-4 12020921

[B149] Ramakrishna V. Gupta K. P. Setty H. O. Kondapi K. A. Medicine A. (2014). Neuroprotective effect of Emblica Officinalis extracts against H2O2 induced DNA damage and repair in neuroblastoma cells.

[B150] Ramanujam K. Sundrarajan M. (2014). Antibacterial effects of biosynthesized MgO nanoparticles using ethanolic fruit extract of Emblica officinalis. J. Photochem. Photobiol. B 141, 296–300. 10.1016/j.jphotobiol.2014.09.011 25463681

[B151] Ramesh P. S. Kokila T. Geetha D. (2015). Plant mediated green synthesis and antibacterial activity of silver nanoparticles using Emblica officinalis fruit extract. Spectrochim. Acta. A Mol. Biomol. Spectrosc. 142, 339–343. 10.1016/j.saa.2015.01.062 25710891

[B152] Romano M. Vitaglione P. Sellitto S. D’Argenio G. (2014). Addendum: Nutraceuticals for protection and healing of gastrointestinal mucosa. Romano M, vitaglione P, sellitto S, D'argenio G. Curr med chem 2012, 19: 109-117. Curr. Med. Chem. 21 (28), 3310. 10.2174/092986732128140825173622 25181480

[B153] Rosarin F. S. Arulmozhi V. Nagarajan S. Mirunalini S. (2013). Antiproliferative effect of silver nanoparticles synthesized using amla on Hep2 cell line. Asian pac. J. Trop. Med. 6 (1), 1–10. 10.1016/S1995-7645(12)60193-X 23317879

[B154] Saeed S. Tariq P. (2007). Antibacterial activities of Emblica officinalis and Coriandrum sativum against Gram negative urinary pathogens. Pak. J. Pharm. Sci. 20 (1), 32–35. 10.1016/j.neuro.2006.10.004 17337425

[B155] Sai P. Ratna M. Upadhyaya B. N. Appaji R. R. (2010). A clinical study on the use of puskara mooladi choorna in tamaka shvasa (bronchial asthma) with pulmonary function tests. Anc. Sci. Life 29 (3), 1–5. PMC3336280 22557352

[B156] Saito K. Kohno M. Yoshizaki F. Niwano Y. (2008). Extensive screening for edible herbal extracts with potent scavenging activity against superoxide anions. Plant Foods Hum. Nutr. 63 (2), 65–70. 10.1007/s11130-008-0071-2 18236159

[B157] Sancheti G. Jindal A. Kumari R. Goyal P. K. (2005). Chemopreventive action of emblica officinalis on skin carcinogenesis in mice. Asian pac. J. Cancer Prev. 6 (2), 197–201. 16101333

[B158] Sangeetha N. Mercy S. Kavitha M. Selvaraj D. Sathishkumar R. Ganesh D. (2010). Morphological variation in the Indian gooseberries (Phyllanthus emblica and Phyllanthus indofischeri) and the chloroplast trnL (UAA) intron as candidate gene for their identifi cation. Plant Genet. Resour. 8, 191–197. 10.1017/S1479262110000171

[B159] Saxena A. Kaur K. Hegde S. Kalekhan F. M. Fayad R. (2014). Dietary agents and phytochemicals in the prevention and treatment of experimental ulcerative colitis. J. Tradit. Complement. Med. 4 (4), 203–217. 10.4103/2225-4110.139111 25379461 PMC4220497

[B160] Scartezzini P. Antognoni F. Raggi M. A. Poli F. Sabbioni C. (2006). Vitamin C content and antioxidant activity of the fruit and of the Ayurvedic preparation of Emblica officinalis Gaertn. J. Ethnopharmacol. 104 (1-2), 113–118. 10.1016/j.jep.2005.08.065 16226416

[B161] Scartezzini P. Speroni E. (2000). Review on some plants of Indian traditional medicine with antioxidant activity. J. Ethnopharmacol. 71 (1-2), 23–43. 10.1016/s0378-8741(00)00213-0 10904144

[B162] Shanmugarajan D. Girish C. Harivenkatesh N. Chanaveerappa B. Lakshmi N. (2021). Antihypertensive and pleiotropic effects of Phyllanthus emblica extract as an add-on therapy in patients with essential hypertension-A randomized double-blind placebo-controlled trial. Phytother. Res. 35 (6), 3275–3285. 10.1002/ptr.7043 33570228

[B163] Shao Y. Y. Zhu D. Yang G. H. Mao X. Xia Q. (2014). Health effects of tannin-containing herbs Chinese society of traditional Chinese medicine.

[B164] Shi Y. P. Yang Z. M. (1994). The study of nutritive componets of flash of Phyllanthus emblica Fruit in GuangZhou. J. Guizhou Normal Univ. (2), 1–6.

[B166] Shynu M. P Gupta K. Saini M. (2011). Antineoplastic potential of medicinal plants. Recent Pat. Biotechnol. 5 (2), 85–94. 10.2174/187220811796365662 21707529

[B167] Siddiqui A. J. Danciu C. Ashraf S. A. Moin A. Adnan M. Alreshidi M. (2020). Plants-derived biomolecules as potent antiviral phytomedicines: New insights on ethnobotanical evidences against coronaviruses. Plants (Basel). 9 (9), 1244. 10.3390/plants9091244 PMC7570315 32967179

[B168] Sidhu S. Pandhi P. Malhotra S. Vaiphei K. Khanduja K. L. (2011). Beneficial effects of Emblica officinalis in L-arginine-induced acute pancreatitis in rats. J. Med. Food 14 (1-2), 147–155. 10.1089/jmf.2010.1108 21138365

[B169] Singh M. K. Dwivedi S. Yadav S. S. Sharma P. Khattri S. (2014). Arsenic-induced hepatic toxicity and its attenuation by fruit extract of emblica officinalis (amla) in mice. Indian J. Clin. biochem. 29 (1), 29–37. 10.1007/s12291-013-0353-9 24478546 PMC3903921

[B170] Singh M. K. Yadav S. S. Gupta V. Khattri S. (2013). Immunomodulatory role of Emblica officinalis in arsenic induced oxidative damage and apoptosis in thymocytes of mice. BMC Complement. Altern. Med. 13, 193. 10.1186/1472-6882-13-193 23889914 PMC3733846

[B171] Sultana S. Ahmed S. Jahangir T. (2008). Emblica officinalis and hepatocarcinogenesis: A chemopreventive study in wistar rats. J. Ethnopharmacol. 118 (1), 1–6. 10.1016/j.jep.2007.04.021 18467048

[B172] Sumantran V. N. Joshi A. K. Boddul S. Koppikar S. J. Warude D. Patwardhan B. (2011). Antiarthritic activity of a standardized, multiherbal, ayurvedic formulation containing boswellia serrata: *In vitro* studies on knee cartilage from osteoarthritis patients. Phytother. Res. 25 (9), 1375–1380. 10.1002/ptr.3365 25363757

[B173] Sumitra M. Manikandan P. Gayathri V. S. Mahendran P. Suguna L. (2009). Emblica officinalis exerts wound healing action through up-regulation of collagen and extracellular signal-regulated kinases (ERK1/2). Wound Repair Regen. 17 (1), 99–107. 10.1111/j.1524-475X.2008.00446.x 19152656

[B174] Sun X. F. Wang W. Du G. Y. Lu W. b. (2002). [Study on anti-HIV drugs in Egyptian medicinal plants]. China J. Chin. Materia Medica 27 (9), 649–679. 10.3321/j.issn:1001-5302.2002.09.003 12866564

[B176] Suresh K. Vasudevan D. M. (1994). Augmentation of murine natural killer cell and antibody dependent cellular cytotoxicity activities by Phyllanthus emblica, a new immunomodulator. J. Ethnopharmacol. 44 (1), 55–60. 10.1016/0378-8741(94)90099-x 7990505

[B177] Suryanarayana P. Kumar P. A. Saraswat M. Petrash J. M. Reddy G. B. (2004). Inhibition of aldose reductase by tannoid principles of emblica officinalis: Implications for the prevention of sugar cataract. Mol. Vis. 10, 148–154. 15031705

[B248] Tan G. S. Jiang R. F. Li Y. P. Li F. A. Zhang D. J. (2015). Protective effect of Wu Wei Yu Gan Zi San on myocardial ischemia-reperfusion injury in rats. West China J. Pharm. 30 (2), 3. 10.13375/j.cnki.wcjps.2015.02.024

[B178] Tasanarong A. Kongkham S. Itharat A. (2014). Antioxidant effect of Phyllanthus emblica extract prevents contrast-induced acute kidney injury. BMC Complement. Altern. Med. 14 (1), 138. 10.1186/1472-6882-14-138 24755233 PMC4045981

[B179] Thakur R. S. Puri H. S. Husain A. (1993). Major medicinal plants of India. Phytochemistry 33 (3), 740. 10.1016/0031-9422(93)85498-G

[B180] Thenmozhi A. J. Dhivyabharathi M. Raja T. R. W. Manivasagam T. Essa M. M. (2016). Tannoid principles of Emblica officinalis renovate cognitive deficits and attenuate amyloid pathologies against aluminum chloride induced rat model of Alzheimer's disease. Nutr. Neurosci. 19 (6), 269–278. 10.1179/1476830515Y.0000000016 25842984

[B181] Thirunavukkarasu M. Selvaraju V. Tapias L. Sanchez J. A. Palesty J. A. Maulik N. (2015). Protective effects of Phyllanthus emblica against myocardial ischemia-reperfusion injury: The role of PI3-kinase/glycogen synthase kinase 3β/β-catenin pathway. J. Physiol. Biochem. 71 (4), 623–633. 10.1007/s13105-015-0426-8 26342597

[B182] Tiwari V. Kuhad A. Chopra K. (2011). Emblica officinalis corrects functional, biochemical and molecular deficits in experimental diabetic neuropathy by targeting the oxido-nitrosative stress mediated inflammatory cascade. Phytother. Res. 25 (10), 1527–1536. 10.1002/ptr.3440 21394805

[B183] Uddin M. S. Mamun A. A. Hossain M. S. Akter F. Iqbal M. A. Asaduzzaman M. (2016). Exploring the effect of Phyllanthus emblica L. On cognitive performance, brain antioxidant markers and acetylcholinesterase activity in rats: Promising natural gift for the mitigation of alzheimer's disease. Ann. Neurosci. 23 (4), 218–229. 10.1159/000449482 27780989 PMC5075744

[B184] Udupa K. N. (1985). Ayurveda for promotion of health. J. Ayurveda 3.

[B185] Upadya H. Prabhu S. Prasad A. Subramanian D. Gupta S. Goel A. (2019). A randomized, double blind, placebo controlled, multicenter clinical trial to assess the efficacy and safety of Emblica officinalis extract in patients with dyslipidemia. BMC Complement. Altern. Med. 19 (1), 27. 10.1186/s12906-019-2430-y 30670010 PMC6341673

[B186] Villacorta L. Azzi A. Zingg J. M. (2007). Regulatory role of vitamins E and C on extracellular matrix components of the vascular system. Mol. Asp. Med. 28 (5-6), 507–537. 10.1016/j.mam.2007.05.002 17624419

[B187] Vormisto A. I. Summanen J. Kankaanranta. H. Vuorela H. Asmawi Z. M. Moilanen E. (1997). Anti-inflammatory activity of extractss lirom leaves of Phyllanthus emblica. Planta Med. 63 (6), 518–524. 10.1055/s-2006-957754 9434603

[B188] Wang C. C. Yuan J. R. Wang C. F. Yang N. Chen J. Liu D. (2017). Anti-inflammatory effects of Phyllanthus emblica L on benzopyrene-induced precancerous lung lesion by regulating the IL-1β/miR-101/lin28B signaling pathway. Integr. Cancer Ther. 16 (4), 505–515. 10.1177/1534735416659358 27562754 PMC5739133

[B189] Wang H. M. Fu L. Cheng C. C. Gao R. Lin M. Y. Su H. L. (2019). Inhibition of LPS-induced oxidative damages and potential anti-inflammatory effects of Phyllanthus emblica extract via down-regulating NF-κB, COX-2, and iNOS in RAW 264.7 cells. Antioxidants (Basel) 8 (8), 270. 10.3390/antiox8080270 PMC6721275 31382466

[B190] Wang H. Z. Shan M. Sun J. Y. (2007). Progress of pharmacological research on alpha-linolenic acid.

[B191] Wang J. Liu Y. T. Xiao L. Zhu L. Wang Q. Yan T. (2014). Anti-inflammatory effects of apigenin in lipopolysaccharide-induced inflammatory in acute lung injury by suppressing COX-2 and NF-kB pathway. Inflammation 7 (6), 2085–2090. 10.1007/s10753-014-9942-x 24958013

[B192] Wang L. P. Cui Y. X. Xu J. (2018). Anti-aging effect of Phyllanthus emblical. On *Caenorhabditis elegans* . J. Jilin Univ. 56 (03), 276–280. 10.13413/j.cnki.jdxblxb.2018.03.45

[B193] Wang M. Sun P. Y. Liang W. Dai Y. Y. Cai S. (2020). Critical success factors for the entry of compounded Chinese herbal medicines into the European market. Chin. J. Pharmacol. Toxicol. 34 (2), 81–94. 10.3867/j.issn.1000-3002.2020.02.001

[B194] Wang R. (2017). Inhibition effect on α-glucosidase and antioxidant activity for polyphenol extractss from Phyllanthus emblica L. Food Res. Dev. 38 (11), 13–16. 10.3969/j.issn.1005-6521.2017.11.004

[B195] Wang S. H. Cheng J. T. Guo C. Cui W. J. Shi J. Liu A. (2019). Chemical constituents of phyllanthus emblica and its anti-inflammation activities.

[B196] Wang S. P. Ma Y. Wang S. H. (2009). Analysis of chemical composition of volatile oil of Phyllanthus emblica L.from Sichuanby GC-MS. West China Pharmaceutical Journal, 279–281.

[B197] Wang W, C. (2022). Book review of Chinese medicinal plants. Chin. J. Traditional Chin. Med. 47 (09), 2556.

[B198] Wang Z. X. Xu G. L. Wang Y. F. (2013). The possible role of oxidative stress and antioxidants in the occurrence and development of diabetic complications. J. Med. Res. 042 (004), 12–15.

[B199] Wei L. Zhao M. Bao Y. Ren J. Shen G. Rao G. J. (2011). Antioxidant and antiproliferative capacities of phenolics purified from Phyllanthus emblica L. fruit. Food Chem. x. 126 (1), 277–282. 10.1016/j.foodchem.2010.11.018

[B200] Wei Z. X. Qiu H. X. Zhou L. H. (2002). Studies on pollen morphology of euphorbiaceae. Plant Stud. Yunnan (02), 253–259. 10.3969/j.issn.2095-0845.2002.02.014

[B201] Wen C. L. Yang F. Yao W. H. Guo J. Yan Y. Xie W. (2018). Study on acute toxicity, genotoxicity and subacute toxicity of extractss from Phyllanthus emblica. Prev. Med. Forum (06), 401–403+406. 10.16406/j.pmt.issn.1672-9153.2018.06.001

[B202] Wu L. F. Ye T. Liang L. J. (2017). Research on stability of tannin part in Phyllanthus emblica L. In artificial gastric and intestinal juice. World Sci. Technology-Modernization Traditional Chin. Med. (08), 136–140. 10.11842/wst.2017.08.023

[B203] Wu L. Zhang Q. Liang W. Ma Y. Niu L. Zhang L. (2021). Phytochemical analysis using uplc-msn combined with network pharmacology approaches to explore the biomarkers for the quality control of the anticancer tannin fraction of phyllanthus emblica l. habitat in Nepal. Evid. Based. Complement. Altern. Med. 2021, 6623791. 10.1155/2021/6623791 PMC8018855 33833816

[B204] Wu X. F. Li L. Li Y. Lv H. N. Liu Y. B. Hu Y. C. (2017). Phloroglucinols with antioxidant activities isolated from lysidice rhodostegia. Molecules 22 (6), 855. 10.3390/molecules22060855 PMC6152794 28545244

[B205] Wu X. H. Xie Z. F. Huang Y. F. (2003). The chemical composition and health-protection functions of Phyllanthus emblica L. Wild Plant Resour. China 22 (006), 69–71. 10.3969/j.issn.1006-9690.2003.06.026

[B206] Wu. M. Cai,. J. H. Fang. Z. F. Li S. S. Huang. Z. Q. (2022). The composition and anti-aging activities of polyphenol extracts from Phyllanthus emblica L. Fruit. Nutr. 14, 857. 10.3390/NU14040857 PMC8878974 35215512

[B207] Xia Q. Xiao P. G. Wang L. W. Kong J. (1997). Ethnopharmacology of Phyllanthus emblica L. J. Traditional Chin. Med. 22 (9), 515–518. 11038937

[B208] Xiang Y. Pei Y. Qu C. Lai Z. Ren Z. Yang K. (2011). *In vitro* anti-herpes simplex virus activity of 1, 2, 4, 6-tetraO-galloyl-beta-D-glucose from Phyllanthus emblica L.(Euphorbiaceae). Phytother. Res. 25, 975–982. 10.1002/ptr.3368 21213355

[B209] Xiao P. G. Liu C. X. (1983). Overview of research, production and demand of medicinal plants abroad. Zhong Cao Yao 14 (06), 40–44.

[B210] Xu M. Q. (2007). Nutrition ingredientsand health functions of buccal tablet of Phyllanthus emblical. J. Quanzhou Normal Univ. 25 (002), 62–64. 10.3969/j.issn.1009-8224.2007.02.014

[B211] Xu M. Zhu H. T. Cheng R. R. Dong W. Chong R. Y. Tanaka T. (2016). Antioxidant and hyaluronidase inhibitory activities of diverse phenolics in Phyllanthus emblica. Nat. Prod. Res. 30 (23), 2726–2729. 10.1080/14786419.2015.1137573 26872865

[B212] Xu Y. X. (2009). Study on the chemical composition and total phenol extractsion process of Phyllanthus emblica L. Beijing University of Traditional Chinese Medicine.

[B213] Yang B. Liu P. (2014). Composition and biological activities of hydrolyzable tannins of fruits of Phyllanthus emblica. J. Agric. Food Chem. 62 (3), 529–541. 10.1021/jf404703k 24369850

[B214] Yang C. B. Zhang F. Deng M. C. He G. Y. Yue J. M. Lu R. H. (2007). A new ellagitannin from the fruit of Phyllanthus Emblica L. J. Chin. Chem. Soc. 54 (6), 1615–1618. 10.1002/JCCS.200700228

[B215] Yang F. Xiang H. Q. Ju S. Li Y. J. Zhang C. R. Yang Y. F. (2010). Effects of 1, 2, 4, 6-tetra-o-galloyl-β-d-glucose from p. emblica on hbsag and hbeag secretion in HepG2.2.15 cell culture. Virol. Sin. 25 (5), 375–380. 10.1007/s12250-010-3144-y 20960184 PMC7090425

[B216] Yang F. Yaseen A. Chen B. Li F. Lake H. Hu W. (2020). Chemical constituents from the fruits of Phyllanthus emblica L. Biochem. Syst. Ecol. 92, 104122. 10.1016/j.bse.2020.104122

[B217] Yang N. T. Zhang Y. He L. J. Fan R. Y. Gou W. Wang C. (2018). Ethnobotanical studies on the traditional edible sour plants of the Bai people in the Dali region. J. Plant Resour. Environ. 27 (2), 8. 10.3969/j.issn.1674-7895.2018.02.12

[B218] Yang S. H. Wu C. H. Lu C. C. Yen G. C. (2016). Inhibitory effects of Phyllanthus emblica L. on hepatic steatosis and liver fibrosis *in vitro* . J. Funct. Foods 20, 20–30. 10.1016/j.jff.2015.10.012

[B247] Yang X. Liang R. J. Hong A. H. Wang Y. F. Cen Y. Z. (2014). Chemical constituents in barks of wild Phyllanthus emblica. Zhong Cao Yao (2), 170–174. 10.7501/j.issn.0253-2670.2014.2.005

[B220] Yi Z. C. Wang Z. Li H. X. Liu M. J. Wu R. C. Wang X. H. (2004). Effects of chebulinic acid on differentiation of human leukemia K562 cells. Acta Pharmacol. Sin. 25 (2), 231–238. 14769215

[B221] Yokozawa T. Kim H. Y. Kim H. J. Tanaka T. Juneja L. R. Okubo T. (2007). Amla (emblica officinalis gaertn.) attenuates age-related renal dysfunction by oxidative stress. J. Agric. Food Chem. 55 (19), 7744–7752. 10.1021/jf072105s 17715896

[B222] Yoshimura Y. Nishii S. Zaima N. Moriyama T. Kawamura Y. (2013). Ellagic acid improves hepatic steatosis and serum lipid composition through reduction of serum resistin levels and transcriptional activation of hepatic ppara in obese, diabetic KK-A(y) mice. Biochem. Biophys. Res. Commun. 434 (3), 486–491. 10.1016/j.bbrc.2013.03.100 23583377

[B223] Yu T. Gongbu Y. D. (1987). The four medical classics. Beijing People's Medical Publishing House.

[B224] Yuan J. M. Yang X. Q. Xu Z. P. Kong W. X. Zhao Q. L. Lei B. (2021). Evaluation of amino acid composition and nutritional value of Yu Ganzi fruit in the dry and hot valley region of Yunnan. Jiangxi J. Agric. 33 (10), 9.

[B225] Zeng X. X. Cen Z. F. (2012). Anti-inflammatory and analgesic effects of Phyllanthus emblica extracts. Guangdong Med. 33 (023), 3533–3536. 10.3969/j.issn.1001-9448.2012.23.008

[B226] Zhang G. Zhou S. M. Tian H. J. (2011). An experimental study of Phyllanthus EmblicaL. On the anti-fatigue effect on mice in simulated plateau environment. Pharm. J. Chin. People's Liberation Army (03), 208–211. 10.3969/j.issn.1008-9926.2011.03.07

[B227] Zhang J. Miao D. Zhu W. F. Xu J. Liu W. Y. Kitdamrongtham W. (2017). Biological activities of phenolics from the fruits of phyllanthus emblica L. (euphorbiaceae). Chem. Biodivers. 14 (12), e1700404. 10.1002/cbdv.201700404 28960771

[B228] Zhang L. Z. Zhao W. H. Guo Y. J. Tu G. z. Lin S. Xin L. g. (2003). [Studies on chemical constituents in fruits of Tibetan medicine Phyllanthus emblica]. J. Chin. Materia Medica 28 (10), 940–943. 15620182

[B229] Zhang W. L. Zhang J. Y. Zhang K. L. Huang Y. C. Lin W. B. Liang Z. W. (2019). Determination of gallic acid and ellagic acid in extractss of Phyllanthus emblica by HPLC. China Pharm. Co. 28 (2), 26–29.

[B230] Zhang W. W. Zhang H. Zheng H. Li K. Feng Y. (2013). Microwave-assisted extractsion and fatty acid composition analysis of oil from Phyllanthus emblica L. seeds. Food Sci. 34 (20), 6. 10.7506/spkx1002-6630-201320003

[B231] Zhang X. M. Liu X. F. Gao Y. T. (2011). On the Antioxidation Effects of the extracts of Phyllanthus emblica L. Res. Trace Elem. Health 28 (003), 29–30.

[B232] Zhang Y, J. Tanaka T. Yang C, R. Kouno I. (2001). New phenolic constituents from the fruit juice of Phyllanthus emblica. Chem. Pharm. Bull. 49 (5), 537–540. 10.1248/cpb.49.537 11383602

[B234] Zhang Y. J. Abe T. Tanaka T. Yang C. R. Kouno I. (2002). Two new acylated flavanone glycosides from the leaves and branches of Phyllanthus emblica. Chem. Pharm. Bull. 50 (6), 841–843. 10.1248/cpb.50.841 12045344

[B236] Zhang Y. Zhao L. J. Guo X. J. Li C. Li H. Z. Lou H. X. (2016). Chemical constituents from Phyllanthus emblica and the cytoprotective effects on H_2_O_2_-induced PC12 cell injuries. Arch. Pharm. Res. 39 (9), 1202–1211. 10.1007/s12272-014-0433-2 24993870

[B238] Zhao B. Hu M. (2013). Gallic acid reduces cell viability, proliferation, invasion and angiogenesis in human cervical cancer cells. Oncol. Lett. 6 (6), 1749–1755. 10.3892/ol.2013.1632 24843386 PMC4023842

[B239] Zhao K. H. Wang J. Lv X. M. (2018). Pharmacological study of Tibetan medicine emblica in the prevention and treatment of high altitude polycythemia. J. Traditional Chin. Med. 033 (003), 934–939.

[B240] Zhao M. M. Liu X. L. Cui C. Luo W. (2007). Composition and antimicrobial activity of essential oil from Phyllanthus emblica L. By supercritical CO2 extractsion. J. South China Univ. Technol. 035 (012), 116–120. 10.3321/j.issn:1000-565x.2007.12.023

[B241] Zhao X. W. Liu P. Y. Liu D. Sun S. S. Li Z. Yu K. X. (2015). Progress in the study of the conformational relationships of flavonoids. Zhong Cao Yao 046 (021), 3264–3271.

[B242] Zhong Z. G. Wu D. P. Huang J. L. Liang H. Pan Z. H. Zhang W. Y. (2011). Progallin A isolated from the acetic ether part of the leaves of Phyllanthus emblica L. induces apoptosis of human hepatocellular carcinoma BEL-7404 cells by upregulation of Bax expression and down-regulation of Bcl-2 expression. J. Ethnopharmacol. 133 (2), 765–772. 10.1016/j.jep.2010.11.001 21073944

[B243] Zhou K. Jian P. Liang W. Y. Liang L. J. Cui Y. P. Ye T. (2018). Research progress on the chemical constituents and pharmacological effects of the Tibetan medicine Fructus mongolicum. World Sci. Technol. - Mod. TCM 20 (9), 1608–1614.

[B244] Zhu X. X. Wang J. J. Ou Y. Han W. W. Li H. F. (2013). Polyphenol extract of Phyllanthus emblica (PEEP) induces inhibition of cell proliferation and triggers apoptosis in cervical cancer cells. Eur. J. Med. Res. 18 (1), 46. 10.1186/2047-783X-18-46 24245877 PMC4176749

[B245] Zhu Z. M. Jin J. W. Zhao D. P. (2018). A Chinese medicinal composition capable of boosting immunity, lowering blood lipids, and protecting the heart and brain and its preparation method.

