# Supplementary figures and images for: Current advances on the phytochemical composition, pharmacologic effects, toxicology, and product development of Phyllanthi Fructus
Source: Front Pharmacol. 2022 Oct 19;13:1017268. doi: 10.3389/fphar.2022.1017268 (PMC9626985; doi:10.3389/fphar.2022.1017268)

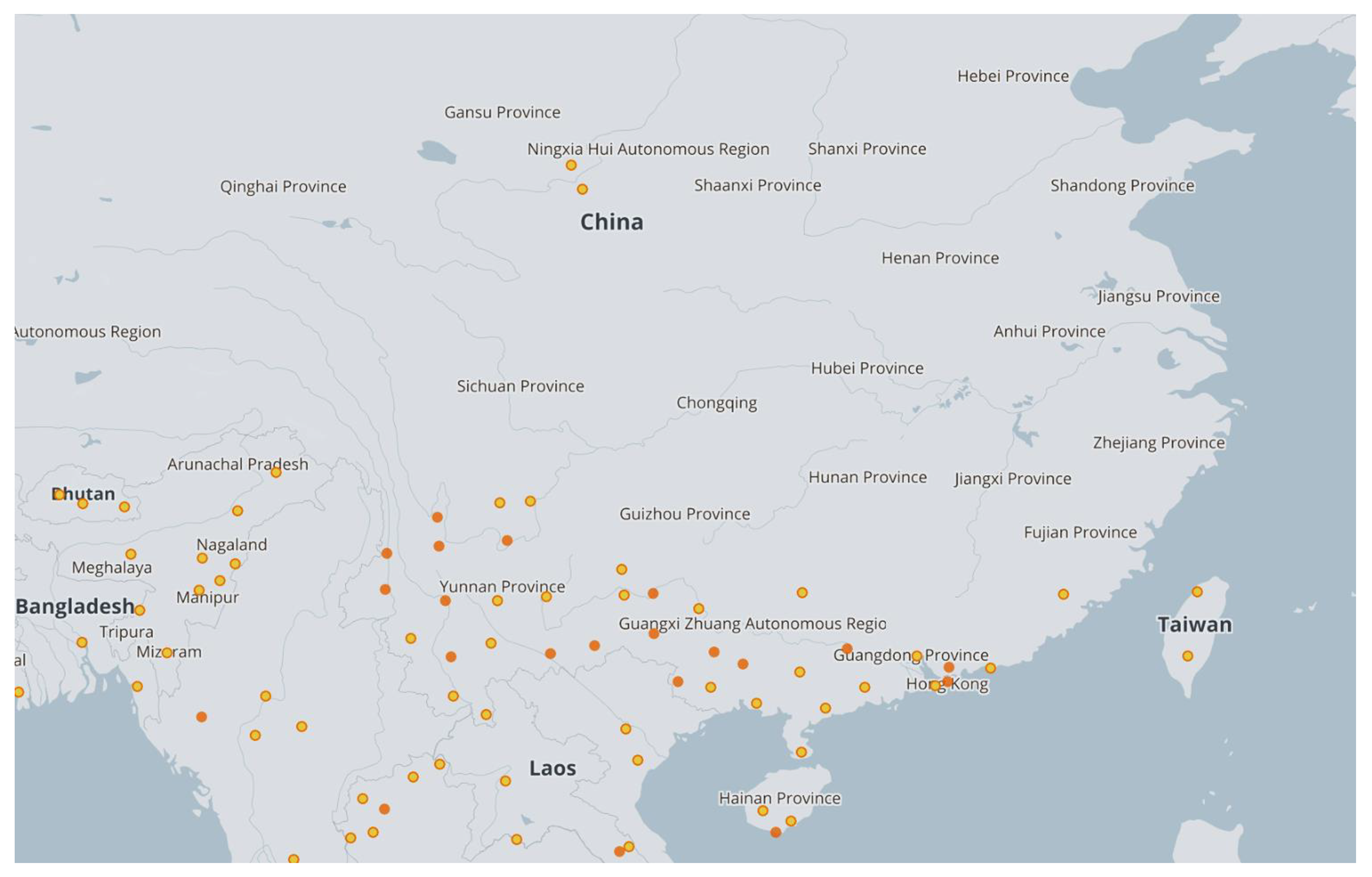

Supplement: Supplementary file 1 [file Image2.TIF]

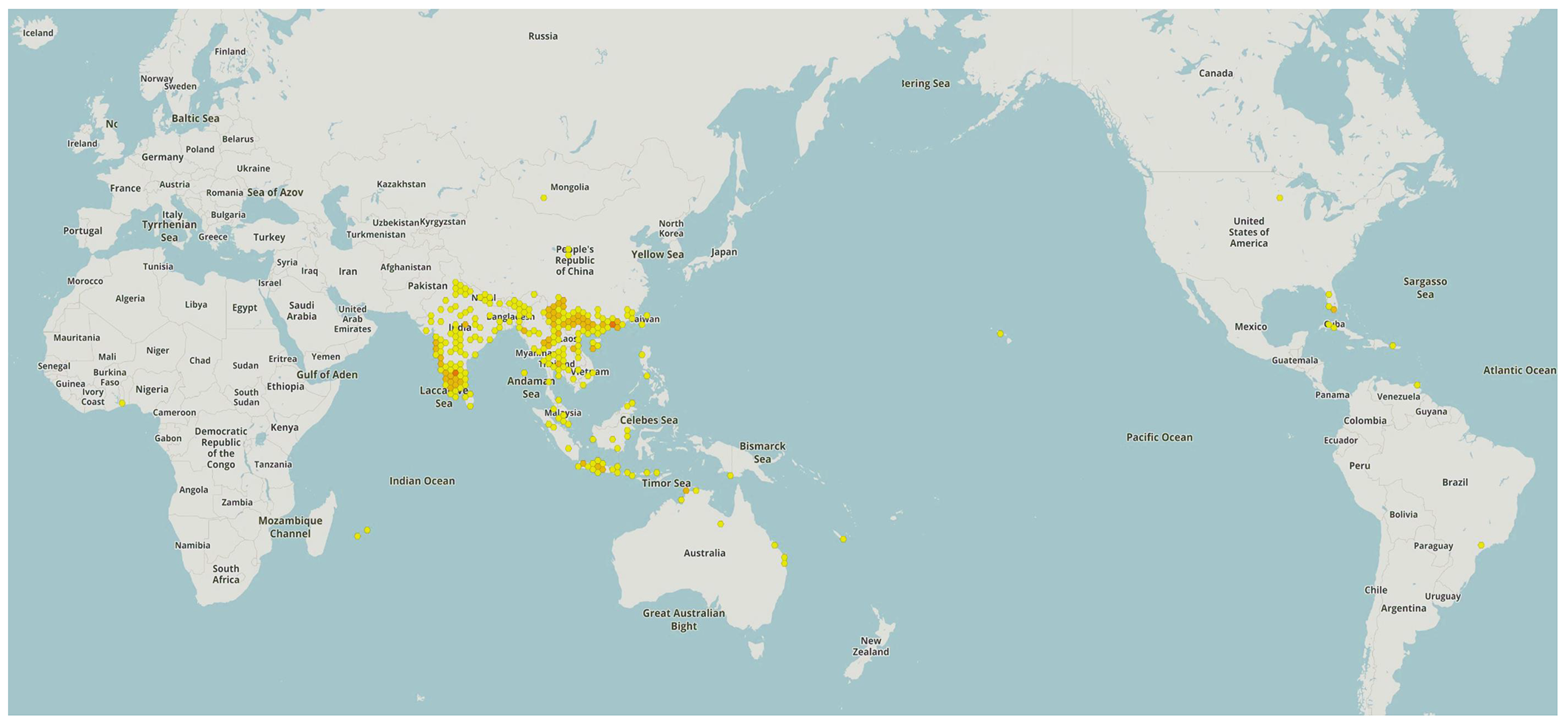

Supplement: Supplementary file 2 [file Image1.TIF]
